# The Invertebrate Lysozyme Effector ILYS-3 Is Systemically Activated in Response to Danger Signals and Confers Antimicrobial Protection in *C*. *elegans*

**DOI:** 10.1371/journal.ppat.1005826

**Published:** 2016-08-15

**Authors:** Maria João Gravato-Nobre, Filipa Vaz, Sergio Filipe, Ronald Chalmers, Jonathan Hodgkin

**Affiliations:** 1 Department of Biochemistry, University of Oxford, Oxford, United Kingdom; 2 School of Life Sciences, University of Nottingham, Queen's Medical Centre, Nottingham, United Kingdom; 3 Laboratory of Bacterial Cell Surfaces and Pathogenesis, Instituto de Tecnologia Química e Biológica and Faculdade de Ciências e Tecnologia, Universidade Nova de Lisboa, Lisboa, Portugal; Stanford University, UNITED STATES

## Abstract

Little is known about the relative contributions and importance of antibacterial effectors in the nematode *C*. *elegans*, despite extensive work on the innate immune responses in this organism. We report an investigation of the expression, function and regulation of the six *ilys* (invertebrate-type lysozyme) genes of *C*. *elegans*. These genes exhibited a surprising variety of tissue-specific expression patterns and responses to starvation or bacterial infection. The most strongly expressed, *ilys-3*, was investigated in detail. ILYS-3 protein was expressed constitutively in the pharynx and coelomocytes, and dynamically in the intestine. Analysis of mutants showed that ILYS-3 was required for pharyngeal grinding (disruption of bacterial cells) during normal growth and consequently it contributes to longevity, as well as being protective against bacterial pathogens. Both starvation and challenge with Gram-positive pathogens resulted in ERK-MAPK-dependent up-regulation of *ilys-3* in the intestine. The intestinal induction by pathogens, but not starvation, was found to be dependent on MPK-1 activity in the pharynx rather than in the intestine, demonstrating unexpected communication between these two tissues. The coelomocyte expression appeared to contribute little to normal growth or immunity. Recombinant ILYS-3 protein was found to exhibit appropriate lytic activity against Gram-positive cell wall material.

## Author Summary

Innate immune defenses against bacterial pathogenesis depend on the activation of antibacterial factors. We examined the expression and relative importance of a gene family encoding six invertebrate-type lysozymes in the much-studied nematode *C*. *elegans*. The *ilys* genes exhibit distinct patterns of tissue-specific expression and response to pathogenic challenge and/or starvation. The most abundantly expressed, *ilys-3*, exhibits constitutive pharyngeal expression, which we show is essential for efficient disruption of bacteria under non-pathogenic growth conditions, and consequently it contributes to normal longevity. *ilys-3* is also strongly up-regulated in intestinal cells after starvation or exposure to Gram-positive pathogens such as *Microbacterium nematophilum* and acts as a ‘slow-effector’ in limiting pathogenic damage from intestinal infections. We show that this induction by pathogens depends on the action of an ERK-MAPK cascade, which acts in pharyngeal rather than intestinal cells; this implies communication between pharynx and intestine. Tagged ILYS-3 protein was detected mainly in recycling endosomes of intestinal cells and in the intestinal lumen after starvation. ILYS-3 was also expressed in coelomocytes (scavenger cells) but we found that these cells make little or no contribution to defense. We examined the enzymatic properties of recombinant ILYS-3 protein, finding that it has lytic activity against *M*. *nematophilum* cell-walls.

## Introduction

Most animal epithelia possess innate defense systems that sense pathogenic and toxic insults and transmit stranger/danger signals to activate appropriate counter measures. The efficacy with which animals can respond to pathogens determines whether organismal homeostasis can be maintained and microbial clearance achieved. In vertebrates, the transcriptional programs that control the epithelial production of antimicrobials and mucosal homeostasis both act via coordinated activation of common innate immune receptors: Toll-like receptors (TLR), Nod-like receptors (NLR) and the cytosolic helicases RIG-1 and MDA5. Some of these also function as sensors of endogenous or exogenous damage-associated molecular patterns [1].

The invertebrate *C*. *elegans* has proved a valuable model to deconstruct biological processes that deal with the way animals detect danger signals and respond to life-threatening events such as toxic chemicals, DNA damage, metabolic stress and pathogens. Damage inflicted on nematode tissues can trigger a variety of functionally conserved molecular events aimed at limiting and repairing damage or sustaining viability while adverse conditions persist (reviewed elsewhere [2–6]).

This nematode is a bacterial feeder that spends much of its life in decomposing vegetable matter and depends on microbes as its source of food. To survive an environment rich in potentially damaging microorganisms, *C*. *elegans* has evolved an epithelial defense system that is able to carry out complex functions. Thus surface-exposed tissues such as epidermis [7], pharynx [8], intestine [8,9], vulva [10], and hindgut [11] are not simple physical barriers, but have all been shown capable of eliciting appropriate immune defense when exposed to potential pathogens. A crucial regulatory challenge to the nematode is the requirement for an adequate discrimination between real threats and innocuous edible food sources.

Much of what we know about the way this nematode is able to cope with pathogen insults comes from studies with intestinal infections. The response of intracellular signaling pathways within the intestine to microbial pathogens has been studied extensively in *C*. *elegans* and has been reviewed elsewhere [4,12]. Intestinal epithelia recognize and respond to extracellular pathogens by activating specific intracellular programs that include families of MAP kinases such as p38 and JNK, among others, that result in expression of candidate immune effectors that fight pathogens.

Predicted antimicrobial proteins elicited in *C*. *elegans* after immune challenge include among others, C-type lectins, CUB-like domain proteins and lysozymes. Many of them probably help the organism maintain intestinal homeostasis under normal intestinal growth conditions [13]. However, and except for the saposins *ssp-1* [14] and *ssp-5* [15,16], for all the others their critical effector function in antimicrobial defense remains to be demonstrated.

We have studied the response of *C*. *elegans* to a Gram-positive bacterium that adheres to the rectal and anal cuticle, *Microbacterium nematophilum* [17]. This nematode-specific pathogen has provided a means to analyze the complex biochemistry of the surface coat and the underlying cuticle [3,5,18–20] as well as innate immune responses. Orally ingested *M*. *nematophilum* alone instigates inflammation and tail swelling [21]. Despite the fact that the most obvious response to infection is rectal colonization and the induction of inflammation in the rectal tissues, this bacterium also establishes itself in the gut of the worm. This makes it a good system to investigate complex effects that occur in the digestive tract associated with chronic gut colonization.

We, and others, have previously found a cluster of invertebrate lysozymes that include *ilys-2* and *ilys-3*, (and the probable pseudogene *ilys-1)* is upregulated upon exposure to many different bacteria, namely *M*. *nematophilum* [22], *Staphylococcus aureus* ([8] and [23]), *Bacillus thuringiensis* DB27 [23], *Enterococcus faecalis* [24], *Cronobacter sakazakii* [25], *Salmonella enterica* serovar *Typhimurium* [26], *Photorhabdus luminescens* [27] as well as fungi *Candida albicans* [28] and *Drechmeria coniospora* [24].

Lysozymes are important bactericidal effectors present in phylogenetically diverse organisms from vertebrates to bacteriophages. In addition to promoting digestion, lysozymes confer direct broad-spectrum antimicrobial properties and perform essential functions in innate immunity. They are effective at targeting the cell wall of many Gram-positive bacteria by hydrolyzing the 1,4-β-glycosidic linkages between *N-*acetylmuramic acid and *N*-acetylglucosamine that make up the carbohydrate backbone of cell wall peptidoglycan. Although primarily associated with defense against Gram- positive bacteria, Gram-negative bacterial cell walls can also be attacked by these enzymes [29,30]. Based on their structural, catalytic and immunological differences, lysozymes have been classified into 6 sub-types. Major lysozymes found in animals belong to chicken(c), goose (g), invertebrate (i) or protist-type groups. All these four groups have been identified in invertebrates. *C*. *elegans* has numerous lysozyme genes belonging to both the protist-type (*lys-1* to *lys-10)* and invertebrate type (*ilys-1* to *ilys-6)* lysozymes reviewed by [31] and [32] and this study. Besides their ability to hydrolyse 1,4-β-glycosidic bonds in the glycan moiety of peptidoglycan, invertebrate lysozymes can also exhibit an isopeptidase (known as destabilase) activity, which specifically targets the isopeptide bonds established between D-Glutamate and L-Lysine present in some Gram-positive peptidoglycans [32–35]. An additional activity attacking isopeptide-type substrates can potentially enhance the lytic activity of invertebrate lysozymes. However, in most cases such synergy is still elusive and awaits further evidence.

While the protist-type lysozymes have been shown to play a role in host defense in interactions that involve *C*. *elegans* and *B*. *thuringiensis* [36], *Serratia marcescens* [37], *Salmonella typhimurium* [38], *S. aureus [8]* and *M*. *nematophilum* [22], little is known about the function of the i-type lysozymes in the worm. We therefore investigated expression, induction and function of *ilys-1-6* focusing our attention on ILYS-3.

Here we show that *C*. *elegans* invertebrate lysozyme ILYS-3 has important roles in both healthy and diseased worms; it is required to enable normal pharyngeal grinder function and to defend against bacterial pathogens. We find that ILYS-3 is a slow effector that is induced by danger signals generated both by bacterial pathogens and starvation. Under non-pathogenic conditions, in the gut *ilys-3* expression levels undergo a post-developmental regulatory oscillation. Levels increase after L1 hatching, decline after the L2 transition, and after L4 transition increase again becoming abundantly expressed in the intestine of adult worms.

Furthermore, we provide evidence that the *M*. *nematophilum*-mediated intestinal induction of *ilys-3* is regulated through the pharyngeal action of the extracellular signal-regulated MAP kinase (ERK) pathway in a non-cell- autonomous manner. This effect implies a mechanism whereby pharyngeal cells sense and propagate danger signals and further instruct antimicrobial responses in distal tissues. This systemic immune regulation of *ilys-3* reveals an unexpected communication between the pharyngeal and intestinal tissues in this multicellular organism.

Finally, using recombinant ILYS-3 fusion proteins we demonstrate their *in vitro* hydrolytic activity against the peptidoglycan of Gram-positive *Micrococcus luteus* and *M*. *nematophilum*. Such lytic activity will impact on the integrity of the cell walls and bacterial viability, which ultimately will affect proliferation in the nematode intestine. In addition, ILYS-3 probably contributes to the ability to digest and cope with the large amount of peptidoglycan fragments generated by a Gram positive diet (either pathogenic or non-pathogenic).

## Results

### ILYS genes are expressed in the pharynx, intestine and diverse tissues

**Fig 1:**
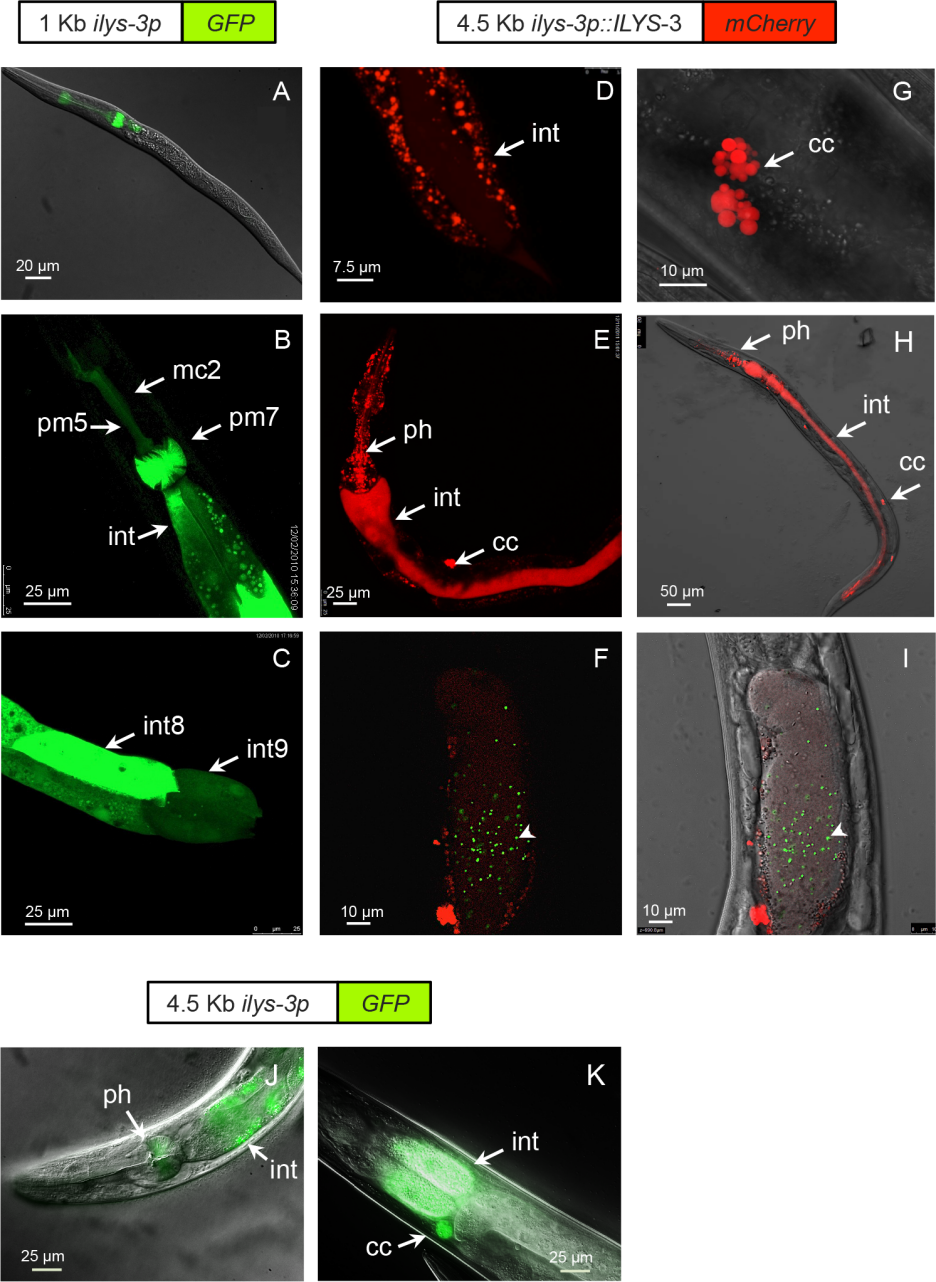
*C*. *elegans ilys-3* is expressed in the pharynx, intestine and coelomocytes. (A-C) GFP expression directed by the 1 Kb *ilys-3* promoter (upstream the start codon). (A) Example of expression of *ilys-3p* in L1. (B) The transcriptional reporter expresses strongly in the pharyngeal muscle pm7 (grinder), in the marginal cell mc2 and in the intestine of an adult worm. (C) Details of the variation in *ilys-3* transcription in intestinal int8 and int9 cells in an adult worm. (D-I) Expression of the *ily-3p*::*ILYS-3*::*mCherry* translational fusion reporter. (D) Distribution of ILYS-3-associated vesicles in the cytoplasm of intestinal cells in an adult hermaphrodite. The intestinal lumen is also visible. (E) Detail of a late adult (8 day after L4) showing mCherry in the intestinal lumen, pharynx and coelomocytes with copious mCherry positive puncta in pharynx and diffuse distribution in intestinal lumen. (F & I) Detail of a late adult worm fed with 0.5 μm yellow-green microspheres 1 hour before imaging showing accumulation of fluorescent beads (arrowhead) in the gut lumen together with faint ILYS-3::mCherry. (F) Fluorescence image and (I) overlay of a DIC image and the epifluorescence image shown in F. (G) Enlarged image showing coelomocyte expression of ILYS-3::mCherry. (H) Image of a starvation-induced-dauer showing high luminal gut expression of ILYS-3::mCherry. (J-K) Representative images of the 4.5 Kb *ilys-3* long promoter GFP construct in adult hermaphrodites. (J) Promoter activity in pharynx and intestine and (K) in the coelomocytes. ph:pharynx; int: intestine; cc: coelomocytes. Scale bars as indicated.

To provide insights into their site of action and response to infection, we investigated the expression pattern of the six invertebrate lysozymes present in the *C*. *elegans* genome. To this end we made transcriptional reporter fusions using DsRed2, CFP or GFP proteins (Fig 1 and S1 Fig). We found that all the invertebrate lysozymes have in common an intestinal expression pattern (Fig 1B and 1C and S1C Fig and S1E Fig, S1F–S1H Fig and S1K Fig and S1 Table). However, one subset appeared to have additional pharyngeal expression whereas the other exhibited some neuronal promoter activity. The four *ilys* genes *ilys-1*, *-2*, *-3* and *-6* exhibited distinct patterns of constitutive pharyngeal expression. *ilys-1* expressed in the pm3 cells in the procorpus, the pm4 cells in the anterior bulb pharyngeal muscles (sieve) and the marginal cells mc1 and mc2 (S1A Fig and Fig 1); *ilys-2* expressed in the pharyngeal muscle pm3 and in the nerve ring (S1B Fig); *ilys-6* in the pharyngeal gland cells and in the coelomocytes (S1I and S1K Fig). A detailed characterization of *ilys-3* expression is presented below. The second subset included *ilys-4* and *-5*, and revealed neuronal-specific expression pattern. S1D Fig shows the expression of *ilys-4* in a set of interneurons, the presumptive AVE/AVA and RIG/ALA cells that send processes that extend the entire length of the ventral and dorsal nerve cord respectively (S1E Fig and S1 Video). S1G Fig and S2 video depict the expression of *ilys-5* in interneurons in the head, the presumptive pharyngeal neuron I1 and AIM/AIY.

This analysis revealed an unexpected variety in the *ilys* expression patterns, suggesting that these genes may have distinct biological roles. Anatomically, expression of *ilys-1*,*-3* and *-6* covers the whole pharynx suggesting that they may have complementary functions. Given the considerable chemical, organizational and architectural diversity within bacteria cell walls and peptidoglycans (PGNs), one might expect little or no redundancy among *ilys* genes.

We found distinct allelic forms of *ilys-1* in various natural isolates of *C*. *elegans*. These included several exonic SNPs and a 10 bp insertion in the second intron that is likely not to impact on the coding region. Unlike all the other *ilys* genes, *ilys-1* does not encode a signal sequence, its first exon consists only of two methionines, and this together with the accumulation of several polymorphisms suggests that this invertebrate lysozyme is a pseudogene, despite its functional promoter.

The functional relevance of the neuronal expression of invertebrate lysozymes is unknown. However, lysozymes have been thought to play a role in neurodegenerative diseases as a part of amyloid plaque pathology. In a fly model of Alzheimer's disease for example, neuronal co-expression of lysozyme and amyloid-β_1–42_ diminished soluble and insoluble amyloid species, and prolonged survival of Aβ-expressing flies [39].

In this study, we focused our attention on the *ilys-3* gene because this was one of highly expressed genes induced in *C*. *elegans* upon exposure to *M*. *nematophilum* and its reporter showed strong induction under various conditions (as described below).

To determine its spatial distribution and responses to infection, we generated four expression reporters. Two of these consisted of a short (1.05 kb) and a long (4.5 kb) version of the *ilys-3* 5' sequence (presumed promoter) driving GFP expression. The third and fourth types were translational reporters with GFP or mCherry fused to ILYS-3 ORF at the N- or C-terminus, respectively. Both translational reporters used the 4.5 Kb *ilys-3* promoter.

The short promoter drove GFP expression mainly in the pharynx grinder muscles pm7, the isthmus marginal cell mc2 and muscle cell pm5 (Fig 1A and 1B). Grinder action is believed to disrupt bacterial cells. In addition, all intestinal cells exhibited *ilys-3* promoter activity, with some variability (Fig 1B and 1C). Intestinal expression of *ilys-3* appeared to be temporally regulated. GFP was first detected in intestine of L1 hatchlings but declined at the L2 transition. By the L4 stage, the intestinal GFP signal became stronger and continued through adulthood. The intensity of GFP was very high in aging adult worms that were no longer self-fertile. Whereas the intestinal expression of *ilys-3* changed during larval growth and adult life, the pharyngeal GFP reporter expression remained constant in larval and adult stages. Weak epidermal promoter activity was also detected.

The long promoter revealed additional GFP expression in the six scavenger coelomocyte cells, but at a significantly lower level in other tissues (Fig 1K). This reporter included only part of the 28 Kb sequence between *ilys-3* and its closest upstream neighbor, C45G7.4, but most of the region is occupied by a large cadherin gene *cdh-10*, on the other strand, whose 3' end is only 1.2 Kb from the predicted ATG of *ilys-3*.

N and C terminal tags showed essentially the same spatial expression patterns. However, higher levels of fluorescence intensity were seen in transgenic animals harboring extrachromosomal arrays of the C-terminal mCherry translational fusion, so this reporter was used in most subsequent experiments.

Steady-state levels of ILYS-3::mCherry were barely detectable until late L4 stage. When the animals entered adulthood, the mCherry signal became stronger in the pharynx, the intestine and in the coelomocytes. The epidermis also expressed mCherry albeit at low levels (S2 Fig).

Representative images of this expression are presented in Fig 1D–1I. ILYS-3::mCherry was abundant in the intestinal lumen in old adults (Fig 1E) and in animals subjected to starvation (Fig 1H). Such luminal secretion of ILYS-3 by intestinal cells was most conspicuous in dauers induced on nutrient-depleted plates. By contrast, we noted surprisingly little of the ILYS-3::mCherry signal in the intestinal lumen of animals under normal growth. To attain additional evidence that tagged ILYS-3 accumulates in the intestinal lumen, we fed animals with fluorescent microspheres just prior to their imaging. As shown in Fig 1F and 1I ingested microspheres accumulated in the intestinal lumen marked with red fluorescence.

The translational mCherry fusion protein shows a punctate expression pattern in most tissues in the pharynx and intestine (Fig 1D and 1E). In the intestinal cells, ILYS-3::mCherry positive vesicles were enriched in the cytosol.

Together these data show that ILYS-3 has a dynamic expression pattern under standard growth conditions and is found primarily in the digestive tract of *C*. *elegans* in the pharyngeal cells and the intestine, indicating that it may play roles both in early stages of bacterial grinding and in further lysis required for nutrient digestion.

### ILYS-3 constitutively localizes to intestinal vesicles and is secreted in the lumen at dauer arrest

**Fig 2:**
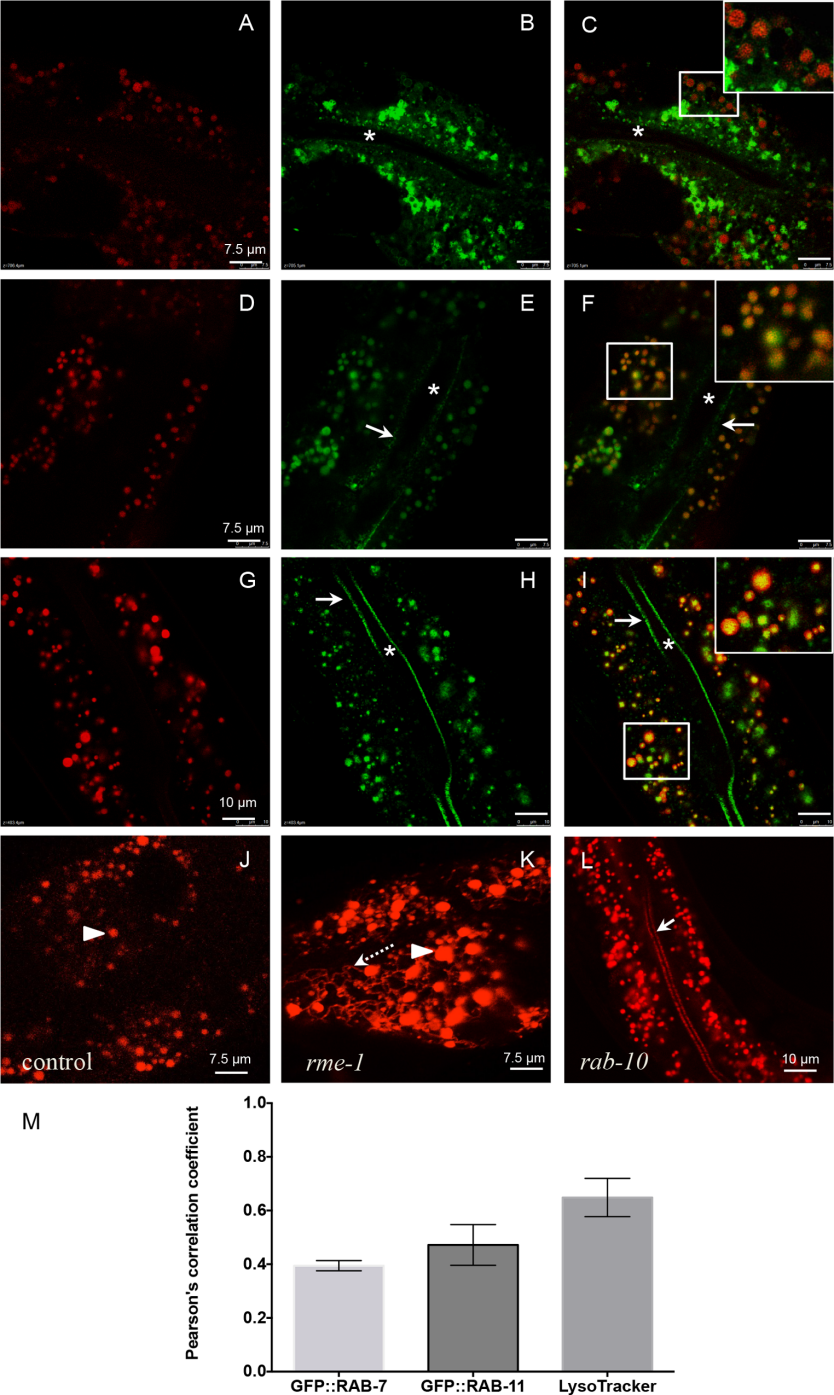
Intestinal ILYS-3::mCherry associates with endocytic and recycling endosomes and LROs. (A-C) Confocal images of the middle focal plane of the intestine showing that ILYS-3::mCherry partially colocalizes with a subpopulation of GFP::RAB-7 in the median cytoplasm in late endosomes, labeled by both ILYS-3::mCherry (A) and GFP:RAB-7 (B). (C) Overlay of the corresponding red and green channel, magnified 3x region in inset. (D-F) ILYS-3::mCherry occasionally appears in RAB-11 positive vesicles residing in the middle focal plane of the intestine. No double labeling is seen on the apical side of the intestine. (D) ILYS-3::mCherry. (E) GFP::RAB-11. (F) Overlay of the corresponding red and green channel, magnified 3x region in inset. (G-I) ILYS-3::mCherry colocalizes with LysoTracker Green in the intestinal lysosome-related organelles (LROs). (G) ILYS-3::mCherry. (H) LysoTracker Green is internalized apically by the intestinal cells and accumulates in the acidic LROs (green granules). Inset in (I) shows the doubly marked LROs. No red signal is seen in the apical side of the intestine. (J-K) Images of the top focal plane showing the basolateral compartment of the intestine. (J) Micrograph acquired underneath the basolateral membrane showing a population of ILYS-3::mCherry positive vesicles in the cytosol in a wild-type control. (K) ILYS-3::mCherry accumulates in the recycling endosome tubular network in the *rme-1* mutant. (L) ILYS-3::mCherry accumulates apically in the plasma membrane and in puncta in *rab-10* mutants. Micrograph acquired in the middle focal plane of the intestine. Asterisks depicts the lumen of the intestine. Arrows point to the apical membrane. Arrowheads point to intestinal vesicles. Dashed arrow marks basolateral tubular network mCherry-labeled. (M) Pearson's correlation coefficient for colocalization of ILYS-3::mCherry signal with GFP::RAB-7, GFP::RAB-11 and LysoTracker Green. Confocal images were from deconvolved 3D stacks acquired in living adults expressing mCherry- and GFP-tagged proteins in the intestinal epithelial cells. Autofluorescence was corrected using Leica LAS X core software. Values for each group represent n = 17/21 areas from 8 animals. Error bars represent SEM.

Given the unexpected paucity of the ILYS-3::mCherry signal in the intestinal lumen of young animals, we examined the subcellular trafficking of ILYS-3. For this, we used well-established markers for early endosomes (GFP::RAB-5), late endosomes (GFP::RAB-7), and Trans-Golgi Network (TGN) and apical recycling endosomes (GFP::RAB-11) (Fig 2). The intestine of *C*. *elegans* is a polarized epithelium with apical membranes and microvilli facing the lumen and a basolateral surface contacting the body cavity (pseudocoelom).These two domains are separated by apical junctions. Molecules that reach the lumen are believed to be delivered through complex sorting machineries in four distinct ways: 1. directly, 2. indirectly, via specifically dedicated vesicles, 3. via apical recycling endosomes or 4. through transcytosis by means of membrane-bounded carriers.

As shown in Fig 2 only a subpopulation of ILYS-3::mCherry colocalized and appeared as puncta around the rim of the GFP::RAB-7 labeled late endosomes (Fig 2A–2C). This population of mCherry-positive vesicles was distributed through the cytoplasm, in the middle focal plane. We found a Pearson's correlation coefficient of 0.39, indicating a low degree of colocalization with the total population of RAB-7-positive endosomes. Some of the RAB-11-positive recycling endosomes that appeared scattered in the cytoplasm were also mCherry positive. Pearson's coefficient at 0.47, suggested some degree of correlation. However no ILYS-3::mCherry signal could be detected at the apical membrane and its associated puncta (Fig 2D–2F). Likewise, no mCherry and GFP-double labeling was detected with the transmembrane marker for the apical intestinal membrane OPT-2 [40].

A separate but pertinent population of ILYS-3-positive vesicles was associated with the acidic lysosome-related organelles (LROs) in the intestine and were LysoTracker Green-labelled (Fig 2G–2I). The Pearson's coefficient was 0.62, implying a greater degree of correlation of ILYS-3 signal with these organelles. LROs are destination vesicles for degradation of extracellular macromolecules from both the apical and basolateral trafficking routes. Fig 2I illustrates also the lack of ILYS-3::mCherry in the apical membrane in contrast to the positive green-labelled-LysoTracker, internalised from apical uptake.

At its simplest, the surprising absence of strong apical and luminal mCherry signal in young animals could indicate that these animals simply degrade the tagged ILYS-3 more efficiently than older siblings. However, the presence of the ILYS-3:mCherry in endosomal and LRO compartments suggests that there could be fast turnover and/or re-uptake of the luminal ILYS-3 through an endocytic recycling route. Hence, the protein could still be present in the apical compartments, but below thresholds of detection.

Endocytic recycling of proteins from endosomes to the plasma membrane is important in cellular processes such as nutrient uptake and immune functions [41]. We next sought to determine whether the reason why we were unable to detect apical mCherry was because ILYS-3 was associated with endosomes that perform basolateral recycling. We used the *rme-1* mutant that specifically accumulates abnormally high numbers of basolateral recycling endosomes but does not accumulate increased numbers of early, late, or apical recycling endosomes in their intestines [42]. In the *rme-1* mutants a large number of ILYS-3::mCherry vesicles associated with a basolateral tubule-vesicular network were detected (Fig 2K) relative to WT background (Fig 2J). Together these results suggest that at least part of ILYS-3::mCherry associates with the interconnected endosomal tubules and that basolateral recycling of ILYS-3 does not require RME-1. Rather than being actively delivered to the apical plasma membrane domain, ILYS-3::mCherry appeared to be dispatched, via the basolateral route, to recycling endosomes. We also found that *M*. *nematophilum* infection induced high ILYS-3::mCherry signals in the tubular-vesicular recycling endosome network of the intestinal cells, strongly resembling animals lacking RME-1 (S3 Fig). The basolateral recycling endosomal location of ILYS-3 was surprising given that abundant ILYS-3::mCherry was observed in the lumen of old animals and dauers.

RAB-10 has been shown to function in basolateral recycling in the intestine upstream RME-1 [43]. *rab-10* mutants exhibit enlarged early endosomes that are likely to play roles in receiving, sorting and also recycling cargo received from both the apical and the basolateral domains. We therefore tested whether a defect in such key endocytic route could interfere with the distribution of basolateral-destined ILYS-3::mCherry. In *rab-10(ok1494)* deletion mutants we observed an aberrant accumulation of the red signal on the apical membrane and its associated vesicles of the intestine (Fig 2L). This was consistent with an impairment on basolateral recycling that characterizes this genetic background. We interpret these results, as an indication that ILYS-3 can be routed to the apical side of the intestinal cells, albeit at low levels in WT backgrounds.

**Fig 3:**
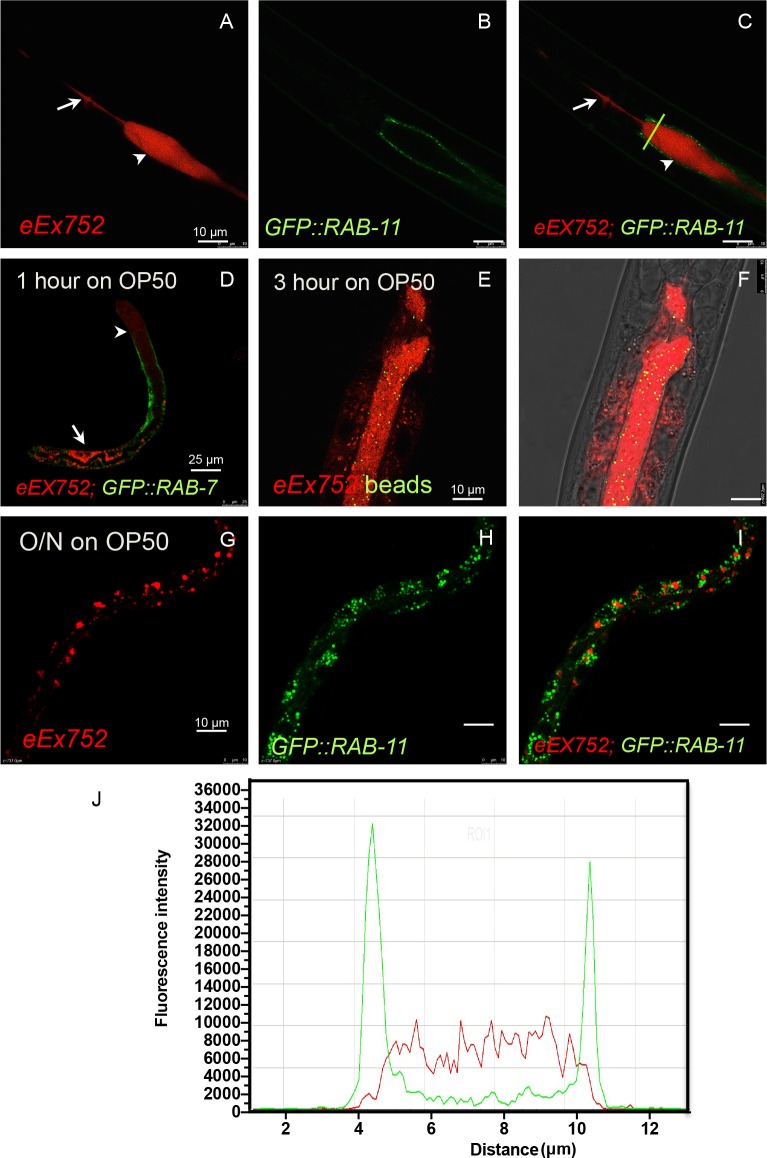
During dauer arrest ILYS-3 is secreted in the lumen but returns to its steady state cytosolic expression upon dauer recovery. (A-I) Time course of ILYS-3 intestinal distribution in dauers. (A-C) Fluorescence images of ILYS-3::mCherry (A), GFP::RAB-11 labeling apical recycling endosomes in plasma membrane (B) and an overlay (C) of the two images acquired with red and green channels. Images are representative of 1-week old dauers yielded from nutrient depleted NGM plates. Arrow and arrowhead depict the pharyngeal and the intestinal lumens, respectively. (D) Micrograph of a dauer animal recovering after 1 hour on OP50. Red depicts ILYS-3::mCherry and green depicts GFP::RAB-7 that marks early endosomes near PM and late endosomes in cytoplasm. Animal shows luminal (arrowhead) and cytosolic (arrow) ILYS-3 at the anterior and posterior ends, respectively. (E-F) Micrographs of a dauer animal recovering after 3 hours on OP50. Red depicts ILYS-3::mCherry in lumen and green depicts fluorescent beads added to the bacterial lawns. (F) overlay of the two channels. (G-I) Micrographs of post-dauer animal recovering after overnight on OP50. (G) Red signal is only detected in vesicles in the cytosol. (H) GFP::RAB-11 in puncta scattered in the cytoplasm. (I) overlay. (J) The mean fluorescence intensity profile corresponding to the animal shown in images A-C. mCherry and GFP containing regions have little overlap and the red signal is extracellular. The green dashed line in (C) indicates the cross section used to quantify fluorescence.

As described above, only in old adults and dauer larvae do we see abundant luminal accumulation of ILYS-3::mCherry. The simplest explanations for this are that the turnover of the protein is impeded in intestinal epithelial cells of these animals and/or it resulted from the leakage due to apical cell damage. Tight junctions act as fences, preventing leakage of basolateral components into the apical compartment. However, if their integrity is compromised, leakage may ensue. While these two scenarios seemed possible in old animals, they appeared unlikely in dauers. To address this issue we first examined where ILYS-3 accumulated in dauers. For this, we co-expressed ILYS-3::mCherry and the intestinal marker RAB-11 tagged to GFP, which labels the apical plasma membrane. As shown in Fig 3A–3C and Fig 3J, most of red signal conferred by ILYS-3::mCherry accumulated within the extracellular milieu, i.e. in the pharyngeal and intestinal lumina. In the intestine mCherry was detected between cells marked by GFP::RAB-11 and the intensity profiles of the two proteins have little overlap (Fig 3J). Steady levels of high luminal ILYS-3::mCherry could still be observed in 25-day old dauer larvae, suggesting that the protein was not actively degraded in these animals.

Strikingly, ILYS-3::mCherry expression pattern in the lumen of dauers rapidly returned to the original localization when animals resumed development after re-exposure to OP50. As illustrated in Fig 3D mCherry began to accumulate intracellularly even before animals resumed feeding in the first hour after they encountered food. The figure is representative of a recovering dauer larva transferred to a bacterial lawn mixed with fluorescent microspheres. No fluorescent beads were detected in the intestinal lumen, indicative of no feeding activity. The transgenic animal shown co-expressed ILYS-3::mCherry and the GFP::RAB-7, a marker for early endosomes near plasma membrane and late endosomes deeper in the cytoplasm. Red fluorescence signal was seen in the lumen and the cytosol at the most anterior and posterior intestinal cells, respectively. Three hours later, fluorescent beads denoting feeding activity could be found in the intestinal lumen, where mCherry was still highly abundant (Fig 3E and 3F). Upon overnight feeding the punctate expression pattern of ILYS-3::mCherry in the cytosol was re-established (Fig 3G–3I).

Taken together these data show that the vesicular and luminal ILYS-3::mCherry distribution is modifiable and regulated by nutritional conditions. This invertebrate lysozyme is secreted in the intestinal lumen but it can also be detected deeper in the cytosol in LROs and recycling-associated vesicles, both of which are known final destinations for internalized fluid-phase cargos. Our observations suggest an intracellular role for the *C*. *elegans* ILYS-3 in addition to luminal activity, which includes intracellular intestinal compartments where it could be acting to degrade residual peptidoglycan fragments.

### *ilys-3* is activated in the intestine in response to bacterial pathogens and hunger signals

Previous microarray analysis showed that during early infection by *M*. *nematophilum*, *ilys-2 and ilys-3* were upregulated by 3 and 4- fold, respectively [22]. To further investigate the role of *ilys-3* gene in host-pathogen defense, we visualized *ilys-3* transcriptional activation *in vivo* using L1 transgenic worms expressing the GFP reporter transgene (short *ilys-3* promoter). We used this promoter because the corresponding transgene showed stronger inducibility than the longer version.

**Fig 4:**
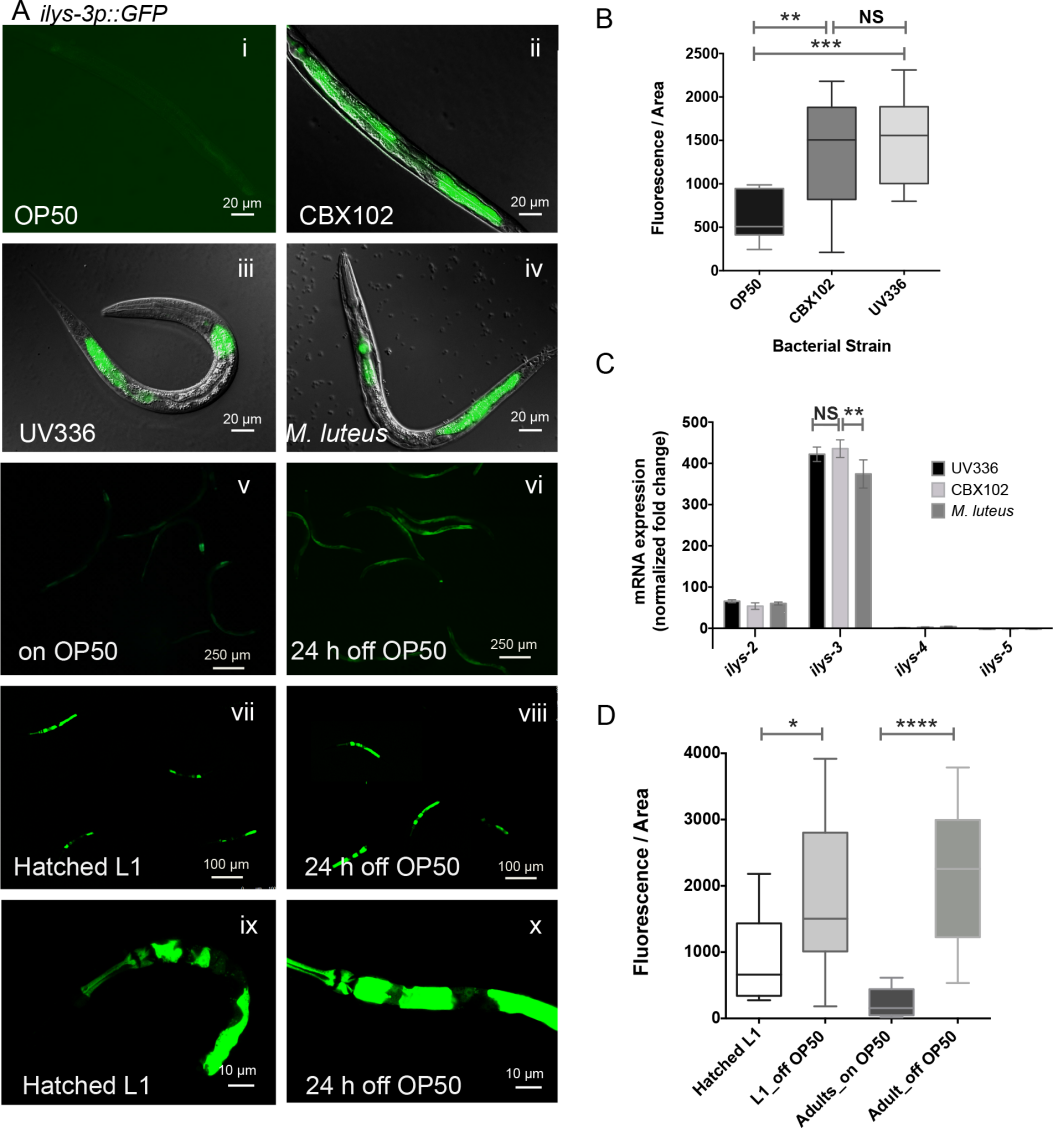
The *C*. *elegans ilys-3* is transcriptionally activated in the intestine upon Gram-positive exposure and is a readout for monitoring danger/hunger signals. (A) Fluorescence images of representative animals carrying the 1 Kb *ilys-3p*::*GFP* transgene following exposure to Gram-positive bacteria. (i) The basal expression of *ilys-3* in the intestine of *E*. *coli* (OP50) fed worms is hardly detected. (ii) Enhanced expression of GFP is observed in animals grown on 100% lawns of the virulent (swelling) and (iii) the attenuated strains of *M*. *nematophilum*, CBX102 and UV336, respectively. (iv) High GFP signal is also detected in the gut of animals exposed to *M*. *luteus*. (v) *ilys-3* reporter transgenic young adults grown on OP50. (vi) Activation of *ilys-3* transcription in the gut of young adults off food for 24 hours. (vii) Expression of the *ilys-3* reporter in L1 larvae at hatching and (viii) in arrested-L1s obtained from nutrient-depleted plates 24 hours after hatching. (ix) and (x) Representative images of the *ilys-3* expression in an L1 at hatching and an one-day-old arrested L1. (B) Quantification of the *ilys-3p*::*GFP* fluorescence in the intestinal cell int8 of the *ilys-3* reporter in animals grown on OP50, CBX102 and UV336. Shown are box plots distributions for the GFP expression in the intestinal cell of L1 animals maintained on the three bacteria for 48 hours at 25°C. The focal plane with the highest GFP signal was used to measure fluorescence intensity within a region of interest (ROI) set to 40 μ diameter and 0.4 μ thickness. Graph is representative of two independent experiments. Asterisks indicate the results of Mann Whitney test of fluorescence values, 95% confidence interval relative to OP50 of worms on CBX102 (** *p* = 0.0012), and on UV336 (*** *p* < 0.0001). NS: not significant. N = 15 per group. (C) qRT-PCR analysis of *ilys* genes from L1 larvae propagated on OP50, CBX102, UV336 for 48 hours, showing high levels of *ilys-2* and *ilys-3*. Expression levels were normalized to OP50, and to the endogenous control gene *rla-1*. *ilys-2* and *ilys-3* transcripts were clearly responsive to Gram-positive bacteria. In contrast, *ilys-4* and *ilys-5* mRNA levels remained mainly unchanged. Data were analyzed with two-way Anova, Holm-Sidak's multiple comparison tests (99% CI) and showed that the increased levels of induction of *ilys-3* mRNA by CBX102 and *M*. *luteus* were significantly different (** *p* = 0.0059) but there was no statistically significant change (NS) between animals on CBX102 and UV337 (*p* = 0.4644). Gene expression was analyzed using the comparative ΔΔCt method. Data are representative of four independent experiments. Error bars are SEM. (D) Quantification of the *ilys-3p*::*GFP* fluorescence in the intestinal cell int2 of the *ilys-3* reporter in animals subjected to nutrient depletion. Shown are box plots distributions for the GFP expression in the intestinal cell of hatched L1 larvae, one day-old arrested L1s, and young adults 24 hours after they were removed from food. Graph is representative of two independent experiments. Mean values for one-day-old adults off food differ significantly from their sibling controls on OP50 (**** *p* < 0.0001). Statistically significant differences were also seen in one-day-old arrested larvae (off OP50) when compared to naïve animals hatched overnight (* *p* = 0.0237). N = 12/group. Asterisks indicate the results of Mann Whitney test of fluorescence values, 95% confidence interval.

We found that *ilys-3* is highly responsive to immune challenges by different Gram-positive bacterial pathogens. Under steady state conditions, when animals develop on lawns of *E*. *coli* OP50 (Fig 4Ai), only low levels of GFP expression are detected in the gut. In contrast, 48 h after exposure to both virulent and attenuated strains of *M*. *nematophilum*, CBX102 (Fig 4Aii) and UV336 (Fig 4Aiii) or to *M*. *luteus* DMS20030 (Fig 4Aiv), strong GFP expression is induced in the intestine, irrespective of whether these pathogens induce an inflammatory swelling response in the worm rectum. The increased levels of GFP signal were quantified by measuring the fluorescence intensity of the intestinal cell int8 in individual transgenic worms that express the *ilys-3* reporter (Fig 4B). Single-cell measurements revealed a 3-fold induction of *ilys-3*::*GFP* in response to *M*. *nematophilum* CBX102 and UV336.

To quantify the pathogen-induced activation of *ilys-3* gene transcription and in non-transgenic animals we used quantitative real-time polymerase chain reaction (qRT-PCR) analysis of whole worm extracts, a method that allows a more reliable quantification of endogenous *ilys-3* mRNA than reporter genes. Strong induction of *ilys-3* (between 370 and 590x) was observed after animals were challenged for 24h (S4 Fig) and 48h by the three Gram-positive bacteria strains, CBX102, UV336 and *M*. *luteus* (Fig 4C).

A clear increase in transcriptional response (above 50x) was also seen for *ilys-2* and less markedly for *ilys-6 (*above 10x, S4 Fig), whereas for *ilys-4* and *ilys-5* no significant changes were observed. Neither *ilys-2*,*-3* nor*-6*, were changed in expression levels after exposure to the moderately lethal pathogen *P*. *aeruginosa* PAO1 (S4 Fig).

*M*. *nematophilum* CBX102 causes a distinctively cell swelling in the tail and infected animals become constipated 24 hours after feeding on this bacterial pathogen. In contrast, worms feeding on the attenuated strains *M*. *nematophilum* UV336 or *M*. *luteus* DMS20030 appear superficially healthy, but their rate of development is slower compared to *E*. *coli* OP50 controls, and they take one extra day to reach adulthood.

We hypothesized that the activation of *ilys-3* following bacterial exposure could be caused by a stress response due to nutrient deprivation resulting from low quality food ingested by the worms. To test this we monitored the levels of expression of *ilys-3* GFP reporter (GFP driven by the short promoter) in animals subjected to starvation. A 24 hour-starvation regime in young adults (Fig 4Avi) or in arrested L1 larvae (Fig 4Aviii and 4Ax) provoked a significant intestinal induction of the GFP signal (Fig 4D). Nutrient depletion led to a 14.5-fold and a 2.3-fold increase in the intestinal GFP signal detected in adults and larvae, respectively. The addition of live *E*. *coli* OP50 (Fig 4Av), [or HB101 (even better quality food, as reported by Shtonda & Avery [44]) restored the low levels of basal GFP expression of *ilys-3* in the intestine.

A strong GFP expression of the *ilys-3* reporter was also seen in animals fed on diets of heat-killed OP50, or heat-killed CBX102 (S5A and S5C Fig). Heat-killed bacteria are more likely to be harder-to-eat and to provide a bad quality diet. In fact, at 72 hours post-hatching all animals on dead bacterial cells had only reached the L2-L3 transitional larval stage. The response elicited to dead bacteria therefore represents a starvation rather than a pathogen response.

The starvation-induced GFP pattern was specific to the *ilys-3* reporter lines as all the other *ilys* transgenes failed to show induction when worms were starved for 24 hours. In fact the *ilys-5* reporter was repressed whereas transgenic animals expressing *ilys-2* and *ilys-4*, CFP or GFP levels remained unchanged. The strong GFP signal present in the intestine of *ilys-5* transgenic animals was completely abolished when animals were starved for 24 hours (S6 Fig).

Taken together, our results indicate that *ilys-3* activation is responsive to both Gram-positive bacteria and starvation signals. Although a previous microarray analysis identified a rapid up-regulation of ILYS-3 in response to *M*. *nematophilum* [22], our real-time PCR analysis revealed a much larger delayed increase of mRNA levels 24 and 48 h after bacterial challenge (590 and 430x, respectively).

### ILYS-3 is required in the pharynx and intestine to promote bacterial lysis and to confer protection against bacterial pathogens

To investigate the function of the *ilys-3* gene we analyzed the phenotype of the recessive mutation *ok3222*, which has an 855 bp deletion that removes most of the coding region. On standard bacterial food OP50 the mutants appeared superficially healthy, with no gross abnormalities and with fertility (self-progeny brood size) only slightly reduced relative to wild-type animals (S7 Fig).

**Fig 5:**
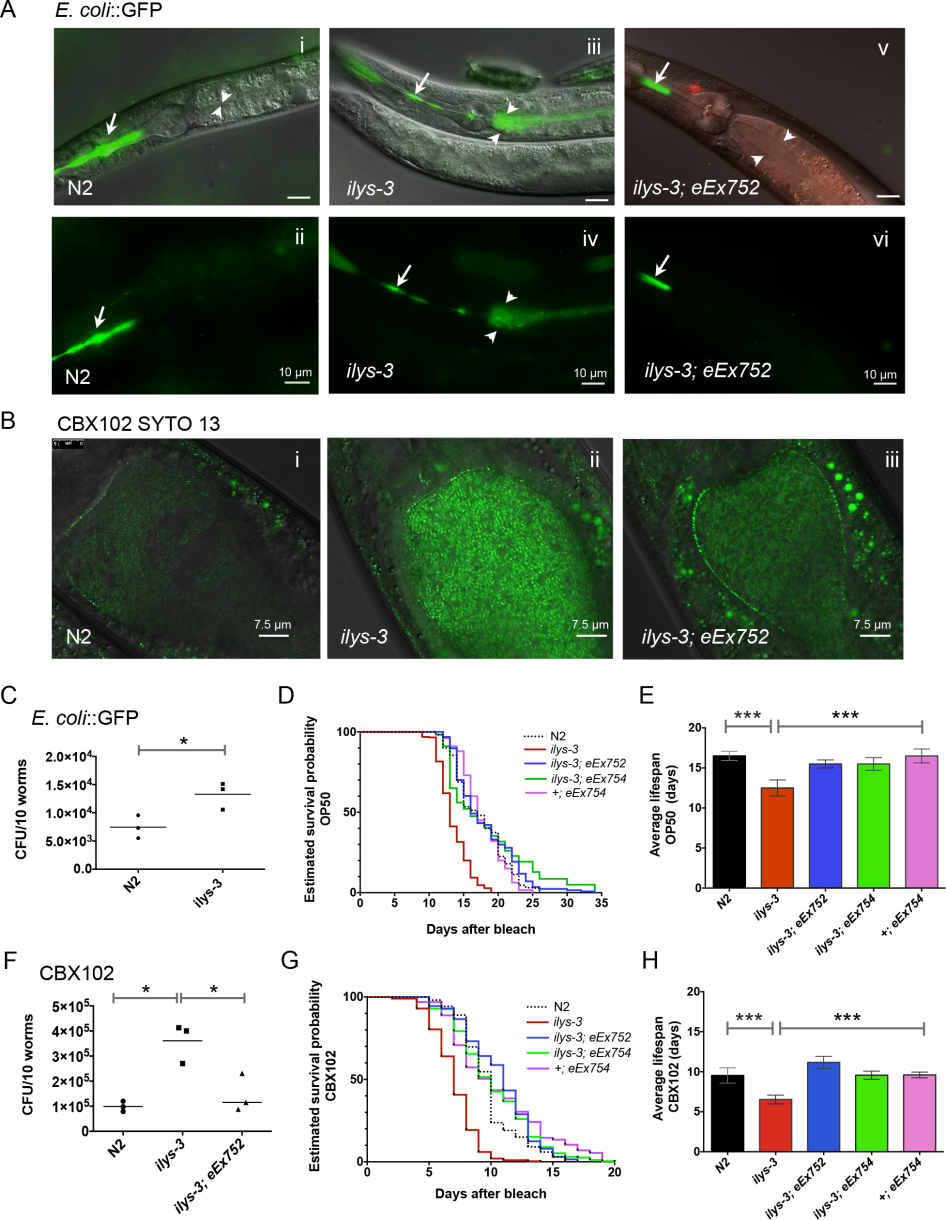
*ilys-3* is required in the pharynx and in the intestine to prevent bacterial burden in the gut lumen and to protect against *M*. *nematophilum*. **2.** (A) Images of a wild-type, *ilys-3* and *ilys-3; eEx752* one-day old adults fed for 24 hour on *E*. *coli* expressing GFP. Live bacteria cells are seen in the pharyngeal (arrow) but not intestinal lumen (arrowhead). (i-ii) N2. (i) Composite DIC and GFP fluorescence image. (ii) Green channel. (iii-iv) *ilys-3* deletion mutants accumulate live bacteria in the gut lumen and exhibit impaired ability to disrupt bacteria. (iii) Composite DIC and GFP fluorescence image (iv) Green channel. (v-vi) Overexpression of ILYS-3 in *ilys-3* with *eEx752* array rescues luminal bacterial accumulation in an animal of the same chronological age. (v) Composite DIC and GFP fluorescence image. (vi) Green channel. (B) Overlays of DIC and epifluorescence images of one-day-old adults of WT, *ilys-3* or *ilys-3; eEx752* exposed to SYTO 13-labeled CBX102 cells for 2 hours. (i) Fluorescence image of an N2 animal showing few stained CBX102 cells, indicative of non-viable bacteria. (ii) Gut lumen of an *ilys-3* animal with high accumulation of live CBX102 cells that fluoresced bright green due to SYTO 13. (iii) *ilys-3; eEx752* transgenic displaying reduced luminal bacterial accumulation. (C) The effect of *ilys-3* knockout on passage of live bacteria into the gut lumen. Bacterial load was calculated using a colony-forming units (CFU) count assay. N2 and *ilys-3* mutants were exposed as L4 larvae to *E*. *coli*::GFP for 24 hours. Each symbol represents the average bacterial load obtained from pools of 10 animals. Thick horizontal bars represent the median of three independent experiments (n = 270 animals/ group analyzed). Asterisk indicates the results of a two-tailed unpaired t-test, with Welch's correction, comparing values of colony forming units/10 worms on *ilys-3* versus N2 (* *p* = 0.0338, 95% CI). (D- E) Effect of ILYS-3 overexpression on survival rates of OP50-fed N2, *ilys-3(ok3222)*, *ilys-3; eEx752*, *ilys-3; eEx754*, and +; *eEx754* cultured at 20°C. *P* value *vs* control calculated with the Mantel-Cox log-rank test (95% CI). Results are the mean of 3 independent experiments with an average of 100 animals analyzed each time. Data in bar graphs depict means ± standard deviation. (D) Lifespan analysis showing that ILYS-3 overexpression extends lifespan in *ilys-3* mutants. (E) Average lifespan plot showing that the decreased average lifespan of *ilys-3* deletion mutants is restored to WT levels in ILYS-3 overexpressing animals carrying *eEx752* or *eEx754* arrays (*** *p <* 0.0001). (F) Counts of CFU isolated from one-day old adult animals, fed for 24 hour on CBX102. Each symbol represents the average bacterial load obtained from three biological replicates. Asterisk indicates the results of a two-tailed unpaired t-test, with Welch's correction, comparing values of CFU/10 worms on *ilys-3* versus N2 (* p = 0.0232) and *ilys-3* versus *eEx752* (* *p* = 0.0269), with a statistical confidence *p* value of <0.05 for each of the three repeats. (G-H) Effect of ILYS-3 overexpression on survival rates of N2 and *ilys-3*, upon exposure to CBX102. Transgenes used were *eEx752* or *eEx754*. *P* value *vs* control calculated with the Mantel-Cox log-rank test (95% CI). Results are the mean of 3 independent trials. Data in bar graphs depict means ± standard deviation. (G) Lifespan curves. (H) Loss of *ilys-3* decreases lifespan in animals exposed to CBX102, but ILYS-3 overexpression enhances their survival during infection by this pathogen.

If *ilys-3* is expressed in the pharyngeal tissue because it is required for efficient lysis then the mutant might be expected to show signs of defective grinder activity and consequently accumulate live bacterial cells in the gut. To address this possibility we compared the kinetics of bacterial accumulation in the gut of mutant and WT animals of the same age. L4 animals were exposed to GFP-expressing *E*. *coli* and SYTO 13-labeled CBX102 and visually scored at different times for presence of a green signal in the intestinal lumen. Although intestinal *E*. *coli* can be normally detected in old WT animals [45,46], they are rarely seen in the younger animals used in this experiment indicating efficient bacterial lysis. In contrast, we observed that *ilys-3* mutants accumulated substantially more un-lysed *E*. *coli*::GFP positive cells in their guts relative to WT (Fig 5Aiii–5Aiv and 5Ai and 5Aii). These differences were clear within four hours of feeding on GFP-expressing *E*. *coli*. Furthermore, an even higher intestinal bacterial load was observed when *ilys-3* mutants were challenged with CBX102 labeled with SYTO 13 (Fig 5Bii and Fig 5Bi). SYTO 13 is a cell-permeant nucleic acid stain that permits visualization of unlysed live bacterial cells. The high green fluorescence of CBX102 cells seen in the lumen of *ilys-3* mutants suggests that most bacteria that have passed through the pharyngeal grinder remain viable.

Bacterial survival in the alimentary tract was also measured directly by homogenizing washed whole animals and counting colony-forming units. In one-day old adults CFU numbers of *E*. *coli* in *ilys-3* mutants were two-fold higher than WT controls [note that these counts include bacteria from both pharyngeal and gut lumen, and therefore underestimate the difference in intestinal burden] (Fig 5C). The intestinal proliferation of CBX102 was also assayed in animals of the same age and we found that *ilys-3* mutants had significantly higher bacterial loads than WT (Fig 5F). We estimated that mutant animals had around 3.6-fold more cell counts than WT. Restoration of ILYS-3 in *ok3222* mutants using the transgene e*Ex752* rescued this phenotype and transgenic animals were able to grind bacteria more efficiently (Fig 5Av and 5Avi, Fig 5Biii and Fig 5F). In addition, the accumulation of viable CBX102 cells in *ilys-3* transgenic animals carrying the *eEx752* array was comparable to that observed for WT (*p* = 0.4090).

We therefore concluded that ILYS-3 is required in the pharynx for efficient disruption of live bacteria and to reduce bacterial colonization in the intestine of *C*. *elegans*.

Standard *E*. *coli* OP50 is commonly used as food despite the fact that it can be mildly pathogenic and its accumulation in the intestine is responsible for much variability in the lifespan of *C*. *elegans* [47]. Given this, we compared the life expectancy of wild-type and *ilys-3* null mutant strains grown on OP50 and *M*. *nematophilum* lawns.

Under our experimental conditions, the mean lifespan of wild-type *C*. *elegans* propagated from L1 on OP50 was approximately 17 days. However, the deletion mutation in *ilys-3* decreased the mean lifespan by 24% to 13 days (*p* < 0.0001) (Fig 5D and 5E and S2 Table). The reduced life expectancy observed in *ilys-3* mutants was restored to that of wild-type in transgenic animals carrying transgenes *eEx752* or *eEx754*. Moreover, both transgenes extended average survival of mutant animals growing on OP50 by 20% (*p* < 0.0001), probably as a consequence of ILYS-3 overexpression.

Despite their identical average lifespans on pathogen lawns of CBX102 (10 days) N2 animals overexpressing ILYS-3 with *eEx754* also exhibited increased adult longevity relative to control animals without this transgene. Compared to N2 the average lifespan of *ilys-3* mutants on pathogen lawns decreased by 30% (from 10 days to 7 days, *p* < 0.0001), (Fig 5G and 5H and S3 Table). Interestingly, supernumerary copies of *ilys-3* seemed to have a protective effect mainly during adulthood. Animals carrying transgenes *eEx752* and *eEx754* showed statistically significant survival curves relative to WT in both *ilys-3* and WT backgrounds (S3 Table).

The translational fusion ILYS-3 protein appears to be fully functional and reverses the shortened lifespan of *ilys-3(ok3222)* when mutants were fed either OP50 or CBX102 diets.

To address the possibility that the reduced lifespan of *ilys-3* mutants was a consequence of subtle developmental defects and/or to a reduced fitness of animals, we measured the developmental rate of WT and mutants on live bacterial cells of OP50 and on the pathogen CBX102 (S8 Fig). Synchronized animals were added to each of the diets as L1 larvae and their developmental age was assessed 48 hours later. No significant differences were found between wild-type and mutants fed on live OP50 or CBX102 (*p* = 0.9 and *p* = 0.7, respectively, 99% confidence level). On pathogen lawns both WT and mutants exhibited similar developmental progression rates at 48 hours and the majority of the animals were of the same developmental age, at the L2 stage.

We therefore concluded that the pathogen-response differences between WT and mutants that we observed were acquired after larval development rather then early in life. Thus, infection by *M*. *nematophilum* might therefore pose a higher risk in mature and elder animals when immunity wanes.

Altogether these results show that ILYS-3 has an important role in the pharyngeal grinder for proper lysis of bacterial cells. The reduced enzymatic activity by this tissue results in increased bacterial colonization in the gut, and reduced lifespan.

### Abrogated coelomocyte activity does not compromise functional immune response, but exerts a small effect when combined with the loss of *ilys-3*

Although the major tissues that express ILYS-3 are associated with the digestive tract, we also detected expression in the coelomocytes. These are scavenger cells in *C*.*elegans* and have been shown to perform unspecific endocytosis of fluid-phase markers and macromolecules from the body cavity [48]. Although similar cells are believed to have an immune function in many invertebrates, in *C*. *elegans* their specific involvement in phagocytosis of whole bacteria seems unlikely and this arm of innate defense is apparently missing. Nevertheless, how did ILYS-3 appear in these cells? One possible scenario would be that ILYS-3::mCherry is secreted by intestinal and pharyngeal tissues and eventually taken up and degraded by the coelomocytes. Proteins with a secretion signal, such as ILYS-3 are translated and processed in the ER and might be secreted into the pseudocoelom before being endocytosed by coelomocytes. However, several lines of evidence indicate that coelomocytes directly produce ILYS-3. First, we observed coelomocyte expression in transgenic animals expressing the long promoter version of the GFP transcriptional reporter, thus suggesting that they are among the primary sites of *ilys-3* transcription activity (Fig 1K). Second, we found that absence of functional coelomocytes did not result in an increased level of ILYS-3:mCherry in the body cavity; for this we used the coelomocyte-deficient strain, NP717 (kind gift of H. Fares). NP717 animals have genetically ablated coelomocytes due to expression of a variant of the Diphtheria toxin A fragment (E148D) driven by the coelomocyte-specific *unc-122* promoter. These animals also express an ssGFP that is secreted by the body wall muscles and accumulates in the body cavity, demonstrating lack of coelomocytes and scavenging [48]. No accumulation of ILYS-3::mCherry in the pseudocoelom was detected in CB7137 animals (NP717 derivative strain) with ablated coelomocytes relative to their respective control counterparts. Finally, we found that in a *rme-1* deletion mutant which has impaired coelomocyte up-take of endocytosis markers, the population of ILYS-3::mCherry positive vesicles was unaffected (S9C Fig). These mutants exhibit an accumulation of the soluble GFP secreted from body-wall muscle cells in their pseudocoelom [42]. However, examination of ILYS-3 expression in this genetic background revealed that compromising endocytic function did not affect the fluorescence intensity, the size or the number of the coelomocytic vesicles. These findings indicate that coelomocytes directly produce ILYS-3.

In the coelomocytes, ILYS-3 is present in presumptive endosomes and lysosomes. This was observed by injecting Alexa-488 BSA into the body cavity of animals expressing ILYS-3::mCherry (S9A Fig). An hour after injection, a population of endocytosed Alexa-488-BSA bearing vesicles co-localized with mCherry positive compartments. These were subsequently confirmed as late endosome/lysosomes vesicles by doubly labeling ILYS-3::mCherry transgenic animals with the endocytic compartment marker GFP::RAB-7, which detects early/late endosomes and lysosomes (S9D–S9F Fig). We also found that in the *cup-5* mucolipin-1 mutants *(ar465)* the population of large vacuoles that correspond to late endosome-lysosome hybrids, were positive for ILYS-3::mCherry (S9B Fig). In these mutants, although lysosomes are able to fuse with late endosomes, the autolysosomes fail to degrade endocytosed material, thus resulting in accumulation of large vacuoles [48,49].

Given the ILYS-3 coelomocyte expression pattern, we reasoned that perhaps these cells might confer some protection against microbial pathogenesis, similar to their mammalian counterparts. To test this hypothesis we examined the effect of removing these cells on the lifespan of animals exposed to *M*. *nematophilum* and compared their response to that of wild-type worms. Depleting coelomocytes resulted in a wild-type response to the pathogen and the median lifespans of coelomocyte-ablated NP717 and N2 were indistinguishable (approximately 7 days) (S10 Fig). In contrast, in the coelomocyte-minus *ilys-3* double mutants (CB7137, an NP717 derivative strain) an enhanced susceptibility to the bacterial pathogen was observed relative to NP717 and to N2. The overall median lifespan of both NP717 and CB7137 animals decreased from 7 to 5 days, (p < 0.0001), and *ilys-3* double mutants were no different from *ilys-3* single mutants. However, a greater proportion of double mutant larvae succumbed to the pathogen at early time points, while older animals that managed to escape the initial bacterial burden exhibited similar mortality risk rates to those of the *ilys-3* single mutants (S10 Fig). When analyzed the two early survival curves were statistically different (*p* = 0.004). We concluded that removing coelomocytes *per se* did not shorten survivorship of wild-type worms and therefore these cells do not seem to provide a protective response to the pathogen *M*. *nematophilum*. However, when combined with a defective *ilys-3* function, lower survival rates in the younger cohorts were observed. Combined absence of functional *ilys-3* and coelomocytes contribute to disease pathogenesis, presumably due to a compound effect of gut luminal colonization and failure to remove/recycle unwanted macromolecules in younger animals. It is therefore likely that these cells play a more important role in hatchlings then in their older siblings and specially in situations where peptidoglycan overload occur.

### Pathogen-induction of intestinal *ilys-3* requires ERK signaling

The activation of the *e*xtracellular signal-*r*egulated *k*inase (ERK) *m*itogen-*a*ctivated *p*rotein (MAP) kinase cascade is required to mount a protective response against *M*. *nematophilum* and consequently loss-of-function mutants of MPK-1, the *C*. *elegans* ortholog of the mammalian ERK1/2, are more susceptible to this bacterial pathogen [50,51].

**Fig 6:**
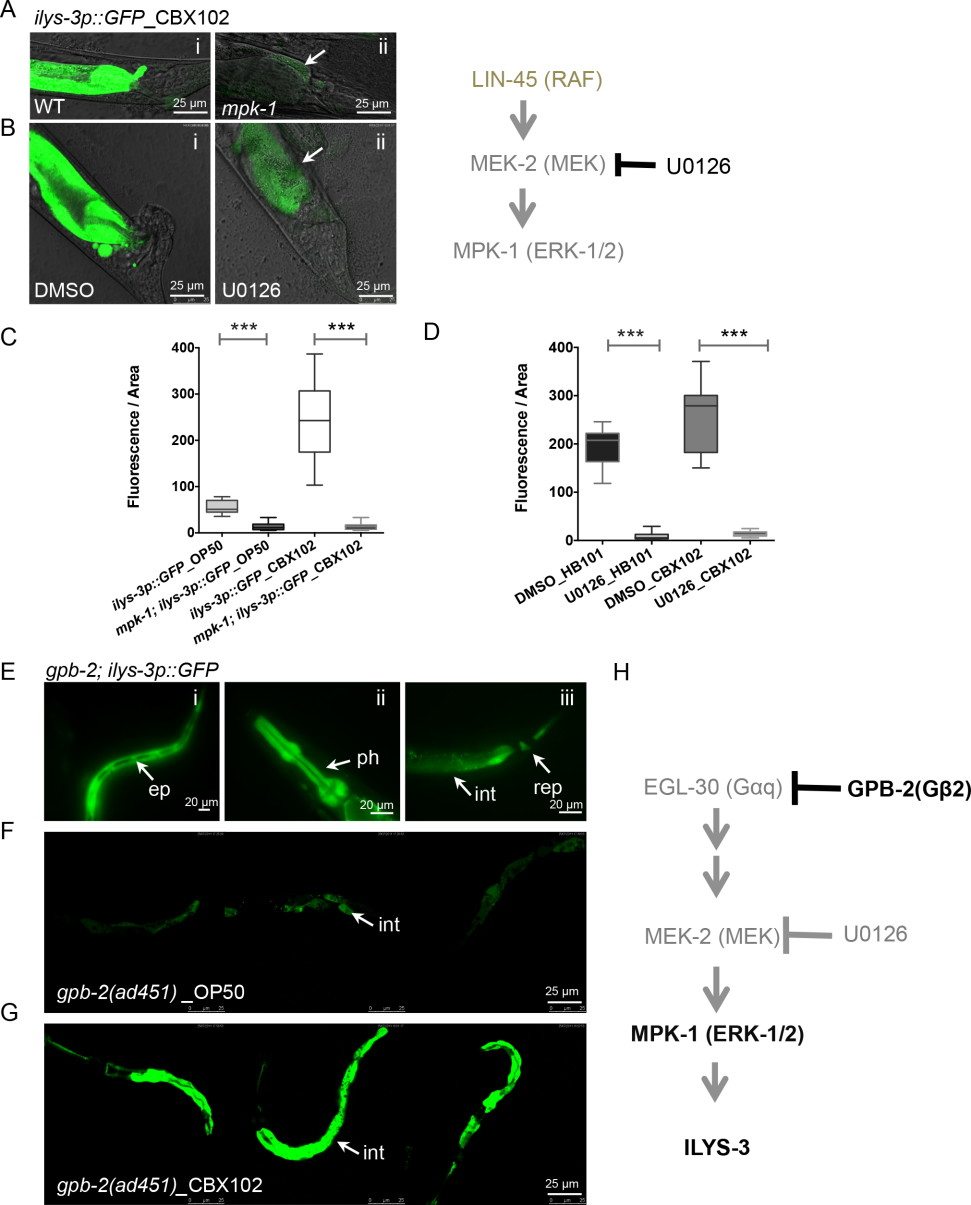
Induction of *ilys-3* requires ERK. (A) *mpk-1* mutants show reduced or no *ilys-3* transcription in the intestine and cannot induce *ilys-3* when exposed to *M*. *nematophilum* CBX102 (arrow). Images show representative fluorescence of the intestine of adults carrying the *ilys-3p*::*GFP* transgene grown on CBX102 in the strains: (i) *ilys-3p*::*GFP* in the wild-type background and (ii) *mpk-1(ku1); ilys-3p*::*GFP*. (B) Post-developmental inhibition of the MEK activity using the chemical inhibitor U0126 that mimics *mpk-1(ku1)* loss-of function mutants. (i) Detail of animal exposed to DMSO (control) and (ii) U0126 treated animal showing reduced GFP signal in the gut after exposure to CBX102. Note the decreased fluorescence in the intestine of an animal treated with U0126 (arrow) (ii) and the reduced swelling in contrast to the DMSO-treated control (i). (C) Expression of *ilys-3p*::*GFP*, measured by fluorescence intensity in the intestinal cell int8 in WT and mutant backgrounds, expressed in arbitrary fluorescence units. ROI was set to 40 μ diameter and 0.4 μ thickness. Asterisks indicate the results of a Mann–Whitney Unpaired test statistical comparisons of the fluorescence intensity for *mpk-1(ku1); ilys-3p*::*GFP*_OP50 *vs ilys-3p*::*GFP*_OP50 and *mpk-1(ku1); ilys-3p*::*GFP*_CBX102 *vs ilys-3p*::*GFP*_CBX102 (*** p < 0.0001). Mean values for *mpk-1* mutants on CBX102 were not significantly different from their sibling controls on OP50 (*p* = 0.6098). N = 15 per group. (D) Confocal quantification of the mean fluorescence intensity in the intestinal cell int8 of L4 expressing *ilys-3p*::*GFP* treated with 50 µM of the MEK inhibitor U0126 or DMSO as control. ROI was set to 40 µ diameter and 0.4 µ thickness. Asterisks indicate the results of a Mann–Whitney Unpaired test statistical comparisons of the fluorescence intensity for worms treated with U0126 relative to the DMSO control. Mean values of U0126 on HB101 and on CBX102 all differed significantly from their respective controls (*** *p* < 0.0001). The mean values for DMSO treated worms on CBX102 were marginally different from those for OP50 controls (*p* = 0.022). 95% confidence interval. N = 15 per group. (E) Activation of ERK MAPK kinase in the *gpb-2* mutant leads to overexpression and ectopic expression of *ilys-3* in (i) epidermis (ep) but not seam cells, (ii) whole pharynx (ph) (iii) intestine (int) and rectal epithelium (rep). (F) Representative images of *gpb-2* mutants on OP50. (G) Induction of *ilys-3* in the gut of *gpb-2* mutants after 24 hours on 100% CBX102. Gain was set to the brightest samples i.e. *gpb-2* in CBX102. (H) Representation of the ERK-1/2 MAPK genetic pathway that is activated by EGL-30 (Gαq) in the context of the morphological changes that take place for the rectal epithelial cell swelling in response to *M*. *nematophilum*. Presumably in this pathway, GPB-2 (Gα2) negatively regulates EGL-30 (Gαq), which in turn, is required for activation of MEK-2 and its downstream effector MPK-1. The network that regulates the transcriptional induction of ILYS-3 upon *M*. *nematophilum* infection (this work) is under the control of MPK-1 and the negative regulation by GPB-2. This signaling cascade can be blocked by the MEK inhibitor U0126, corresponding to *mpk-1* loss-of-function.

We therefore tested whether the ERK pathway could account for the up-regulation of *ilys-3* after *M*. *nematophilum* infection. We found that induction of *ilys-3* in the intestine of *mpk-1(ku1)* mutants exposed to *M*. *nematophilum* was severely compromised (Fig 6Ai and 6Aii). This was confirmed by measuring the relative fluorescence intensity of *ilys-3p*::*GFP* transcriptional reporter in the intestinal cell int8 of WT and mutant animals (Fig 6C), respectively. Inactivation of *mpk-1* resulted in a significant reduction or absence of the *ilys-3* transcriptional activity in the gut of animals grown on OP50 as well as on CBX102 (Fig 6Ai and 6Aii). In contrast, pharyngeal *ilys-3* remained largely unchanged (as shown below).

These results were confirmed by assaying the effect of the MEK inhibitor U0126 on the expression of *ilys-3* reporter in the gut of L4 WT animals grown on HB101 or on 10% CBX102 (Fig 6Bi and 6Bii). Exposure of the worms to the MEK inhibitor resulted in a statistically significant decrease of GFP in the intestinal cell int8, when compared to DMSO controls. This effect was seen when worms were grown on *E*. *coli* or challenged with CBX102 (Fig 6D). We also confirmed the effect of inhibition of ERK signaling pathway on the reduction of intestinal *ilys-3* expression, by feeding L4 animals with *mpk-1* dsRNA. Consistent with our mutant analysis, knockdown of this gene also diminished the GFP transcriptional reporter intensity in the intestine but not in the pharynx (S11 Fig). We concluded that intestinal induction of *ilys-3* triggered by *M*. *nematophilum* infection is dependent on MPK-1 activity.

ERK activation has also been shown to mediate the detrimental effect of starvation in pharyngeal muscle of *C*. *elegans*, via the signaling transduction pathway muscarinic acetylcholine receptor-Gqα-PKC-MAPK [52]. We hypothesized that if activation of *ilys-3* was also due to a response to starvation, then the loss-of-function *gpb-2* mutants that are hypersensitive to muscarinic signaling and starvation should show increased *ilys-3* activity in the gut. We found that this was the case. *gpb-*2 is the ortholog of vertebrate G β5 and encodes the β subunit of a heterotrimeric G protein that binds the Gqα EGL-30, functioning to negatively regulate signaling in the pharyngeal muscle. In these mutants *ilys-3* was overexpressed (Fig 6E). GFP was abundant not only in the pharynx (Fig 6Eii), intestine and rectal epithelium (Fig 6Eiii) but also in other tissues such as epidermis (Fig 6Ei). In *gpb-2* mutants the muscarinic acetylcholine signal cannot be down-regulated and starvation has detrimental effects in the pharynx [52,53].

We next asked whether hyperactivation of the pharyngeal MAPK pathway could affect the worm responses to CBX102. In assays with 100% lawns of CBX102, *gpb-2(ad541)* L1 larvae die in 3 days whereas WT had an average lifespan of 10 days. This early death is likely due to unrestrained pharyngeal damage [52], despite the fact that in this mutant background expression of an *ilys-3* reporter was markedly induced following *M*. *nematophilum* infection (Fig 6F and 6G). We estimated a two-fold increase of the *ilys-3p*::*GFP* transcriptional reporter in the intestinal cells of transgenic mutant animals, relative to uninfected siblings (S12 Fig).

Overall, our results revealed a transcriptional program leading to ILYS-3 induction upon *M*. *nematophilum* infection, which is mediated at least in part by the ERK-MAPK and the negative regulator GPB-2 (Fig 5H). Loss-of-function in GPB-2 causes MAPK hyperactivation, damage to the pharynx with probably enhanced susceptibility to both starvation and pathogen despite increased ILYS-3 expression. Our results also suggest that *ilys-3* is a useful *in vivo* sensor for monitoring responses to starvation as well as to pathogens.

### The pathogen-induced activation of intestinal ILYS-3 is cell-non-autonomous and requires MPK-1 activity in the pharynx

**Fig 7:**
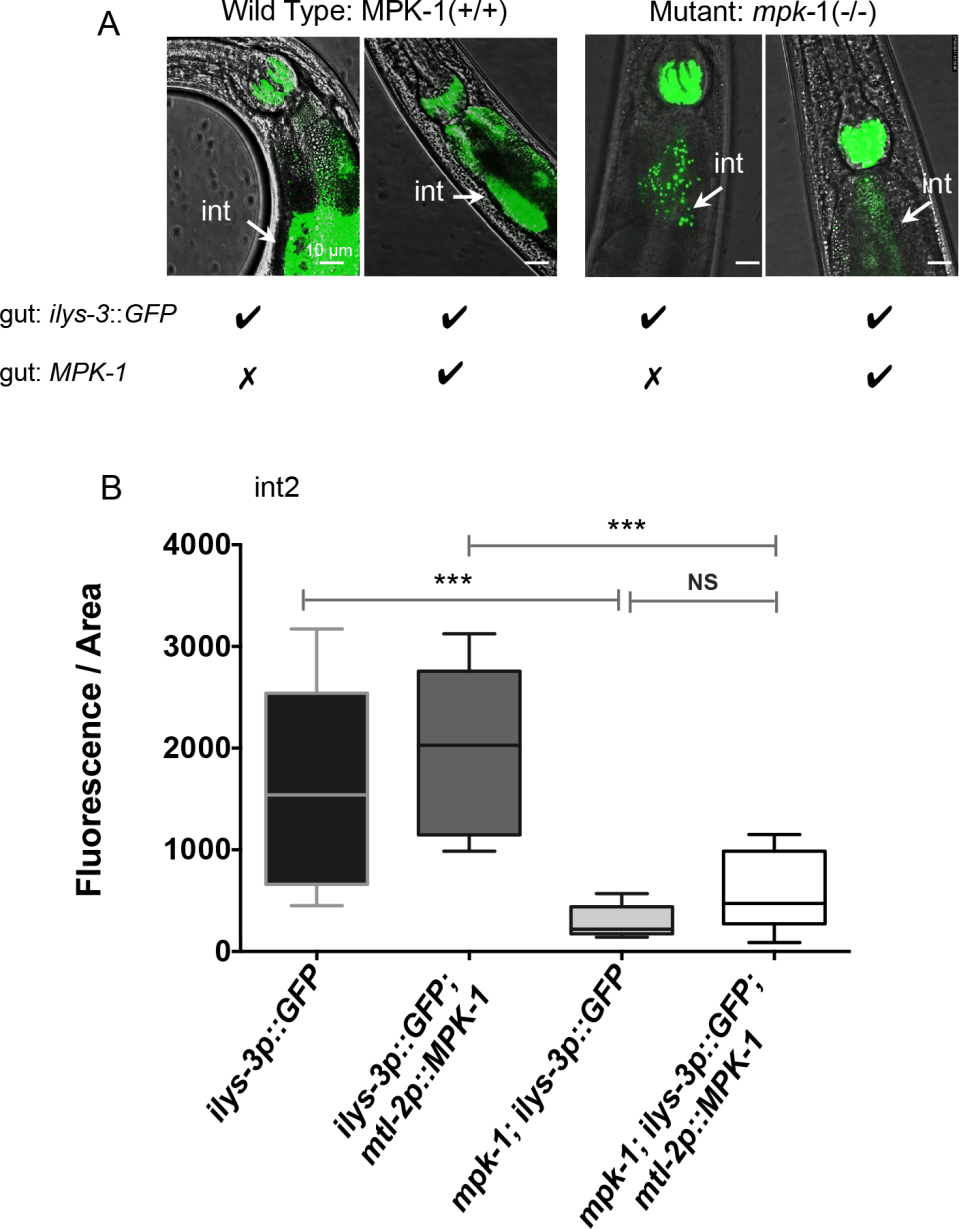
The activation of ILYS-3 does not require MPK-1 activity in the gut. (A) Images of single and double transgenic animals carrying the *ilys-3p*::*GFP* reporter without or with the transgene *mtl-2p*::*MPK-1* in the WT (N2) and in the *mpk-1(ku1)* backgrounds. The construct *mtl-2p*::*MPK-1* drives MPK-1 expression in the intestine (int). In the *mpk-1* mutants, *ilys-3* expression was blocked. This phenotype was not rescued when MPK-1 is restored in the intestine. (B) Quantification of fluorescence intensity in the intestinal cell int2. Asterisks indicate the results of a Mann–Whitney Unpaired test statistical comparisons of the fluorescence intensity for *mpk-1(ku1); ilys-3p*::*GFP; mtl-2p*::*MPK-1vs ilys-3p*::*GFP; mtl-2p*::*MPK-1*(*** *p* = 0.0005) and *mpk-1(ku1); ilys-3p*::*GFP vs ilys-3p*::*GFP* (*** *p* = 0.0002). Mean values for *mpk-1* mutants with the double transgene were not significantly different (NS) from their sibling controls harbouring the *ilys-3p*::*GFP* reporter only (*p* = 0.0934). N = 10-15/group.

The *C*. *elegans mpk-1* gene is expressed in the nervous system and in many tissues including the pharyngeal-intestinal valve, intestine, body wall muscles and rectal epithelium ([54] and https://www.wormbase.org). For this reason we asked whether intestinal expression of *mpk-1* alone could modulate the induction of the intestinal *ilys-3* in the mutant background in response to the pathogen, thus working in a cell autonomous manner. Surprisingly, we found that this was not the case. Overexpression of MPK-1 in intestine under the intestine specific promoter *mtl-2* (*mtl-2p*::*MPK-1*) did not rescue GFP reporter levels in the gut of *mpk-1(ku1)* mutant animals (Fig 7A). The levels of fluorescence in the intestinal cells int2 and int8 of single and double transgenes remained largely unaltered (Fig 7B and S13 Fig, respectively).

We also tested whether increased expression of the MAP kinase cascade in the rectal epithelium could lead to increased *ilys-3p*::*GFP* signal in the intestine in view of the known importance of ERK signaling in the rectal cells. For this, we used a strain that bears a rectal enhancer element of *egl-5* coupled with the *pes-10* minimal promoter driving expression of LIN-45(S312A,S453A) (Raf) (RE::LIN-45*), [55]. Although constitutive activation of the swelling response was seen in transgenic animals, as expected, no increase in levels of *ilys-3p*::*GFP* were observed in the gut (S14 Fig).

**Fig 8:**
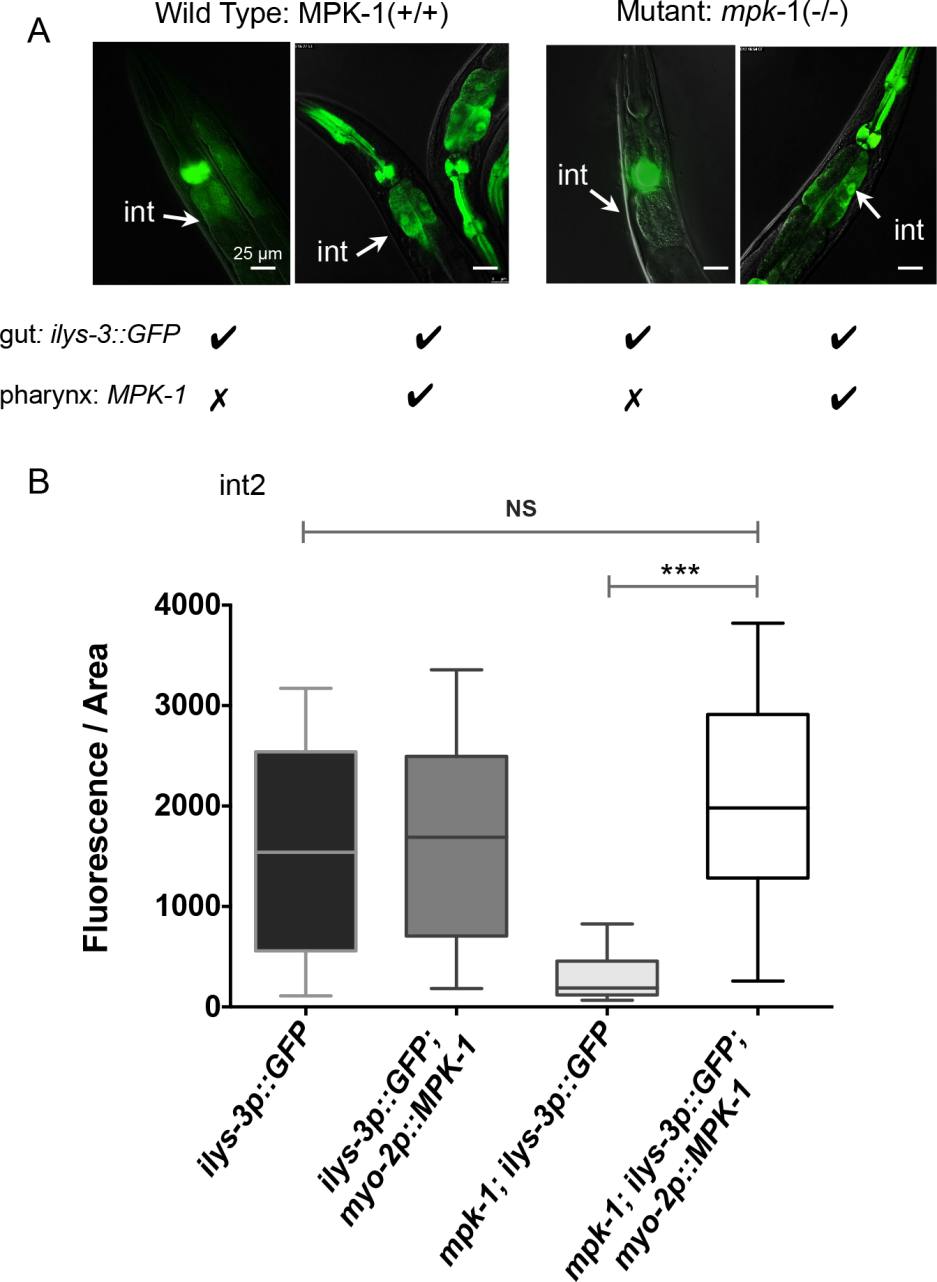
The activation of ILYS-3 is cell non-autonomous and requires MPK-1 activity in the pharynx. (A) Images of single and double transgenic animals expressing *ilys-3p*::*GFP* only or in combination with the *myo-2p*::*MPK-1* in *mpk-1(ku1)* and WT (N2) backgrounds. The *myo-2p*::*MPK-1* construct drives MPK-1 expression in the pharynx and restored *ilys-3* expression in the intestine (int) of *mpk-1* mutants. (B) Quantification of fluorescence intensity (after background subtraction) in the intestinal cell int2 of single and double transgene reporter strains. Data analyzed with Mann–Whitney Unpaired test, 95% confidence level. Fluorescence intensity values for *mpk-1(ku1); ilys-3p*::*GFP*; *myo-2p*::*MPK-1 vs ilys-3p*::*GFP; myo-2p*::*MPK-1* and *mpk-1(ku1); ilys-3p*::*GFP; myo-2p*::*MPK-1 vs ilys-3p*::*GFP* were not significantly different (NS). Mean values for *mpk-1* mutants with the double transgene differ significantly from their sibling controls harbouring the *ilys-3p*::*GFP* reporter only (*** *p* = 0.0003). N = 10-15/group.

We next tested whether restoring MPK-1 specifically in the pharynx of *mpk-1(ku1)* mutants had an effect on intestinal induction of *ilys-3p*::*GFP*. We used a strain that expressed the wild-type *mpk-1* from the pharyngeal specific promoter *myo-2*, a myosin heavy chain isoform gene expressed specifically in pharyngeal muscles (kind gift of L. Avery). We measured the fluorescence of intestinal *ilys-3* in int2 and int8 cells in *mpk-1* and wild-type single and double transgenic animals and compared to levels of expression in their single transgenic siblings (Fig 8 and S15 Fig). Pharyngeal expression of MPK-1 completely recapitulated the intestinal expression of *ilys-3* pattern generated in the wild-type reporter strain (Fig 8A). Statistically significant high levels of intestinal GFP were detected in *mpk-1* double transgenic animals compared to their single transgenic sibling mutants (Fig 8B). Furthermore, and as shown in Fig 8B and S15 Fig, no significant differences were found in the average levels of intestinal *ilys-3* fluorescence between mutant and wild-type double transgenes. Thus, presence of MPK-1 in the pharynx resulted in an increased expression of *ilys-3p*::*GFP* and also rescued reporter gene induction in the intestine of the *mpk-1(ku1)* mutant. To corroborate the results described above, we used quantitative real-time PCR analysis to determine *ilys-3* transcriptional activity in whole organism in *mpk-1* mutants harboring the tissue specific transgenes that rescue MPK-1 activity in the pharynx and in the intestine (S16 Fig and S4 Table). Consistent with our confocal microscopy analysis we were able to observe high *ilys-3* mRNA induction levels in the *mpk-1* mutant only when the pharyngeal but not the intestinal MPK-1 transgene was present. Particularly high levels of *ilys-3* basal transcription activity were detected in animals harboring the pharyngeal MPK-1 cDNA transgene reared on OP50 lawns. This is also in agreement with the microscopic analysis using the *ilys-3* GFP reporter.

Overall, these results indicate that for its response to infection by *M*. *nematophilum*, intestinal ILYS-3 induction depends on MPK-1 activity in the pharynx. High levels of MPK-1 activity in the pharynx of *mpk-1* mutant resulted in generally detrimental effects in most animals, making it impossible to complete pathogenicity assays, as the transgenic line was difficult to maintain. We therefore could not directly test whether activation of pharyngeal MPK-1 was protective against bacterial infections. This deleterious effect is consistent with previous reports [52].

Altogether these results showed that intestinal MPK-1 activity is not required for *ilys-3* pathogenic induction in this tissue, whereas pharyngeal MPK-1 activity is.

However, under nutrient depletion, and in contrast to the bacterial-mediated response, inactivation of ERK-MAPK by mutation did not block induction of the *ilys-3* reporter in the intestine (S17 Fig); thus indicating that immunity and nutrient depletion can be uncoupled and are presumably, under the control of two distinct regulatory networks.

### Recombinant ILYS-3 exhibits hydrolytic activity on Gram-positive bacterial cell walls

**Fig 9:**
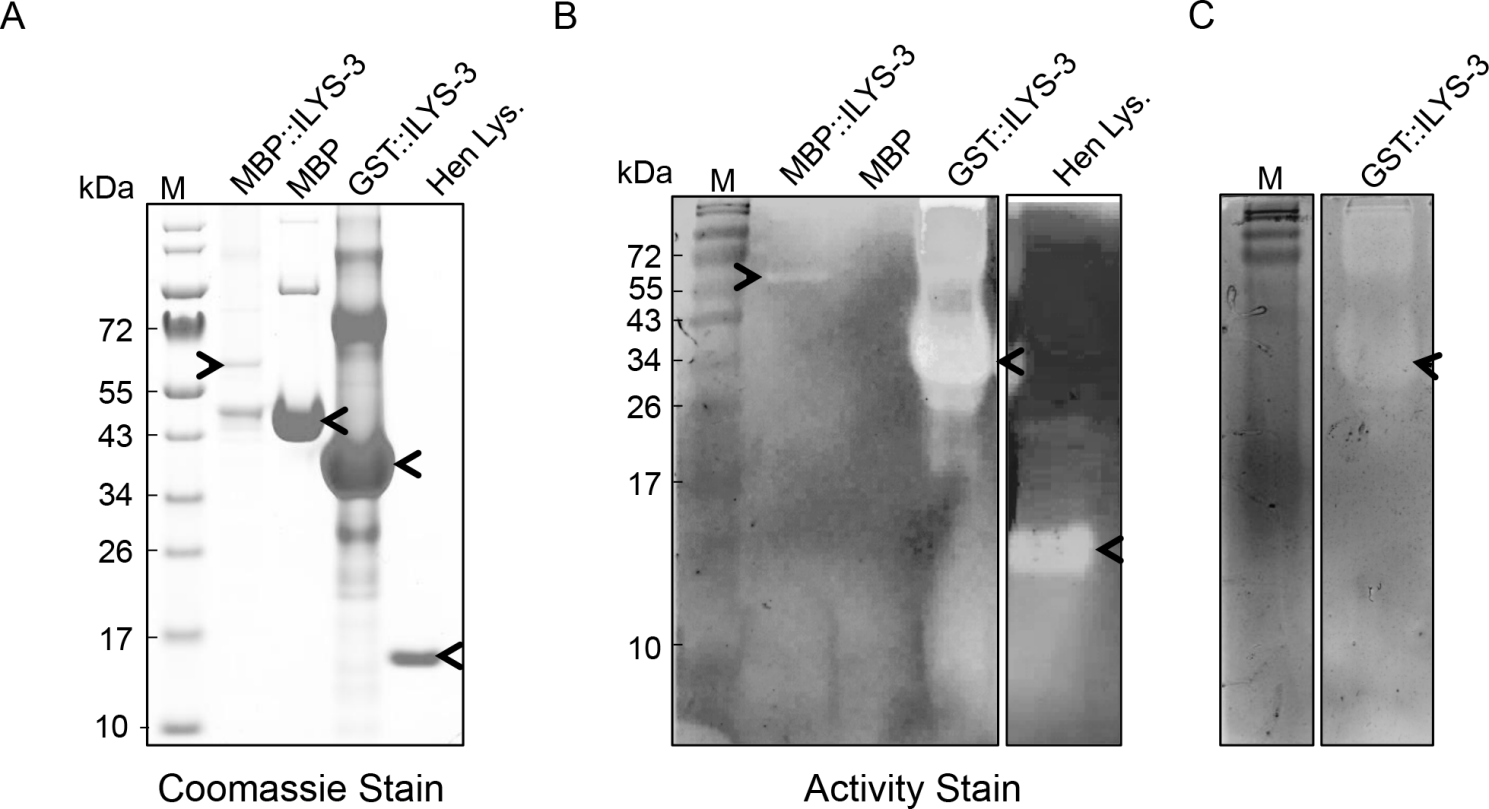
Recombinant ILYS-3 possesses hydrolytic activity on *M*. *luteus* and *M*. *nematophilum*. (A) SDS-PAGE analysis of the purified rILYS-3 fusion proteins under non-reducing conditions. Lanes M: protein marker; MBP::ILYS-3: Purified MBP::ILYS-3 fusion protein (4 µg); MBP: purified MBP migrates as 45 kDa (10 µg); GST::ILYS-3: solubilized inclusion bodies harboring GST::ILYS-3 signal less fusion protein; Hen LYS.: Hen egg-white lysozyme migrates as 14 kDa (1 µg). Gel was visualized by Coomassie staining. The target protein is indicated by arrowhead. (B) Zymogram analysis of recombinant ILYS-3 fused to MBP or GST on an SDS-polyacrylamide gel with *M*. *luteus* cells. The hydrolytic activity was assayed by methylene blue staining. Samples appear in the same order as in (A). Non-stained zones indicate peptidoglycan degradation. The recombinant MBP::ILYS-3 fusion protein produced a band of clearing at the expected position (arrowhead), indicating peptidoglycan-hydrolytic activity. Purified Hen LYS and MBP alone were used as positive and negative controls, respectively. No gel clearing was detected with MBP alone. Solubilized inclusion bodies recovered from IPTG-induced *E*. *coli* BL21 harboring the recombinant GST::ILYS-3 signal peptide less construct produced a clear band resolved at 41 kDa (expected size), and denotes enzymatic activity. The target protein is indicated by arrowhead. (C) Zymogram analysis of recombinant ILYS-3 signal less peptide fused to GST on an SDS-polyacrylamide gel with *M*. *nematophilum* cells. The target protein is indicated by arrowhead.

In view of the evident importance of this invertebrate lysozyme, both in healthy and diseased worms we next wanted to analyze the enzymatic properties of ILYS-3, and prepared a purified recombinant protein. A construct was designed that allowed expression of the full length of ILYS-3 (139 amino acids) fused with *E*. *coli* maltose-binding protein (MBP, 367 amino acids) via a 47 amino acid linker. The purity and approximate molecular mass of (r)ILYS-3 were assessed by performing SDS–PAGE on a 10–12% gradient gel (Fig 9). The size of the fusion protein was approximately 58 kDa (including MBP-tag; 42.5 kDa), which was consistent with the predicted size of the fusion protein. Expression of rILYS-3 was accompanied by proteolytic activity resulting in an additional band of 43kDa in size that corresponded to MBP protein fused with the first 5 amino acids of ILYS-3 (Fig 9A).

Next rILYS-3 was tested *in vitro* for its ability to cleave peptidoglycan in cell walls of the standard bacterial strain, *M*. *luteus*. For this, we employed a zymogram assay, an electrophoretic method that is based on an SDS polyacrylamide gel impregnated with bacterial cells as substrate, which becomes hydrolyzed by the protein during the renaturation period. Methylene blue staining of the gel reveals sites of hydrolysis as white bands on an otherwise dark blue background [56]. Hen egg-white lysozyme (Hen Lys) served as a positive control in this assay. As shown in Fig 9B both Hen Lys and rILYs-3 display cell-wall degrading lytic activity, suggesting that ILYS-3 is capable of releasing peptidoglycan fragments in the same way as Hen Lys. A recombinant ILYS-3 fused to GST at the N-terminus, was also made and IPTG-induced bacterial cell extracts were found active on dead cells from both *M*. *luteus* (Fig 9B) and *M*. *nematophilum* (Fig 9C). In contrast, Hen Lys was only active on *M*. *luteus*.

The pH of the *C*. *elegans* lumen ranges from 5.96 ± 0.31 in the anterior pharynx to 3.59 ± 0.09 in the posterior intestine [57]. We investigated whether ILYS-3 is active under these conditions. For this we performed zymogram analysis with proteins renatured on gels exposed to buffers at different pH values. We found it maximally active between pH4.5 and 5.0, although a pronounced activity of the rILYS-3 was also seen at pH3.0. This coincides with the luminal pH of *C*. *elegans* intestine and the highly acidic digestive organelles lysosomes. The zymograms shown in Fig 9B and 9C depict rILYS-3 activity at pH5.0.

The hydrolytic activity by ILYS-3 is in agreement with its high sequence homology to the bivalve invertebrate lysozymes in *Tapes japonica* (PDB No 2DQA) and *Meretrix lusoria* (PDB No. 3AB6), which have muramidase activity [Percentage of pairwise sequence identity with ILYS-3 were of 51%, and 49% with 7.00 × 10^−24^ and 6.00 × 10^−23^ blast expectation values, respectively) [(https://www.predictprotein.org/)] (S18 Fig). The tertiary structural model of ILYS-3 shown in S18A Fig was constructed by performing homology-based modeling using the iTasser server. The model matches the crystal structure of the NAG-bound lysozyme from *Meretrix lusoria* (Chain A; PDB No. 3ab6) that has six α-helices and two β-sheets.

We concluded that the *C*. *elegans* ILYS-3 appears to be largely similar to the invertebrate lysozymes from bivalves [29,58]. Given that an intact cell wall is essential for bacterial viability and that the invertebrate lysozyme ILYS-3 has an *in vitro* lytic activity, we propose that ILYS-3 is likely to be an effector that affects this cell integrity thus reducing bacterial proliferation in the nematode and aiding in the digestion of shed PGN.

Attempts to demonstrate direct bactericidal action of recombinant ILYS-3 on live *M*. *nematophilum* cells were not successful, probably due to protein aggregation and perhaps a need for additional digestive factors that are present in the natural situation.

## Discussion

Central to the capacity of intestinal epithelial cells to maintain immune barrier functions is an ability to produce inducible antimicrobial effectors. Our results show that the intestinal cells of *C*. *elegans* are capable of producing and inducing a candidate bactericidal effector, ILYS-3 that helps prevent Gram-positive bacterial pathogen colonization, by cleaving and perhaps recycling the load of peptidoglycan fragments which might otherwise overwhelm the whole epithelia. Moreover, we show that pharyngeal ILYS-3 has an important role for lysing both Gram-positive and Gram-negative bacteria.

In multicellular organisms danger inflicted on cells results in alarm signals that activate innate immune responses. Thus, a host mechanism that propagates danger signals to neighboring cells and tissues is advantageous for pathogen clearance and damage repair. In the present study, we show that during *M*. *nematophilum* infection, the activation of the intracellular ERK-MAPK signaling cascade in the pharynx results in a signal that leads to *ilys-3* transcription in a distal tissue, the intestine. This communication between pharyngeal muscle and intestinal epithelium enables host cells to increase the expression of effector ILYS-3, at a site where bacterial colonization occurs.

*M*. *nematophilum* infection represents a suitable model system to study chronic colonization of the intestinal epithelium in *C*. *elegans*. This is because in contrast to what is seen with bacterial pathogens that rapidly kill the nematode, *M*. *nematophilum* affects the worm fitness and elicits immune reactions as a consequence of its persistent infection in the intestine. Faced with continuous pathogen stimulation, the intestinal epithelium of infected worms thus requires a balance between mounting an exacerbated response or maintaining steady-state levels of antimicrobial expression.

Here we have elucidated the biological function of the *C*.*elegans ilys-3*, a member of the invertebrate type of lysozymes. We have found that one of the biological roles of *ilys-3* is to aid bacterial grinding and lysis, which are required for nutrient digestion. Firstly, *ilys-3* is expressed primarily in digestive tissues of *C*. *elegans*: the pharyngeal grinder and the intestine. Furthermore, upon ingestion of non- or mildly-pathogenic bacteria, animals with depleted levels of ILYS-3 showed increased intestinal bacterial cell counts, indicative of enhanced bacterial proliferation. This intestinal bacterial overgrowth is likely to be associated with the acquisition of the aging-related phenotype observed in *ilys-3* mutants which exhibited reduced lifespan on both OP50 and CBX102. This is in line with findings in *Drosophila* [59] and *C*. *elegans* [46,60]. In these two models of aging, bacterial overgrowth acts on the organismal lifespan. Manipulations on the microbial dynamics with antibiotics were seen not only to prevent bacterial cell proliferation within their hosts but also to increase fly and worm lifespans.

Several lines of evidence indicate that ILYS-3 works as immune effector in *C*. *elegans*. First, we provide biochemical evidence that ILYS-3 has *in vitro* hydrolytic activity for the Gram-positive bacterial cell walls of *M*. *luteus* and *M*. *nematophilum*. Peptidoglycan consists of glycan strands of alternating β-1,4-linked *N*-acetylglucosamine (Glc*N*Ac) and *N*-acetylmuramic acid (Mur*N*Ac) residues cross-linked to each other by short peptides made of L- and D-amino acids. *C*. *elegans* ILYS-3 has the conserved Aspartate and Glutamate residues responsible for muramidase activity and is probably capable of generating PGN fragments by hydrolyzing the β(1,4) linkages between *N*-acetylmuramic acid and *N*-acetylglucosamine residues in PGN. However, we have not established the exact sites of ILYS-3 cleavage in the complex peptidoglycan polymer. Likewise, we have not demonstrated that purified ILYS-3 can act alone as bactericidal protein. Animal lysozymes have been implicated in peptidoglycan hydrolysis and innate immunity, via the generation of soluble and immunogenic PGN fragments, which themselves act as ligands and can be detected by the host immune system [61,62]. In mice, the cutaneous innate defense response and subsequent NOD2 detection of the extracellular pathogen *S*. *aureus*, requires the intracellular delivery of peptidoglycan-derived muramyl dipeptides through the insertion of the pore-forming toxin α-hemolysin into the host membranes [63]. The second domain of catalytic activity predicted in invertebrate lysozymes is isopeptidase. However, our homology searches revealed that of the six invertebrate-type lysozymes present in the *C*. *elegans* genome, only *ilys-4* has intact isopeptidase putative residues (S95 and H123). All the others appeared to have lost the Serine residue, which has been substituted by Alanine. Therefore they are expected to lack isopeptidase activity.

Second, the prolonged induction of *ilys-3* suggests that sustained high levels of this antimicrobial are necessary to provide protection against a persistent pathogen such as *M*. *nematophilum*. This suggests that ILYS-3 is a slow and long-lasting effector, which takes longer to attain maximal levels and strengthen its antimicrobial function. Such sustained antimicrobial induction could be designed to entail an efficient protection against more persistent pathogens that resist the first line of effectors and remain resident within the host tissues. As mentioned above, ILYS-3 can also be induced by the Gram-positive *S*. *aureus*, which is capable of activating a strong transcriptional response in *C*. *elegans* intestinal epithelial cells. Work by Irazoqui showed that *ilys-3* is highly responsive to this pathogen and its transcriptional activity started early in time by 4 hours, but continued to increase by their final assayed time point (12 hours) [8].

Intestinal function is reinforced by the luminal secretion of antimicrobials which disrupt essential features of bacterial biology and creates the first line of immune defense. We wished to address the localization of ILYS-3 and to identify the machinery responsible for its transport in the polarized intestinal epithelia and secretion to the gut lumen. Surprisingly, and despite the many candidates for secretion into the intestinal lumen, there is no published direct evidence for a secreted gut enzyme in *C*. *elegans*. We have been unable to ascertain the route of the abundant luminal ILYS-3::mCherry secretion detected in old adults and dauers, as our experiments using markers for apical secretion were inconclusive. Although apical levels of ILYS-3::mCherry were usually below thresholds of detection, we found that the fusion protein can be routed to the apical domains of the intestinal cells. This conclusion was based on experiments that reduced the function of basolateral recycling vesicles in the *rab-10* genetic background. We also observed that ILYS-3::mCherry can be recycled basolaterally in a *rme-1* independent manner which suggests that ILYS-3 may work partly within the recycling endosomes and acidic vesicles of the intestinal epithelium. Digestion of PGN fragments and bacterial fragments may require the coordinated action of lysozymes that work effectively both in apical and basolateral environments. In this scenario, ILYS-3 would help detoxifying and clearing the remained PGN fragments that managed to escape the initial action of lytic enzymes and end up intracellularly and/or in the body cavity. This is in keeping with the observations that ILYS-3 is expressed in the recycling endosomes and the acidic vesicles LROs of the intestine but also in the late endosomes and lysosomes in the coelomocytes.

The expression patterns we observed were obtained using protein expressed from multicopy arrays, and therefore potentially affected by overexpression artifacts. However, the transgenes used efficiently rescued all mutant phenotypes and did not exhibit obvious dominant effects. Moreover, the rapid and reversible changes in distribution in response to nutrient availability indicate that the reporter reliably reflect endogenous protein distribution.

Third, disruption of *ilys-3* renders mutant animals more susceptible to the bacterial pathogen *M*. *nematophilum*. Fourth, overexpression of ILYS-3 leads to an enhanced protection against *M*. *nematophilum* in both *ilys-3* mutants and wild-type backgrounds.

It is intriguing that ILYS-3 is detected in coelomocytes, the six scavenger cells present in the adult hermaphrodite that continuously and nonspecifically recycle and endocytose macromolecules from the pseudocoelom [48]. These cells are believed to perform detoxification, acting like hepatic cells in higher animals [64]. In addition, they have been shown to work as sensors for dietary restriction-mediated longevity in *C*. *elegans* [65]. Our results suggest that coelomocytes are ILYS-3-producing cells but we ruled out the possibility that they contribute in any major way to the defense response of the *C*. *elegans* wild-type to *M*. *nematophilum*. Nevertheless, once the combined functions of *ilys-3* and the coelomocytes were compromised, a small effect was observed. The effect could be due to the excessive tissue exposure to PGNs resulting from lack of active processing.

Actively growing Gram-positive bacteria release large amounts of peptidoglycan fragments as a consequence of cell remodeling during cell division. Work done in *Bacillus megaterium* and *B*. *subtilis* established that 30–50% of PGN can be shed into the growth medium in each bacterial generation during exponential growth [66]. The intestinal environment of *C*. *elegans* can not be reproduced in a liquid-based medium, and consequently bacterial growth may differ in these two situations. However, bacteria that reside and proliferate in the nematode intestine, like *M*. *nematophilum*, can be expected to shed PGN fragments as part of normal growth, which may represent a significant burden on the intestinal cell function.

We found that in the intestine *ilys-3* has a post-embryonic regulated expression pattern, suggesting that it might be important for maintaining nutrient levels or immune homeostasis during certain developmental transitions. In conventionally reared animals, high levels of *ilys-3* transcriptional activity were detected in the intestinal cells of L1 at hatching, but these declined as animals reached L2. In early adults however, levels of intestinal *ilys-3* increased and were sustained thereafter. The physiological relevance of such transient expression pattern where steady state levels of *ilys-3* are kept high in hatchlings and adults may reflect the distinct needs to provide protection against bacterial colonization during key post-developmental stages. In the early phase of post-embryonic life, the intestine of naïve L1 may need to be primed with effectors that ensure safe first contact with food, in adulthood, the hermaphrodite intestine may become more vulnerable as its resources become directed towards nurturing the germline with yolk proteins.

With the onset of larval diapause, dauers might also need to be primed with effectors and signaling molecules that help them withstand harsh conditions and/or prepare them to get ready for feeding. We showed that upon nutrient depletion, luminal secretion of ILYS-3::mCherry was both very dynamic and reversible in these larvae. The fusion protein was secreted and seen to accumulate in the intestinal lumen, only to return rapidly to its cytosolic default state when animals encountered food and resumed development. A similar changing pattern of secretory polarity has also been reported for INS-35 and INS-7 [67], two insulin-like peptides that suppress larval diapause. These peptides, are usually secreted into the pseudocoelom in larval stages during normal development, but during diapause they are routed to the intestinal lumen where they are degraded. However, we do not have any evidence for the luminal degradation of ILYS-3:mCherry. In fact, in 25-day-old larvae the signal of fusion protein did not decrease and only changed as larvae adapted to resume development upon feeding. Unlike in starvation-induced dauers, food deprivation during other larval stages did not provoke accumulation of ILYS-3 in the lumen. These changing patterns of ILYS-3 distribution reveal yet another facet of that adaptive changes that occur in dauer development and demonstrate remarkable plasticity in the intestinal cells.

In *C*. *elegans*, most intestinal immune effectors that protect against many pathogens appear to act locally and respond cell autonomously to the activation afforded by key regulators such as the p38 MAP kinase PMK-1 [68] [13,28,69,70], DAF-16 [71] and ZIP-2 [72]. Interestingly, in the *S*. *aureus* model of infection intestinal *ilys-3* induction requires the proline hydroxylase EGL-9 which under normal O_2_ levels, functions in a conserved hypoxia-sensing pathway to convert the hypoxia-inducible factor, HIF-1 [73].

In contrast, our results indicate that for the *M*. *nematophilum*-induced *ilys-3* transcription an ERK/MAPK activity is required just as the starvation-mediated response in the pharyngeal muscle of *C*. *elegans* does [52]. Intense induction of *ilys-3* was found in *gpb-2* mutant animals in which the ERK/MAP kinase signaling could not be down-regulated. However, in contrast to starvation-effects on pharyngeal muscles, the starvation/pathogen induction of *ilys-3* occurs in the intestine. The transcriptional programs that govern pathogen- and starvation are likely to be distinct despite the fact that they share at least one downstream molecular target, *ilys-3*.

We discovered a cell non-autonomous regulation of the *ilys-3* transcriptional response following *M*. *nematophilum* infection. ERK expression in the pharynx alone was sufficient to restore intestinal *ilys-3* induction in the loss-of-function mutant *mpk-1;* while the activation of ERK MAP kinase in the intestine was not able to activate *ilys-3* transcription in this tissue. How can ERK signaling in the pharynx trigger *ilys-3* transcription in the gut? The ability of multicellular organisms to mount an efficient innate response against pathogen infections is likely to be aided by systemic communication between individual cells and tissues. In the intestine of *C*. *elegans* cell non-autonomous effects have been suggested in many contexts, ranging from the regulation of organismal response to oxidative and heat stress and lifespan [74–76] to the modulation of immune response against the bacterial pathogen *P*. *aeruginosa* [77]. In most of these examples however, the signaling between tissues requires a neuronal component. However, it has been shown that upon DNA damage, germ cells are capable of eliciting a systemic pathogen and stress resistance response in the somatic tissues of *C*. *elegans* [78]. Such induction depends on the germline specific activity of the ERK/MAK kinase *mpk-1*, which evokes the transcriptional activation of innate immune genes, similarly to the local response evoked by p38 MAPK upon intestinal infection by pathogens [68].

The results shown here suggest a communication between two non-neuronal distal tissues. This is reminiscent of a mammalian epithelial cell-to-cell communication strategy that propagates NF-kB and ERK- dependent pro-inflammatory signals from infected cells to bystander cells following infections by *Shigella flexneri* and *Listeria monocytogenes* [79,80]. However, in our context the signaling pathway that activates *ilys-3* in the pharynx is not required for its action in the other tissue.

In conclusion, our data suggest a model whereby upon *M*. *nematophilum* stimulation, the pharyngeal muscles activate the MAPK pathway leading to the transmission of an alarm signal to the intestinal epithelium. This results in the activation of an innate immune response, with transcription of the antimicrobial *ilys-3*. Whether the pharyngeal signal induces other intestinal responses, or acts on other distal tissues, remains to be seen. We also cannot exclude more complex models, in which the primary pathogen detection occurs in the intestine or elsewhere, and the danger signal is then amplified by the pharyngeal muscle. However, the pharynx is well placed to act as a site for primary danger detection.

Our work demonstrates the importance of a specific lysozyme gene both to normal nutrition and to defense. The unexpected complexity of its expression, both in different tissues and subcellular compartments, suggest that it may have functions within cellular vesicles, as well as in the digestive tract.

Our work also reveals the existence of a new tissue-tissue communication acting at the organismal level and reveals a strategy whereby the pharynx activates an antimicrobial intestinal defense by propagating bacterial/danger signals.

## Materials and Methods

### Worm culture and strains

Maintenance and manipulation of *C*. *elegans* strains were performed as previously described [81]. Unless otherwise specified, animals were cultivated at 20°C and fed with *Escherichia coli* OP50 as food. Wild-type is the *C*. *elegans* variety Bristol strain (N2). Mutant alleles were provided by the *Caenorhabditis* Genetics Center at the University of Minnesota, which is supported by the National Institutes of Health–Office of Research Infrastructure Programs (P40 OD010440).

The following genes and alleles were used in this work:

*LGI*: VC1026 [*rab-10(ok1494)*], GS2643 [*arIs37* [*myo-3p*::*ssGFP + dpy-20* (+) *I; cup-5(ar465) III; dpy-20(e1282) IV*]. DA541 [*gpb-2(ad541)*]. *LGIII*: CB6148 [*mpk-1(ku1)*], DP38 [*unc-119(ed3)*]. *LG IV*: VC2493 [*ilys-3(ok3222)*], obtained from the *C*. *elegans* Gene Knockout Consortium, outcrossed three times and sequence verified prior to phenotypic characterization. *LG V*: DH1201 [*rme-1(b1045)*]. Unknown LG: CB6603 *eIs102*[*egl-5p*::*GFP*::LIN-45*], RT311 {*unc-119(ed3); pwIs69*[*vha-6p*::*GFP*::*RAB-11 + Cbr-unc-119*(+)]}, RT476 {*unc-119(ed3); pwIs170*[*vha-6p*::*GFP*::*RAB-7 + Cbr-unc-119*(+)]}, NP871 {*unc-119(ed3) III; cdIs66*[*pcc1*::*GFP*::*RAB-7 + myo-2p*::*GFP* + *unc-119(+)*]}. The strain DA2200 carrying the integrated *adIs2200* [*myo-2p*::*MPK-1*::*GFP*] was a gift from Leon Avery, University of Texas Southwestern Medical Center, Dallas. NP717 strain carrying the *unc-119(ed3); arls37(myo-3p*::*ssGFP); cdls32*(*pcc1*::*DT-A(E148D)* + *unc-119(+) myo-2p*::*GFP* was a gift of H. Fares (University of Arizona, Tucson, Arizona). New strains constructed specifically for this study are listed in S5 Table.

### Bacterial strains

*E*. *coli* OP50, *E*. *coli* HB101 (DE3) and *E*. *coli*::GFP (kind gift of Jonathan Ewbank), *M*. *nematophilum* CBX102 and UV336; *M*. *luteus* DMS20030; *P*. *aeruginosa* PAO1. Bacterial cells were grown at 37°C in LB and lawns prepared from exponential phase growth cultures.

### Generation of reporter gene fusions and transgenic strains

Reporter constructs were generated using a PCR fusion protocol [82]. Except otherwise stated transgenic lines were obtained from injections of *unc-119(ed3)* mutants with the PCR products obtained from co-amplification of the promoters and the fluorescent proteins. The plasmid *unc-119* (+) (pDP♯MM016) was used as a genetic marker for the transgenes [83].

For *ilys-3p*::*GFP* transcriptional fusion, the *ilys-3* (*C45G7*.*3*) genomic sequence containing the 1.05 Kb 5′-upstream of the first predicted methionine was fused to the green fluorescent protein (GFP) and the *unc-54* 3′-untranslated sequence from the vector pPD95.75 (A. Fire). This amplicon was cloned into the pJET1.2 vector to give pMGN26. *eEx650* and *eEx651* are transgenic lines obtained from the injections in *unc-119(ed3)*. CB7163 contains *eIs120*, a gamma-integrated array derived from *eEx650*. This strain was outcrossed three times to the wild-type N2 before use. For the *ilys-1* reporter, the 1.7 Kb promoter of *ilys-1* (*C45G7*.*1*) was fused to DsRed2 from pMGN7 [18]. The PCR product was cloned into pJET1.2. Subsequent injections into *unc-119(ed3)* generated transgenic lines *eEx652* and *eEx653*. Transgene *eEx655* was obtained from the amplicon encompassing the 2.4 Kb upstream methionine sequence of *ilys-2* (*C45G7*.*2*) fused to CFP from pPD133.51. For the *ilys-4p*::*GFP* transcriptional reporter the 3.3 Kb upstream sequence of *ilys-4* (*C55F2*.*2*) was fused to GFP from vector pPD95.75. *eEx670* is the transgene obtained. For the *ilys-5* transcriptional reporter the 5.8 Kb sequence upstream methionine of *ilys-5* (*F22A3*.*6*) was fused to GFP from pPD95.75. The amplicon was cloned into pJet1.2. Transgenes obtained from *unc-119* injections were: *eEx671* and *eEx672*.

To generate the translational fusion of *ilys-3p*::*ILYS-3*::*mCherry g*enomic DNA fragment encompassing the full length ILYS-3 (4.5 Kb promoter and 925 bp ORF) was fused at the C-terminal with the mCherry amplicon from pFP10. The resulting PCR was cloned into Topo XL vector (Invitrogen) to give pMGN47 and injected into CB7029 [*ilys-3(ok3222); unc-119(ed3)*] or into CB7007 [*ilys-3(ok3222)*] adult worms at a concentration of 20 ng/μl. For the injections into *ilys-3(ok3222)* the co-injection marker *sur-5*::*GFP* (pTG96) was used at 60 ng/μl. Transgenic lines *eEx752*, *eEx753* and *eEx754* contain extrachromosomal versions of pMGN47.

To construct N-terminal *GFP*::*ILYS-3* transgene driven by its own promoter the GFP vector pPD117.01 was used (Andy Fire). For this the 4.5 Kb *ilys-3* promoter was PCR amplified with oligonucleotides containing *XbaI* and *Not*I specific sites. The resulting amplicon was subcloned into pJet1.2 to generate pMGN50. The *ilys-3* promoter was then restriction digested and cloned into the *Xba*I *Not*I sites of pPD117.01 to generate the plasmid 4.5 Kb *ilys-3p*::*GFP*, pMGN52. The entire coding sequence of ILYS-3 containing its own 3'UTR was obtained from N2 genomic DNA and amplified with oligonucleotides containing *Nhe*I and *Apa*I specific sites. The amplicon was subcloned into pJet1.2 to give pMGN51, and then excised and directionally cloned into the *Nhe*I and *Apa*I sites, downstream of GFP, into plasmid pMGN52. To establish transgenic lines, 20 ng of the resulting plasmid, pMGN53 was microinjected into the *unc-119* mutant along with the *unc-119* co-injection marker. Transgenic line *eEx779* contains extrachromosomal version of pMGN53 to give CB7210.

### Tissue specific constructs

Construct pMGN37 was generated by fusion of the 598 bp sequence of the intestinal specific promoter *mtl-2* to *mpk-1* cDNA from plasmid pHN3e [11], The resulting amplicon was cloned into pJet1.2. The *mtl-2* promoter was also fused to mCherry to give pMGN39. These two plasmids were co-injected at 5 ng/μl into *unc-119(ed3)* and the transgenic line *eEx727* was crossed into *mpk-1(ku1)* to give CB7004. Complete plasmid sequences are available upon request.

### Heat-killing bacterial assays

For heat-killing of OP50 and CBX102, fresh overnight cultures were concentrated 10-fold and incubated at 100°C for 60 min. Following heat treatment, cells were plated to confirm inviability after overnight incubation on LB plates. For the assays,100 μl of dead bacterial cell suspensions were plated on NGM plates and approximately 50 L1 larvae from the test strains WT, *ilys-3*, or *ilys-3p*::*GFP* reporter were placed on each plate. Animals were analyzed from triplicate plates and experiments were repeated twice each with freshly prepared bacterial cells.

### Endocytosis assays

The fluid-phase marker BSA::Alexa 488 (Thermo Fisher Scientific), was injected at 1 mg/ml into the pseudocoelomic space in the pharyngeal region of adult worms as described by Zhang *et al* [84]. Injected worms were transferred to OP50 seeded plates at 20°C, and observed at different time points. The intracellular trafficking of the dye was stopped by moving the plates to ice. Injected animals were subsequently mounted on agarose pads and observed on the confocal microscope.

### Quantitative RT-PCR analysis

qRT-PCR was performed in synchronized populations obtained by the alkaline bleach method using gravid hermaphrodites and letting the eggs hatch overnight in M9 on NGM peptone-depleted plates. Synchronized naïve L1 larvae were transferred to bacterial lawns made of 100% OP50, *M*. *nematophilum* CBX102, UV336 or *M*. *luteus* DMS20030 and harvested 24 or 48 hours later. In each case, total RNA from triplicate samples were extracted using Trizol (Thermo Fisher Scientific). RNA concentration and quality was assessed with a NanoDrop 1000 spectrophotometer (Thermo Fisher Scientific). 1μg of total RNA from infected and non-infected worms was used for reverse transcription (SuperScript VILO, Thermo Fisher Scientific), and subjected to RNase H (NEB) treatment subsequently. mRNA levels were assessed using a duplexing qPCR with the TaqMan Gene expression assay (Applied Biosystems). This assay relies on two fluorescently labeled TaqMan probes: the two reporter dyes FAM and VIC were used for the detection of the gene of interest and endogenous control, respectively. Using the two probes the method allows the simultaneous amplification and quantification of two target sequences in the same sample. Fluorescent reporters were detected on the real-time qPCR platform StepOnePlus (Applied Biosystems). Results were normalized to *E*. *coli* OP50 and to the endogenous control gene *rla-1*, which did not vary under the conditions being tested. Expression levels were assayed using the 2^-ΔΔCt^ method [85]. Means and standard error of the mean were calculated from at least 3 independent biological replicates. Statistical analysis was done using Holm-Sidak's multiple comparisons test. Primer sequences are available upon request.

### Infections

Unless otherwise stated infections with *M*. *nematophilum* and *M*. *luteus* were carried out by adding synchronized L1 larvae to 100% bacterial lawns followed by incubation at 25°C. Surviving worms were counted every day.

### Analysis of live bacteria accumulation

To analyze bacterial accumulation in the intestine, wild-type, *ilys-3*(*ok3222)* and *ilys-3; eEx752* animals were synchronized and allowed to grow to L4 on OP50 seeded plates. L4 were then transferred to NGM plates seeded with *E*. *coli*::GFP or CBX102 and grown at 20°C for 24 hours. For the *E*. *coli*::GFP bacterial counts, pools of 10 worms each were surface sterilized with 100 μg/ml gentamicin in PBS + 0.1% Triton X-100 (PBST) and subsequently washed in 10 μl drops of ice-cold 25mM tetramisole/M9 (0.1% Triton X-100). Manual disruption of the worms was carried out in PBST in a sterile 1.5 ml microcentrifuge tube using a pestle and glass beads. Triplicates of worm lysates corresponding to 10 animals per group were serially diluted in PBS and incubated overnight on LB ampicillin plates to determine the number of colony forming units (CFU). For CBX102 bacterial counts, pools of 10 worms each were sterilized with 50 μg/ml nalidixic acid in PBST, serially diluted, and bacterial suspensions plated on LB agar supplemented with nalidixic acid. Cultures were incubated at 37°C and colonies counted after 48 hours.

### SYTO 13 staining

Bacterial cells of CBX102 were labeled with SYTO 13 (ThermoFisher Scientific), at a final concentration of 15 μM in 10mM Tris-HCl, 5 mM NaCl, 1 mM EDTA, pH7.5. Briefly, fresh overnight cultures were concentrated 5-fold and bacteria were stained with SYTO 13 for 45 min at room temperature. Aliquots (100 μl) of stained bacterial suspensions were added to NGM plates, approximately 50 one-day old adults from the test strains WT, *ilys-3*, or *ilys-3; eEx752* were placed on each plate and animals were allowed to fed for 2 hours.

### Lifespan assays

Before being assayed, worms were fed for two generations on HB101 and synchronized twice by bleaching. The resulting eggs were left overnight to hatch at 20°C in M9 on NGM peptone-depleted pates. For lifespan analysis, Day 0 was the day animals were exposed to bleach for synchronization. Assays were conducted at 20°C or 25°C. Approximately 100 animals were tested in each experiment (25 animals/ NGM plate without FUdR). During the first few days, worms were transferred to fresh plates daily. Animals were scored every other day, as alive or dead by gentle prodding with an eyelash. For *ilys-3; eEx754* and +; *eEx754*, only GFP positive animals were analyzed. Worms that crawled off the plate were censored. Statistical analysis was performed using the software Prism 6 (GraphPad) and survival data were compared using the log-rank test.

### MAP kinase inhibition using U0126

Synchronized *eEx650* L1 transgenic animals were left on *E*. *coli* HB101 or 10% *M*. *nematophilum* CBX102:HB101 for 48 hours and allowed to develop to late L4. These were then transferred to HB101 or CBX102:HB101 (at 1:10 ratio) NGM seeded plates containing either 50 μM U0126 (Sigma) or DMSO (as a vehicle control). Plates were incubated for 48 hours at 25°C and fluorescence intensity in the intestinal cell int8 was measured using confocal microscopy.

### Microspheres feeding assay

For the microsphere feeding assay, adults and dauers carrying the transgene *eEx752 ILYS-3*::*mCherry* reporter construct were transferred to NGM plates containing overnight lawns of OP50 mixed with Fluoresbrites yellow-green microspheres, 0.5 μm (Polyscience, Inc.) at a 1:1000 ratio (v/v) of beads to bacteria. Animals were allowed to feed for at least 45 min or longer, anesthetized in a drop of 100 mM tetramisole and mounted on agarose pad for imaging.

### Starvation assays

After bleaching, synchronized L1 worms carrying the *ilys-3p*::*GFP* reporter were transferred to peptone-depleted NGM plates and let starve for a specific amount of time (typically between 24–72 hours from transferal). For the L4 nutrient depletion assays, well-fed late L4 animals were washed five times in M9 to exhaust any residual bacterial cells, and subsequently transferred to unseeded peptone-depleted NGM plates where they remained till they were collected for light microscopy analysis. The fluorescence intensity of intestinal GPF of nutrient depleted animals was then compared to that of chronologically identical animals reared under normal and well fed conditions. Mean fluorescence value was subtracted from the background fluorescence for each animal and statistical analysis was performed using a Mann Whitney test, 95% confidence interval relative to controls.

### LysoTracker Green staining

The lysosome marker LysoTracker Green (ThermoFisher Scientific) was used at 1 μM concentration and 200 μl of green dye were added to 9 cm OP50 seeded-NGM plate and left to stabilize for 24 h. Animals were allowed to feed on labeled *E*. *coli* for 2 days and imaged as young adults.

### Quantification of fluorescence intensity in the intestine

Quantification of the *ilys-3p*::*GFP* fluorescence in the intestinal cells int2 and int8 of reporter transgenes was carried out using a Leica TCS-SP5 Laser Scanning Confocal microscope using a x63 oil immersion lens and the Argon 488 laser. The focal plane with the highest GFP signal was used to measure fluorescence intensity within a ROI set to 0.4 μ thickness, and a 10 μ or 40 μ diameter, for L1 larvae or adults, respectively. To make comparisons across samples, data are presented in boxes plots which define interquartile range (25% of the data above or below the median), bars represent expression range, and the thick line is the median. Identical exposure settings were used for all genotypes. GFP fluorescence was limited to 495/512 nm to diminish background autofluorescence from the animals. For each experiment, and on the same day, 10–15 animals/ treatment were imaged. GFP/mCherry colocalization assays were performed on adult worms expressing the fluorescent markers as previously described. The Pearson's coefficients were calculated using Leica LAS AF software. This correlation coefficient has been used to calculate the percentage of pixels that colocalize from each channel. For imaging ILYS-3::mCherry worms were examined under the HeNe 543 nm laser line.

### RNAi assays

Transgenic animals expressing the *ilys-3p*::*GFP* reporter were used in the RNAi feeding assay performed as described [86] using bacteria from the Ahringer RNAi library (Source BioScience) grown overnight in Luria broth supplemented with 100 μM ampicillin and 25 μM tetracycline, and 100 μl of the culture was added to NGM plates supplemented with 1 mM isopropyl β-D-1-thiogalactopyranoside, 30 μM carbenecillin. Five L4 animals were transferred to the RNAi lawn and allowed to self-fertilize. The F1 progeny was added to OP50 or CBX102 lawns and phenotypes analyzed. RNAi experiments were replicated twice, each with freshly prepared medium. The empty vector L4440 was used as control RNAi.

### Recombinant ILYS-3 expression MBP fusion

A construct was engineered that allowed expression of the full length of ILYS-3 (139 amino acids) fused with *E*. *coli* maltose-binding protein (MBP, 367 amino acids) via a 47 amino acid-long linker. The maltose-binding-protein (MBP) fusion system is reported to enhance solubility and proper folding of recombinant proteins. The recombinant pMAL-c2X::ILYS-3 protein coding sequences were verified by DNA sequencing. The resulting MBP-rILYS-3 fusion protein (58 kDa and MBP alone 43 kDa) was overexpressed in *E*. *coli* BL21 (DE3) and induced with 1 mM IPTG for 3 hours at 37°C and the cells were harvested by centrifugation at 5000 *g* for 20 min and disrupted by a French pressure cell. The homogenates were cleared at 20,000 *g* for 25 min and affinity purified using an amylose resin column and washed with binding buffer following manufacturer's instructions (NEB).

A second (r)ILYS-3 was also prepared with a DNA fragment containing ILYS-3 coding region excluding the first 19 aa signal sequence, and containing *BamH*I and *Xho*I restriction sites. The fragment was cloned into the expression vector pGEX-6T1 (GE Healthcare) which contains a N-terminal GST affinity tag. The resulting plasmid pMGN73 (GST::ILYS-3 signal less) was verified by sequencing. *E*. *coli* BL21 cells were transformed with pMGN73 and grown in LB containing ampicillin and 1% glucose and incubated at 30°C, until cell density reached OD_600_ 0.6. To induce the expression of GST::ILYS-3 fusion protein, 0.2 mM IPTG was added and incubation was continued by shaking for an additional 4 h at 23°C. Recombinant GST::ILYS-3 signal less expressed as inclusion bodies and these were solubilized in 8M urea. After solubilization, the fusion protein was refolded by stepwise dialysis for 48 h at 4°C against 50 mM Tris, pH 8.0, 10% glycerol, 50 mM NaCl, 10 mM glutathione reduced, 1 mM glutathione oxidized to drop urea to 0 M concentration. The refolded GST::ILYS-3 signal less fusion protein was subsequently dialyzed against 25 mM Tris, pH 8.0, 10% glycerol, 50 mM NaCl to remove glutathione and purified using a GST Spin Trap column pre-equilibrated with binding buffer (GE Healthcare) following manufacture's instructions.

### Zymogram

The purified recombinant proteins were analyzed by performing a zymogram assay based on an SDS–PAGE and subsequently stained with Coomassie Blue. Two mg/ml of lyophilized *M*. *luteus* or *M*. *nematophilum* cells, were resuspended and incorporated into a 10% SDS-PAGE gels as in [56]. 15 μl samples were loaded into each well. After electrophoresis gels were washed three times in dH_2_O for 30 min and then renatured for 16 hours in 250 mM Tris pH (ranged from 3.0–7.0), 20 mM glycine and 100mM NaCl at 37°C, under constant agitation. Next day, gels were stained in 1% methylene blue for 1 hour, and destained in dH_2_O until clear bands emerged. Methylene blue staining of the gel reveals sites of hydrolysis as white bands on an otherwise dark blue background.

### Microscopy

Animals were anesthetized with 10 mM tetramisole/M9 buffer mounted on 2% (w/v) agarose pads. Images were captured by differential interference contrast (DIC) and epifluorescence microscopy on a Zeiss Axioplan 2 fluorescence microscope using a Zeiss AxioCam camera, or by confocal microscopy using a Leica SP5. Images captured using an AxioCam camera and AxioVision software (Carl Zeiss).

### Statistical analysis

Statistical analysis and graphing was done on Prism 6 (GraphPad Software, San Diego, CA).

## Supporting Information

S1 Fig Invertebrate lysozymes express in the digestive tract and nervous system. Analysis of the promoter activity of the *C*. *elegans* invertebrate lysozymes *ilys-1*, *-2*, *-4*, *-5* and *-6* using DsRed2, CFP and GFP proteins. Fluorescence micrographs of transgenic animals expressing the *ilys* transcriptional reporters. (A) *ilys-1* expression in the pm3 in the procorpus, in the pm4 cells in the metacorpus and the marginal cells mc1 and mc2. (B-C) *ilys-2* expression in the muscle pm3, nerve ring (arrowhead) and in the intestine (int) (arrow). (D-F) Expression of *ilys-4* in the interneurons in the head, in the ventral nerve cord (VNC) and intestine (int). (G-H) *ilys-5* expression in the presumptive I1 and AIM/AIY neurons and intestine (int). (I-K) Expression of *ilys-6* in the pharyngeal gland cells (ph gland) and duct projections (arrowhead), coelomocytes (cc) and intestine (int).
(TIF)

S2 Fig ILYS-3::mCherry expresses in the epidermis. (A) Fluorescence image of ILYS-3::mCherry in the epidermis (arrow) in dauer. (B) Overlay of Nomarski and fluorescence images.
(TIF)

S3 Fig ILYS-3::mCherry expresses in tubular-vesicular recycling network of the intestinal cells animals on CBX102. (A) Micrograph taken from top focal plane, showing ILYS-3::mCherry positive vesicles and tubules in the basolateral compartment. Arrow marks the tubular network in the intestine of an adult hermaphrodite grown on CBX102. (B) Micrograph acquired in the middle focal plane of the intestine, showing ILYS-3::mCherry positive basolateral vesicles in an adult grown on CBX102 and fed with fluorescent microspheres here illustrating the lumen of the intestine. The contours of the basolateral compartment are outlined (dashed lines). Cc: coelomocytes.
(TIF)

S4 Fig qRT-PCR analysis of the induction of ILYS genes in response to the infection by the Gram-positive *M*. *nematophilum* CBX102 and the Gram-negative *P*. *aeruginosa* PAO1 pathogens. Graph shows the relative induction of *ilys-2*, *-3* and *-6* expression following exposure to OP50, CBX102 and *P*. *aeruginosa* (PAO1) for 24 hours. mRNA levels were normalized to *E*. *coli* OP50, and to the endogenous control gene *rla-1*. *ilys-2* and *ilys-3* transcripts, and to a less extent *ilys-6*, were responsive to *M*. *nematophilum* CBX102 but not to *P*. *aeruginosa* PAO1. Gene expression was analyzed using the comparative ΔΔCt method. Data are representative of 2 independent experiments. Error bars denote SEM. ***: indicate statistically significant differences of the indicated comparisons. Data were analyzed with two-way Anova, Holm-Sidak's multiple comparison tests (99% CI). CBX102 induced significantly higher levels of *ilys-2* and *-3* than PAO1 (*** *p* < 0.0001). In contrast, expression levels of *ilys-6* were not significantly different (*p* = 0.2876, NS).
(TIF)

S5 Fig Heat-killed bacteria induce strong expression of the *ilys-3* reporter in the intestine. (A-B) Images of *ilys-3* reporter animals fed on live or dead bacteria for 48 hours, after bleaching. Worms were added to NGM plates as synchronized L1 larvae. (A) Details of the GFP expression in animals grown on OP50. (i) and (ii) basal *ilys-3p*::*GFP* expression in worms on live OP50. (iii-iv) Strong induction in animals on dead OP50. (B) Details of GFP induction on CBX102. (i-ii) High GFP expression in the intestine of animals on live bacterial cells as well as (iii-iv) on dead CBX102. (C) Quantification of the *ilys-3p*::*GFP* fluorescence in the intestinal cell int2 of the *ilys-3* reporter in animals fed on live or heat-killed OP50 or CBX102 for 48 hours. ROI was set to 20 μ diameter and 0.4 μ thickness. Graph is representative of two independent experiments. Asterisks indicate the results of Mann Whitney test of fluorescence values, 99% confidence interval. Fluorescence intensity for worms on heat-killed_OP50 *vs* live_OP50 differ significantly (****p < 0.0001). Mean values for animal heat-killed_CBX102 vs live_CBX102 and heat-killed_OP50 *vs* heat-killed_CBX102 were not significantly different (*p* = 0.0715 and *p* = 0.0717, respectively). NS: not significant. N = 12 per group.
(TIF)

S6 Fig The expression of *ilys-5* decreases in transgenic animals subjected to starvation for 24 hours. (A-B) One-day old adult animals carrying the *ilys-5p*::*GFP* reporter and grown on OP50. Basal levels of GFP expression can be detected in their intestines. (C-D) Fluorescent images of representative transgenic animals that were transferred to nutrient-depleted plates at L4 and imaged 24 hours latter. The high intestinal *ilys-5p*::*GFP* signal is almost completely abrogated in worms grown in the absence of bacteria.
(TIF)

S7 Fig Inactivation of *ilys-3* exerts a modest effect on the normal reproduction on OP50. Total number of progeny counted for individual wild-type (N2) or *ilys-3* mutants. Average brood sizes are expressed as a percentage of wild-type (error bars indicate SEM). Asterisks indicate ***p < 0.0001 (two-tailed unpaired t-test). Loss of *ilys-3* reduced the brood by 10% only. [Broods: N2 = 269 *vs ilys-3* = 241].
(TIF)

S8 Fig *ilys-3* mutants and WT exhibit similar developmental rates on live bacteria diets. Plot represents developmental progression on live cells of OP50 and CBX102. Synchronized N2 and *ilys-3* animals (at L1 stage) were grown on the two different diets as indicated on the x-axis, and scored after 48 hours.
(TIF)

S9 Fig ILYS-3 expresses in lysosomes (LY) and late endosomes (LE) in coelomocytes. **(**A) ILYS-3::mCherry and BSA::Alexa 488 colocalization. (B) Vacuoles accumulating ILYS-3::mCherry in the *cup-5(ar465)* mutant. Green shows endocytosed GFP (*myo-3p*::ss*GFP*). (C) Deficient endocytosis does not block ILYS-3::mCherry in the coelomocytes in the *rme-1(b1045)* mutant. (D-F) Some vesicles ILYS-3::mCherry-labelled colocalize with the RAB-7 GFP-marked late endosomes and lysosomes. (D) Red channel. (E) Green channel. (F) Overlay of the images of the corresponding red and green channels.
(TIF)

S10 Fig Effect of coelomocyte depletion in *ilys-3* mutants. (A) Lifespan analysis of N2, *ilys-3(ok3222)*, NP717 (coelomocyte-depleted), CB7137 [*ilys-3(ok3222);* coelomocyte depleted double mutants] fed on *M*. *nematophilum* from day 0 after bleaching and raised at 20°C. *P* value *vs* control calculated with the Mantel-Cox log-rank test (95% CI). Results are the mean of 2 independent trials. N ranges from 220 to 150. (B) Table summarizing the details of lifespan analysis.
(TIF)

S11 Fig *mpk-*1 RNAi abrogates *ilys-3p*::*GFP* induction in the intestine of CBX102-exposed adults. (A) Green fluorescence in transgenic worms carrying the *ilys-3p*::*GFP* reporter fed on the control RNAi L4440. (B) *mpk-1* RNAi blocks the green fluorescence of the *ilys-3* reporter in the intestine but not in the pharynx (arrows).
(TIF)

S12 Fig Quantification of *ilys-3p*::*GFP* expression in the *gpb-2(ad542)* genetic background upon exposure to OP50 or CBX102 Quantification of fluorescence intensity in the intestinal cell int8 of one-day-old adults. ROI set to 40 μ diameter and 0.4 μ thickness. Graph is representative of two independent experiments. Asterisks indicate the results of Mann Whitney test of fluorescence values, 95% confidence interval. Fluorescence intensity for *gpb-2; ilys-3p*::*GFP*_OP50 *vs ilys-3p*::*GFP*_OP50 and *gpb-2; ilys-3p*::*GFP_*CBX102 *vs ilys-3p*::*GFP* _CBX102 differ significantly (** *p* = 0.0052 and *** *p* = 0.0004, respectively).
(TIF)

S13 Fig The activation of ILYS-3 does not require MPK-1 activity in the gut. **Fluorescence quantification of *ilys-3* promoter activity and the effect of intestinal MPK-1 in int8.** The construct *mtl-2p*::*MPK-1* drives MPK-1 expression in the intestine. In the *mpk-1* mutants, *ilys-3* expression was blocked. This phenotype was not rescued when MPK-1 is restored in the intestine. Asterisks indicate the results of a Mann–Whitney Unpaired test statistical comparisons of the fluorescence intensity for *mpk-1(ku1); ilys-3p*::*GFP; mtl-2p*::*MPK-1 vs ilys-3p*::*GFP; mtl-2p*::*MPK-1* and *mpk-1(ku1); ilys-3p*::*GFP vs ilys-3p*::*GFP* (*** *p* = 0.0002), *mpk-1(ku1); ilys-3p*::*GFP; mtl-2p*::*MPK-1 vs ilys-3p*::*GFP* (** *p* = 0.0012), which all differ from their controls. Mean values for *mpk-1* mutants with the double transgene were not significantly different (NS) from their sibling controls harbouring the *ilys-3p*::*GFP* reporter only (*p* = 0.7137). N ≥ 15/group.
(TIF)

S14 Fig Overexpression of the active form of *LIN-45* (Raf) in the rectal epithelium results in tail swelling in the absence of *M*. *nematophilum* infection but is not sufficient to promote induction *ilys-3p*::*GFP* expression in the intestine. **(**A-B) Image of an adult showing the active LIN-45* driven by the 1.3 Kb *egl-5* promoter fragment which expresses GFP in the B, K, F, U, and P12.pa rectal epithelial cells and in three posterior body wall muscles. (A) Fluorescent channel (B) Merge image of GFP and DIC. (C-D). Representative image of a transgenic animal expressing intestinal *ilys-3p*::*GFP*. (D). Overlay. (E-F). Representative image of an animal bearing the two transgenes, showing that intestinal *ilys-3p*::*GFP* expression is not enhanced by constitutively activating MAPK signalling in the rectal epithelium. (F) Merge image of GFP and DIC. All images correspond to 1-day-old adults.
(TIF)

S15 Fig The activation of ILYS-3 requires MPK-1 activity in the pharynx. Fluorescence quantification of *ilys-3* promoter activity and the effect of pharyngeal MPK-1 in int8 of single and double transgene reporter strains. The construct *myo-2p*::*MPK-*1 drives MPK-1 expression in the pharynx and rescued the intestinal *ilys-3* expression in *mpk-1* mutants. Data analyzed with Mann–Whitney Unpaired test, 95% confidence level. Fluorescence intensity for *mpk-1(ku1); ilys-3p*::*GFP; myo-2p*::*MPK-1 vs ilys-3p*::*GFP; myo-2p*::*MPK-1* and *mpk-1(ku1); ilys-3p*::*GFP; myo-2p*::*MPK-1 vs ilys-3p*::*GFP* were not significantly different (ns). Mean values for *mpk-1* mutants with the double transgene differ significantly from their sibling controls harbouring the *ilys-3p*::*GFP* reporter only (** *p* = 0.004). N = 10-15/ group.
(TIF)

S16 Fig Regulation of *ilys-3 mRNA* upon *M*. *nematophilum* infection in *mpk-1(ku1)* mutants by pharyngeal but not intestinal MPK-1 activity. RT-PCR quantification of relative levels of *ilys-3* in *mpk-1(ku1)* mutants and control strains grown on OP50 or CBX102. Levels were normalized against WT (N2) on *E*. *coli* and to the endogenous control gene *rla-1*. Gene expression was analyzed using the comparative ΔΔCt method. Each bar represents the average relative mRNA level from three independent RNA isolations obtained from synchronization L1 in populations of worms exposed for 24 hours to *M*. *nematophilum*. Control strains were as follows: N2, *ilys-3p*::*GFP; unc-119; unc-119 (+)*, *mpk-1(ku1); unc-119(+)* [obtained from cross with *mpk-1; mtl-2p*::*MPK-1; unc-119; unc-119(+)*], and *mtl-2p*::*MPK-1; unc-119; unc-119(+)*, *myo-2p*::*MPK-1*. Data were analyzed with two-way Anova, Holm-Sidak's multiple comparison tests, 99% confidence interval. Error bars represent SEM. Expression levels of *ilys-3* were significantly higher in transgenic animals expressing the pharyngeal MPK-1 relative to N2 controls regardless of nature of the bacterial lawn (*** *p* < 0.0001). The mean values for *mpk-1* mutants with the intestinal MPK-1 transgene were not significantly different from their sibling single mutant controls (NS: *p* > 0.9999). For multiple comparisons see S4 Table.
(TIF)

S17 Fig Following nutrient depletion intestinal *ilys-3* induction does not depend on MPK-1 signaling. Fluorescence images of *ilys-3p*::*GFP* reporter in worms fasted for 24 hours. (A) *ilys-3p*::*GFP* control. (B) *ilys-3p*::*GFP*; *mpk-1* mutants showed similar intestinal fluorescence intensity relative to control animals.
(TIF)

S18 Fig The *C*. *elegans* ILYS-3 closely resembles bivalve i-type lysozymes. (A) Predicted 3D homology model structure of the *C elegans* ILYS-3 inferred from the crystal structure from of the lysozyme isolated from *M*. *lusoria* (PDB No:3AB6A). This model was created using PyMOL molecular graphic software. Residues E18 D29 predicted to be involved in muramidase activity are shown in red. Residues predicted to be responsible for the isopeptidase activity are marked in green. Of these only H113 seemed to have been conserved in both lysozymes. The other residue is a substitution of the S77 to A. (B) Sequence logo showing the conservation of amino acids in i-type lysozymes of *Meretrix lusoria*: 3AB6, *Tapes japonica*: 2DQA and *C*. *elegans* ILYS-3, based on a multiple sequence alignment (CLUSTALW) WebLogo3 was used to generate the sequence logos. Signal peptides were predicted by SignalP 4.1 server and removed prior to alignment. Poorly aligning N- and C- terminus were also removed. Amino acids are colored by charge: blue: positive and red: negative. The two muramidase residues E and D are indicated by red arrows and the possible active residues responsible for isopeptidase activity are marked with blue arrowheads. In contrast to the *Tapes japonica* protein, endowed with both the Serine and Histidine residues that confer isopeptidase activity, the *C*. *elegans* ILYS-3 and the *M*. *lusoria* 3AB6A appeared to have replaced the active Serine (S77) with Alanine.
(TIF)

S1 Table A summary of the distinct patterns of expression for all the *ilys* reporters constructed in this study. (DOCX)

S2 Table Survival rates of N2 and *ilys-3(ok3222)* deletion mutants with or without extrachromosomal arrays *eEx752* and *eEx754* on lawns of OP50 from day 1 after bleaching, raised at 20°C. *P* value *vs* control calculated with the Mantel-Cox log-rank test (95% CI). Results are the mean of 3 independent trials.
(DOCX)

S3 Table Survival rates of N2 and *ilys-3(ok3222)* deletion mutants with or without extrachromosomal arrays *eEx752* and *eEx754* on lawns of CBX102 from day 0 after bleaching, raised at 25°C. *P* value *vs* control calculated with the Mantel-Cox log-rank test (95% CI). Results are the mean of 3 independent trials.
(DOCX)

S4 Table Real Time-PCR quantification of the effect of pharyngeal and intestinal MPK-1. (DOCX)

S5 Table List of *C*. *elegans* strains constructed for this study. (DOCX)

S6 Table Gene accession information (DOCX)

S1 Video Expression of the *C*. *elegans ilys-4* in a set of interneurons in the head, the presumptive AVE/AVA and RIG/ALA cells, in the ventral nerve cord and the intestine. (MOV)

S2 Video Expression of the *C*. *elegans ilys-5* in the presumptive I1 and AIM/AIY neurons and intestine. (MOV)
