# Supplementary figures and images for: The Invertebrate Lysozyme Effector ILYS-3 Is Systemically Activated in Response to Danger Signals and Confers Antimicrobial Protection in C. elegans
Source: PLoS Pathog. 2016 Aug 15;12(8):e1005826. doi: 10.1371/journal.ppat.1005826 (PMC4985157; doi:10.1371/journal.ppat.1005826)

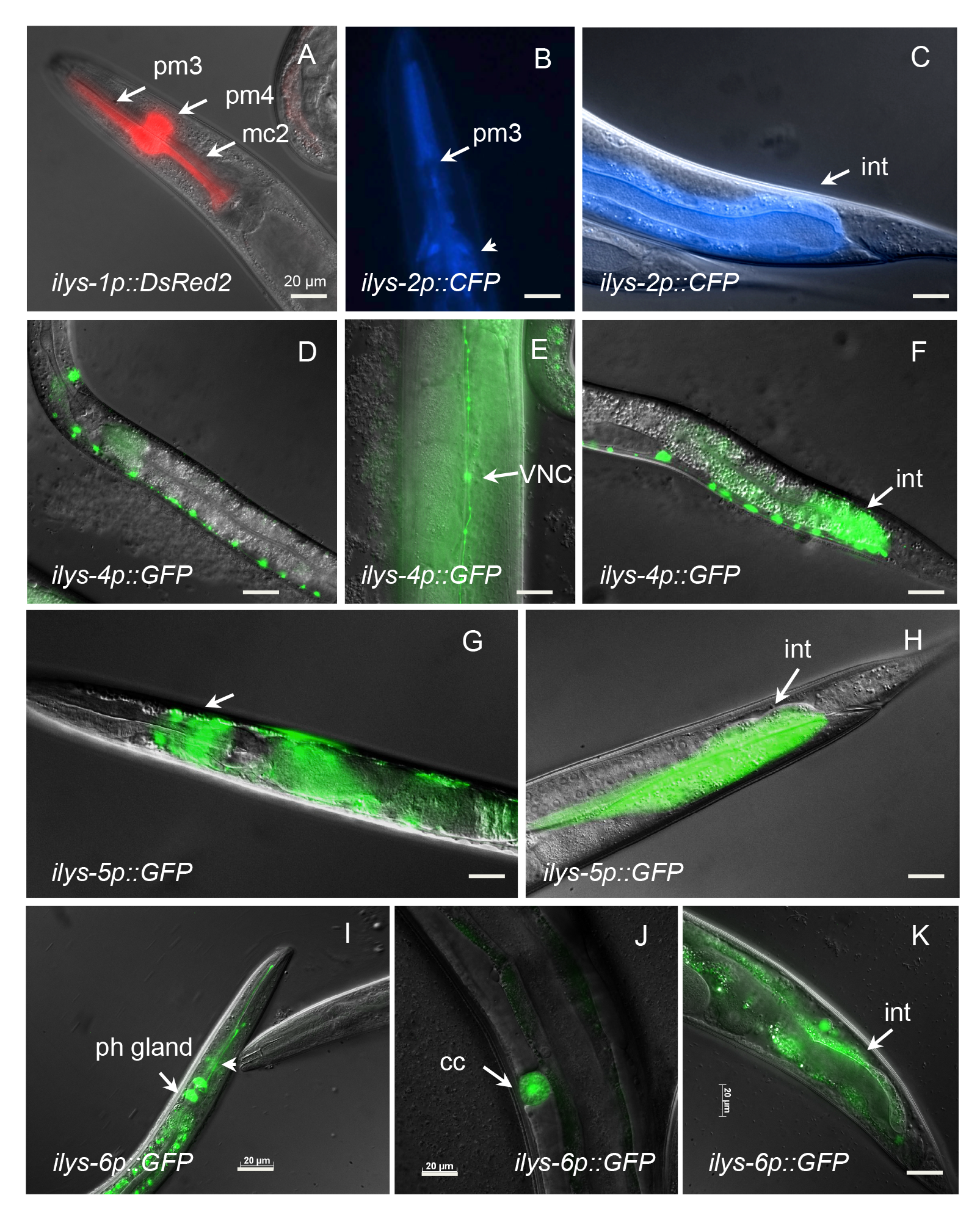

Supplement: S1 Fig — Analysis of the promoter activity of the C. elegans invertebrate lysozymes ilys-1, -2, -4, -5 and -6 using DsRed2, CFP and GFP proteins. Fluorescence micrographs of transgenic animals expressing the ilys transcriptional reporters. (A) ilys-1 expression in the pm3 in the procorpus, in the pm4 cells in the metacorpus and the marginal cells mc1 and mc2. (B-C) ilys-2 expression in the muscle pm3, nerve ring (arrowhead) and in the intestine (int) (arrow). (D-F) Expression of ilys-4 in the interneurons in the head, in the ventral nerve cord (VNC) and intestine (int). (G-H) ilys-5 expression in the presumptive I1 and AIM/AIY neurons and intestine (int). (I-K) Expression of ilys-6 in the pharyngeal gland cells (ph gland) and duct projections (arrowhead), coelomocytes (cc) and intestine (int). (TIF) [file ppat.1005826.s001.tif]

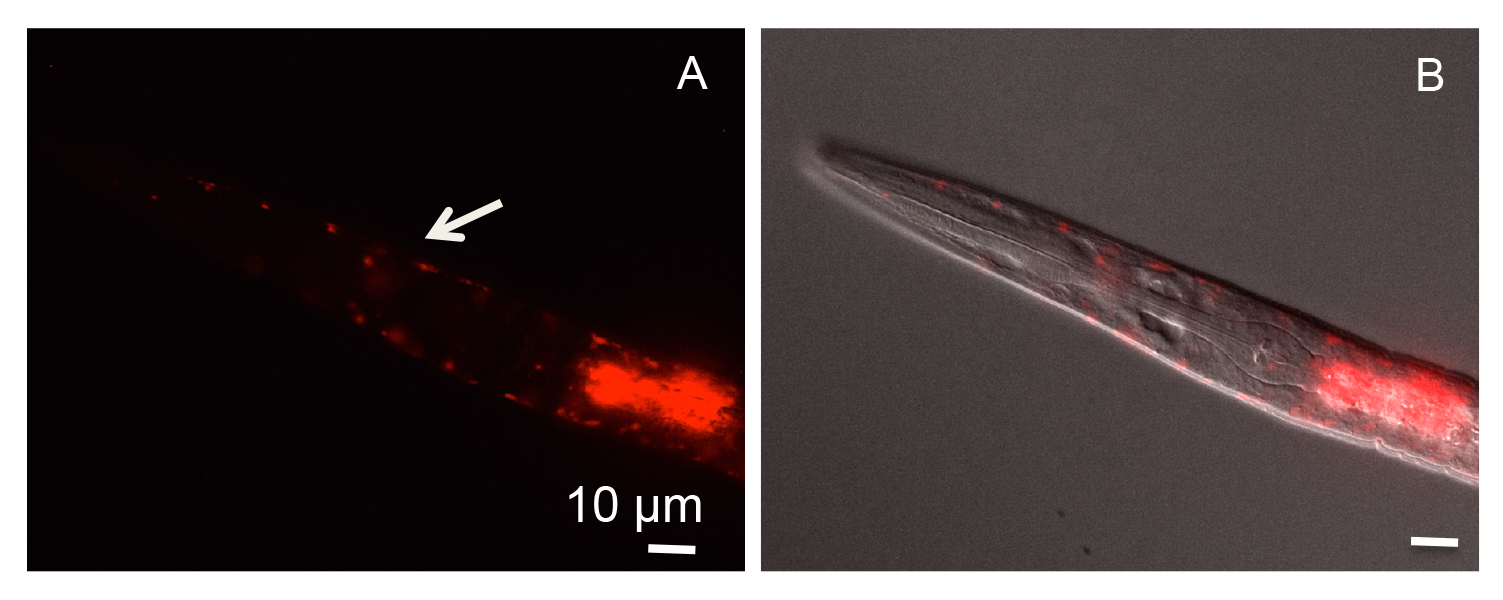

Supplement: S2 Fig — (A) Fluorescence image of ILYS-3::mCherry in the epidermis (arrow) in dauer. (B) Overlay of Nomarski and fluorescence images. (TIF) [file ppat.1005826.s002.tif]

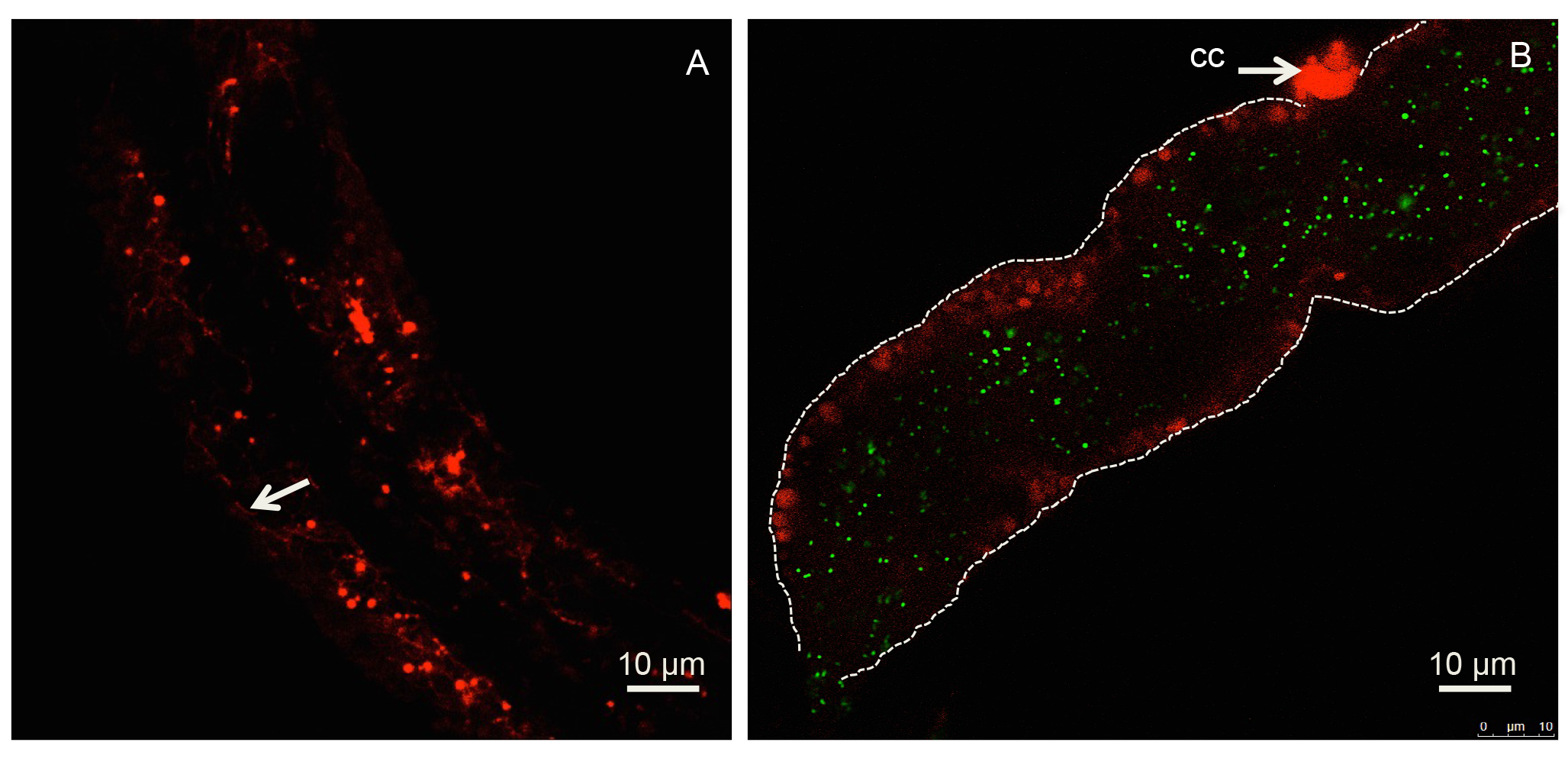

Supplement: S3 Fig — (A) Micrograph taken from top focal plane, showing ILYS-3::mCherry positive vesicles and tubules in the basolateral compartment. Arrow marks the tubular network in the intestine of an adult hermaphrodite grown on CBX102. (B) Micrograph acquired in the middle focal plane of the intestine, showing ILYS-3::mCherry positive basolateral vesicles in an adult grown on CBX102 and fed with fluorescent microspheres here illustrating the lumen of the intestine. The contours of the basolateral compartment are outlined (dashed lines). Cc: coelomocytes. (TIF) [file ppat.1005826.s003.tif]

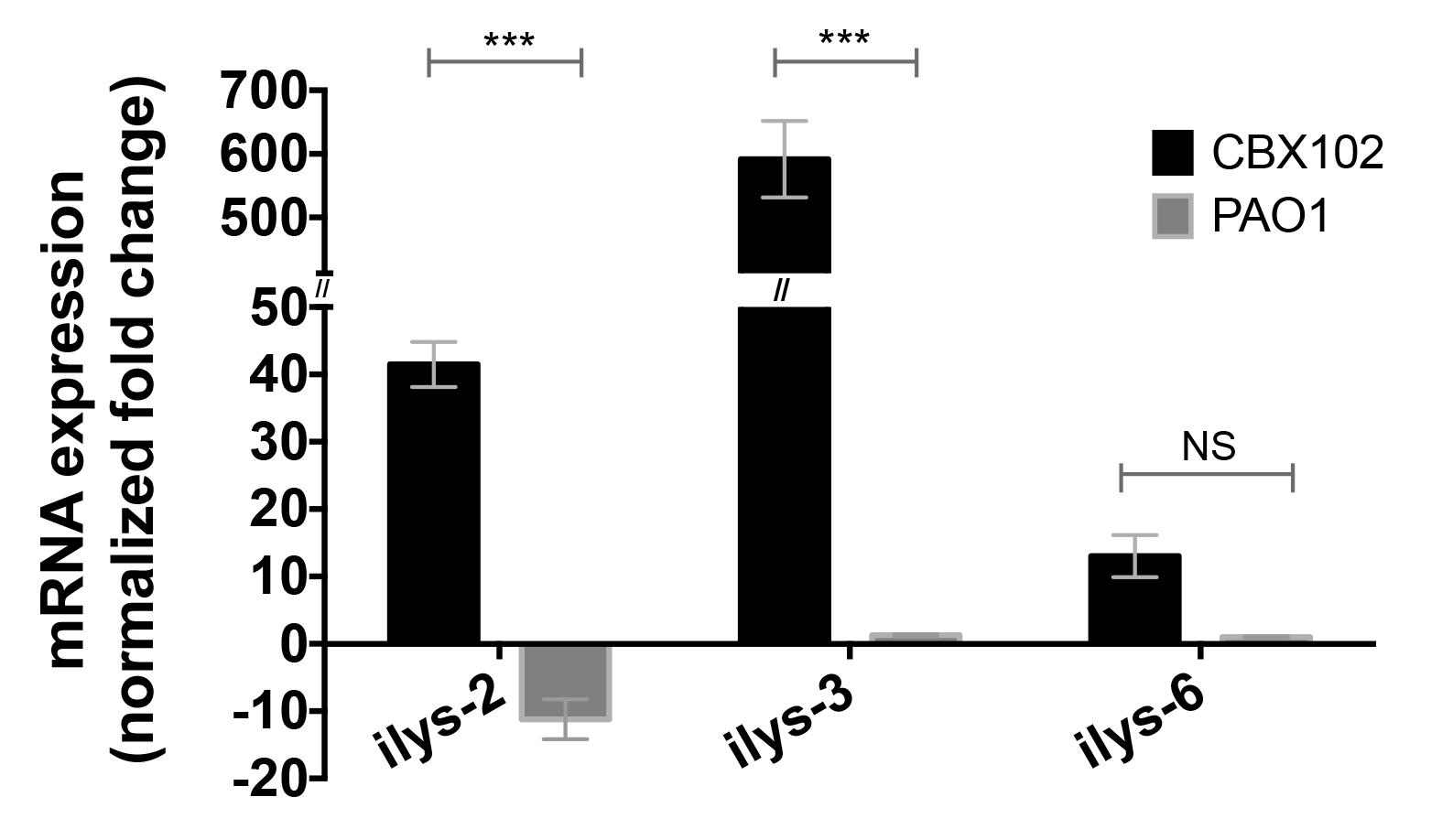

Supplement: S4 Fig — Graph shows the relative induction of ilys-2, -3 and -6 expression following exposure to OP50, CBX102 and P. aeruginosa (PAO1) for 24 hours. mRNA levels were normalized to E. coli OP50, and to the endogenous control gene rla-1. ilys-2 and ilys-3 transcripts, and to a less extent ilys-6, were responsive to M. nematophilum CBX102 but not to P. aeruginosa PAO1. Gene expression was analyzed using the comparative ΔΔCt method. Data are representative of 2 independent experiments. Error bars denote SEM. ***: indicate statistically significant differences of the indicated comparisons. Data were analyzed with two-way Anova, Holm-Sidak's multiple comparison tests (99% CI). CBX102 induced significantly higher levels of ilys-2 and -3 than PAO1 (*** p < 0.0001). In contrast, expression levels of ilys-6 were not significantly different (p = 0.2876, NS). (TIF) [file ppat.1005826.s004.tif]

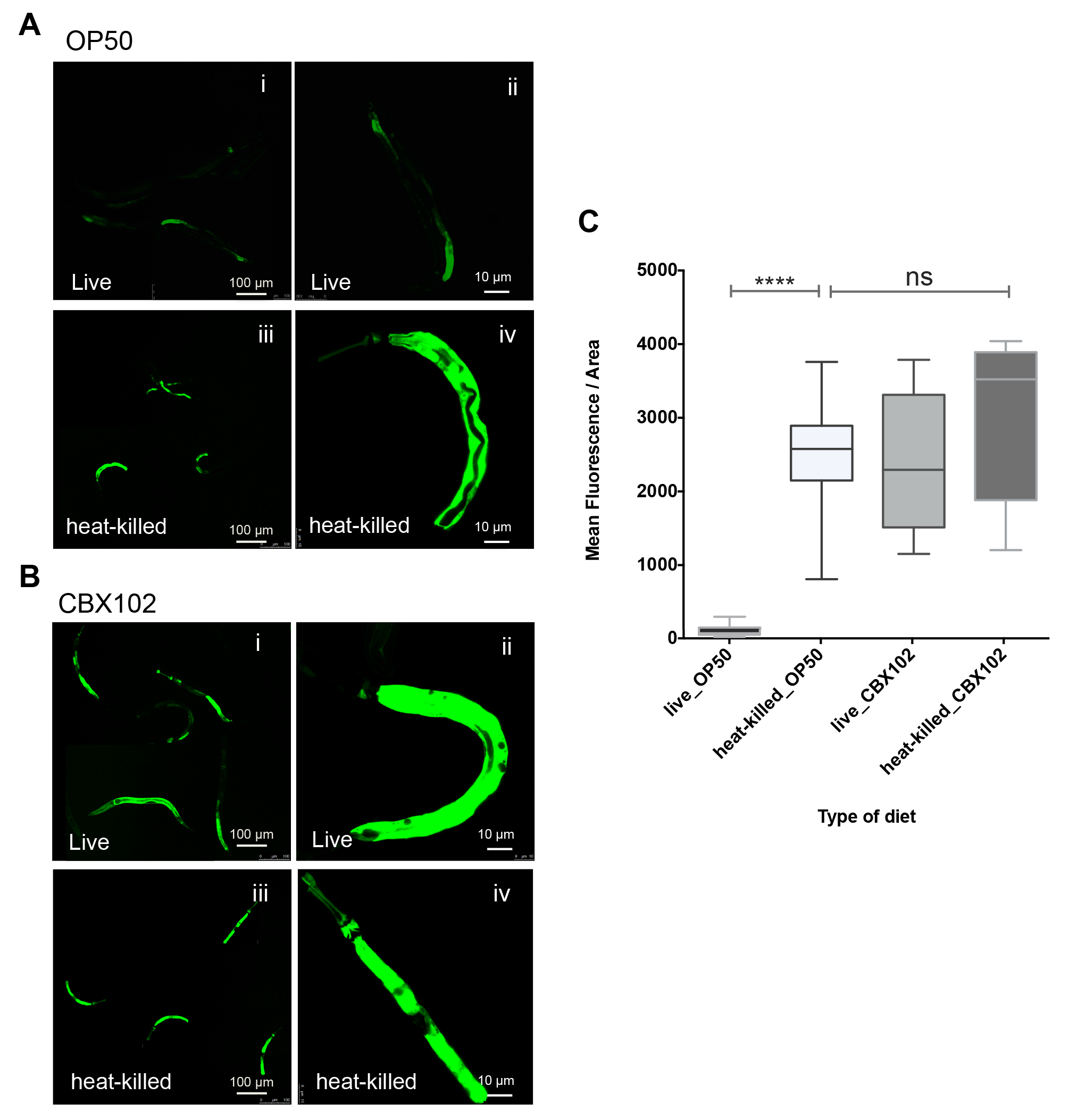

Supplement: S5 Fig — (A-B) Images of ilys-3 reporter animals fed on live or dead bacteria for 48 hours, after bleaching. Worms were added to NGM plates as synchronized L1 larvae. (A) Details of the GFP expression in animals grown on OP50. (i) and (ii) basal ilys-3p::GFP expression in worms on live OP50. (iii-iv) Strong induction in animals on dead OP50. (B) Details of GFP induction on CBX102. (i-ii) High GFP expression in the intestine of animals on live bacterial cells as well as (iii-iv) on dead CBX102. (C) Quantification of the ilys-3p::GFP fluorescence in the intestinal cell int2 of the ilys-3 reporter in animals fed on live or heat-killed OP50 or CBX102 for 48 hours. ROI was set to 20 μ diameter and 0.4 μ thickness. Graph is representative of two independent experiments. Asterisks indicate the results of Mann Whitney test of fluorescence values, 99% confidence interval. Fluorescence intensity for worms on heat-killed_OP50 vs live_OP50 differ significantly (****p < 0.0001). Mean values for animal heat-killed_CBX102 vs live_CBX102 and heat-killed_OP50 vs heat-killed_CBX102 were not significantly different (p = 0.0715 and p = 0.0717, respectively). NS: not significant. N = 12 per group. (TIF) [file ppat.1005826.s005.tif]

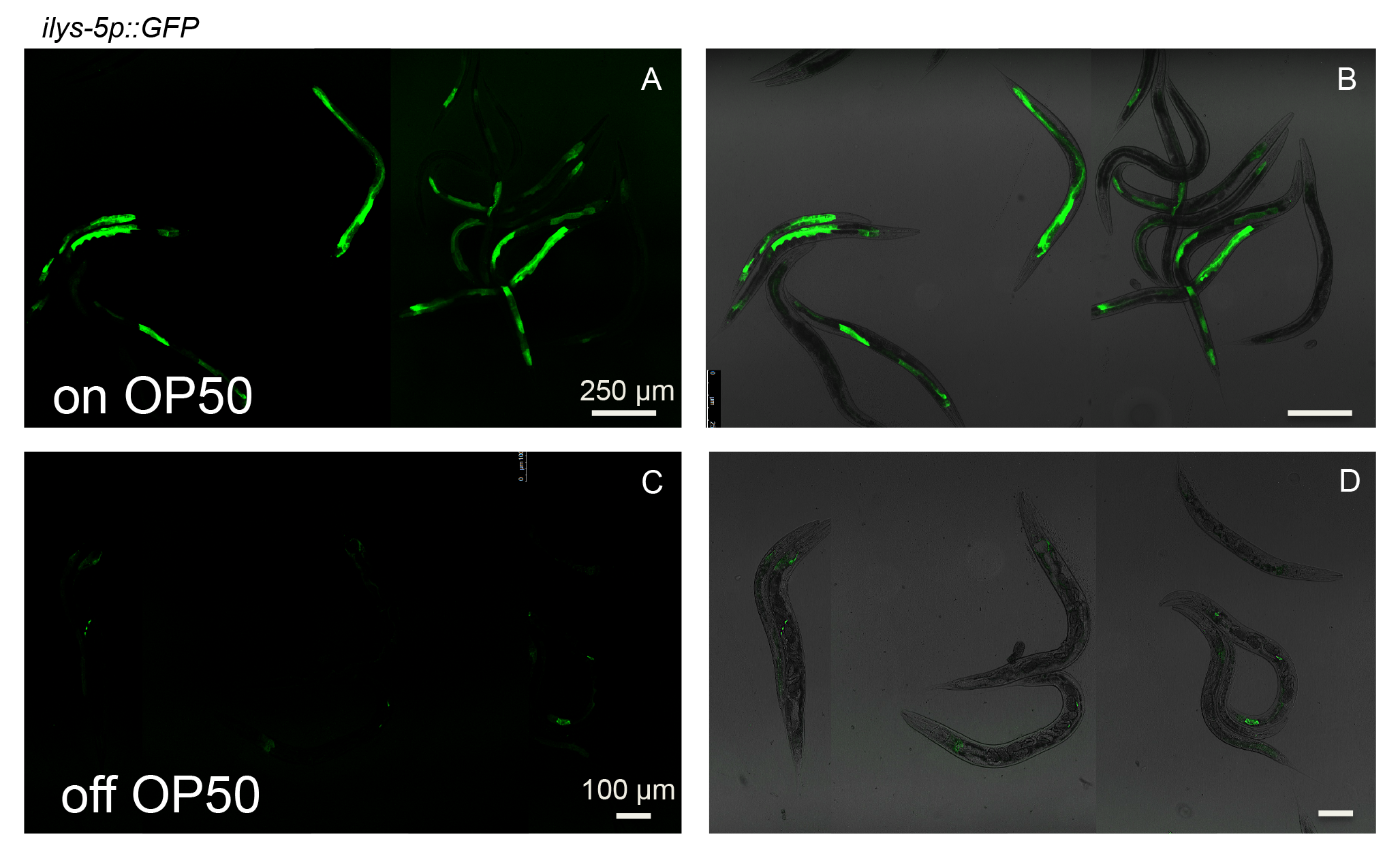

Supplement: S6 Fig — (A-B) One-day old adult animals carrying the ilys-5p::GFP reporter and grown on OP50. Basal levels of GFP expression can be detected in their intestines. (C-D) Fluorescent images of representative transgenic animals that were transferred to nutrient-depleted plates at L4 and imaged 24 hours latter. The high intestinal ilys-5p::GFP signal is almost completely abrogated in worms grown in the absence of bacteria. (TIF) [file ppat.1005826.s006.tif]

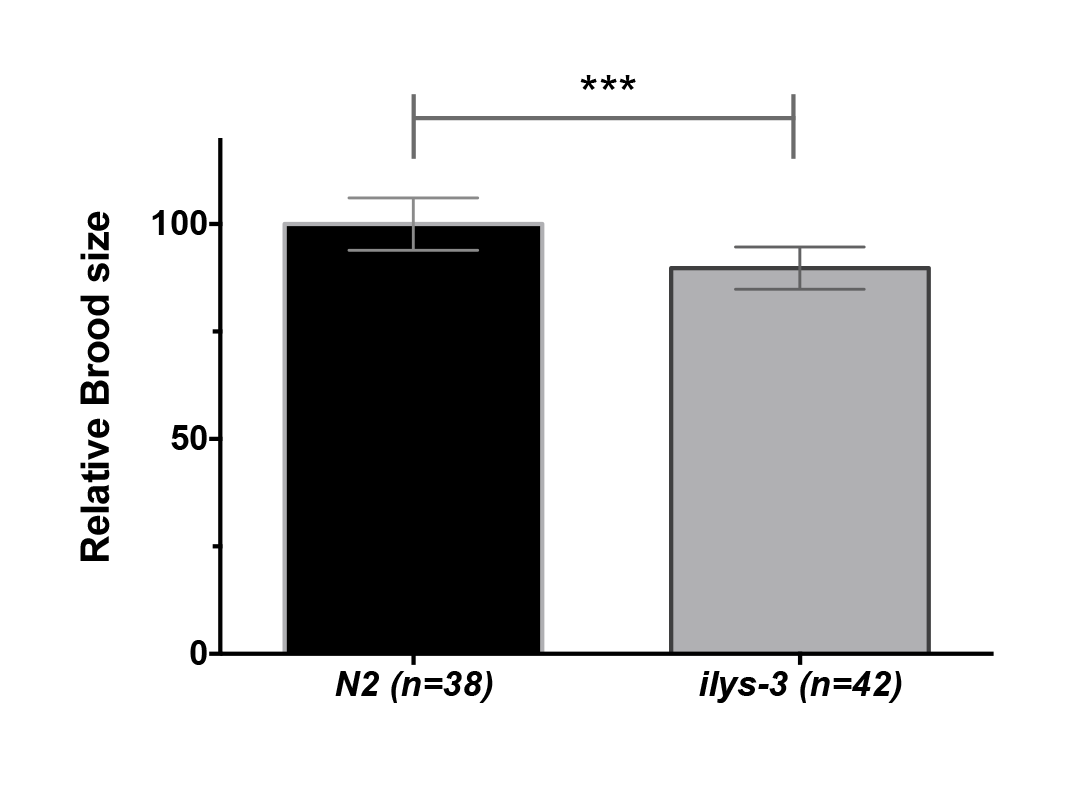

Supplement: S7 Fig — Total number of progeny counted for individual wild-type (N2) or ilys-3 mutants. Average brood sizes are expressed as a percentage of wild-type (error bars indicate SEM). Asterisks indicate ***p < 0.0001 (two-tailed unpaired t-test). Loss of ilys-3 reduced the brood by 10% only. [Broods: N2 = 269 vs ilys-3 = 241]. (TIF) [file ppat.1005826.s007.tif]

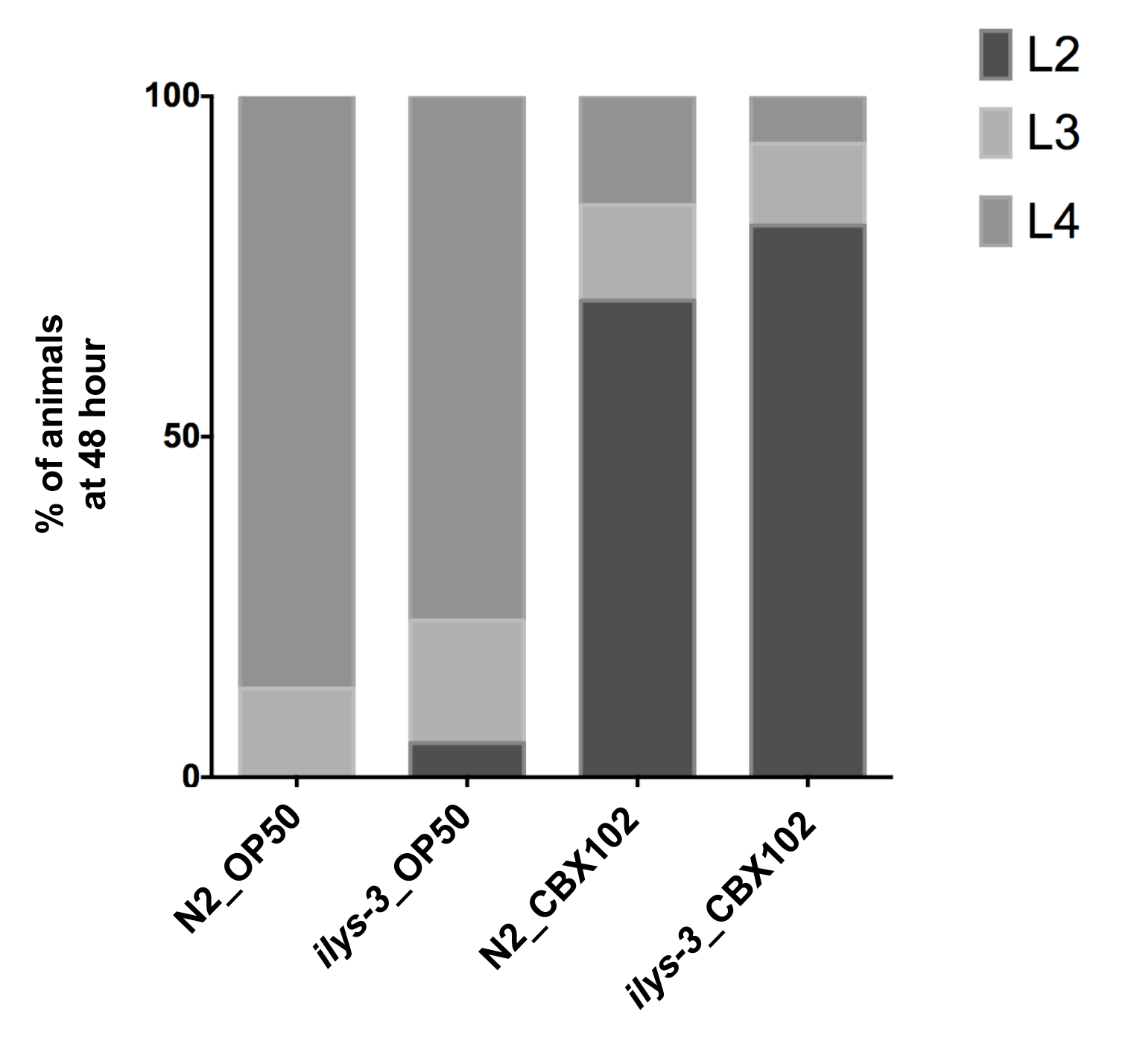

Supplement: S8 Fig — Plot represents developmental progression on live cells of OP50 and CBX102. Synchronized N2 and ilys-3 animals (at L1 stage) were grown on the two different diets as indicated on the x-axis, and scored after 48 hours. (TIF) [file ppat.1005826.s008.tif]

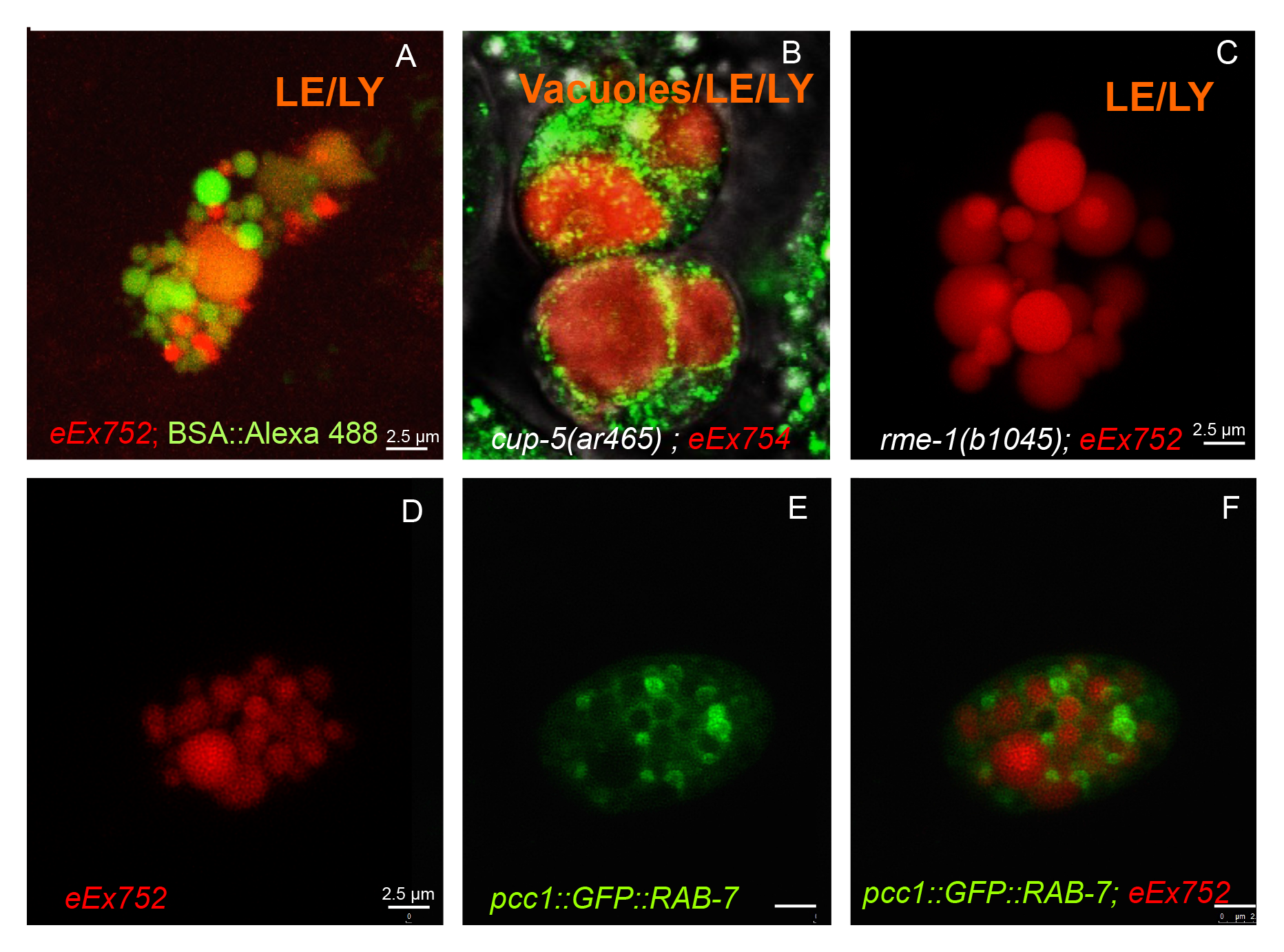

Supplement: S9 Fig — (A) ILYS-3::mCherry and BSA::Alexa 488 colocalization. (B) Vacuoles accumulating ILYS-3::mCherry in the cup-5(ar465) mutant. Green shows endocytosed GFP (myo-3p::ssGFP). (C) Deficient endocytosis does not block ILYS-3::mCherry in the coelomocytes in the rme-1(b1045) mutant. (D-F) Some vesicles ILYS-3::mCherry-labelled colocalize with the RAB-7 GFP-marked late endosomes and lysosomes. (D) Red channel. (E) Green channel. (F) Overlay of the images of the corresponding red and green channels. (TIF) [file ppat.1005826.s009.tif]

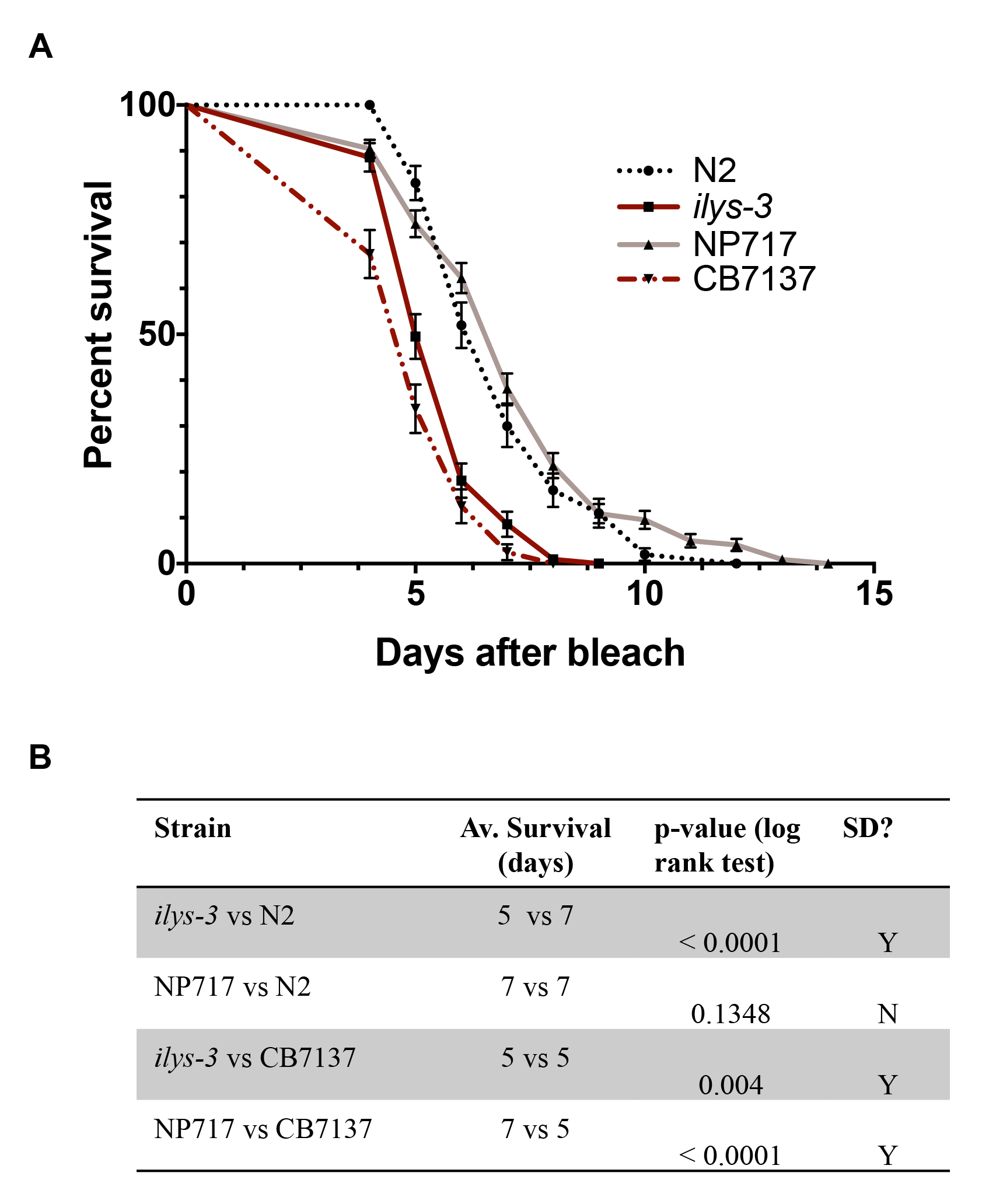

Supplement: S10 Fig — (A) Lifespan analysis of N2, ilys-3(ok3222), NP717 (coelomocyte-depleted), CB7137 [ilys-3(ok3222); coelomocyte depleted double mutants] fed on M. nematophilum from day 0 after bleaching and raised at 20°C. P value vs control calculated with the Mantel-Cox log-rank test (95% CI). Results are the mean of 2 independent trials. N ranges from 220 to 150. (B) Table summarizing the details of lifespan analysis. (TIF) [file ppat.1005826.s010.tif]

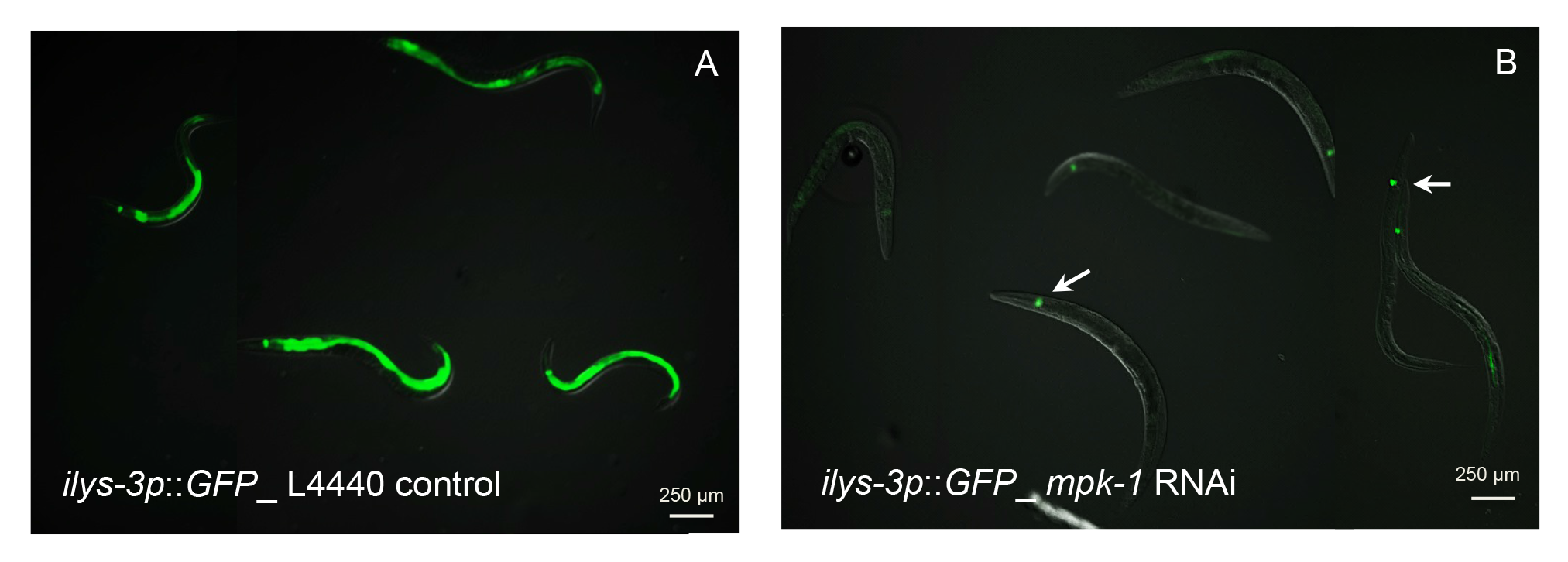

Supplement: S11 Fig — (A) Green fluorescence in transgenic worms carrying the ilys-3p::GFP reporter fed on the control RNAi L4440. (B) mpk-1 RNAi blocks the green fluorescence of the ilys-3 reporter in the intestine but not in the pharynx (arrows). (TIF) [file ppat.1005826.s011.tif]

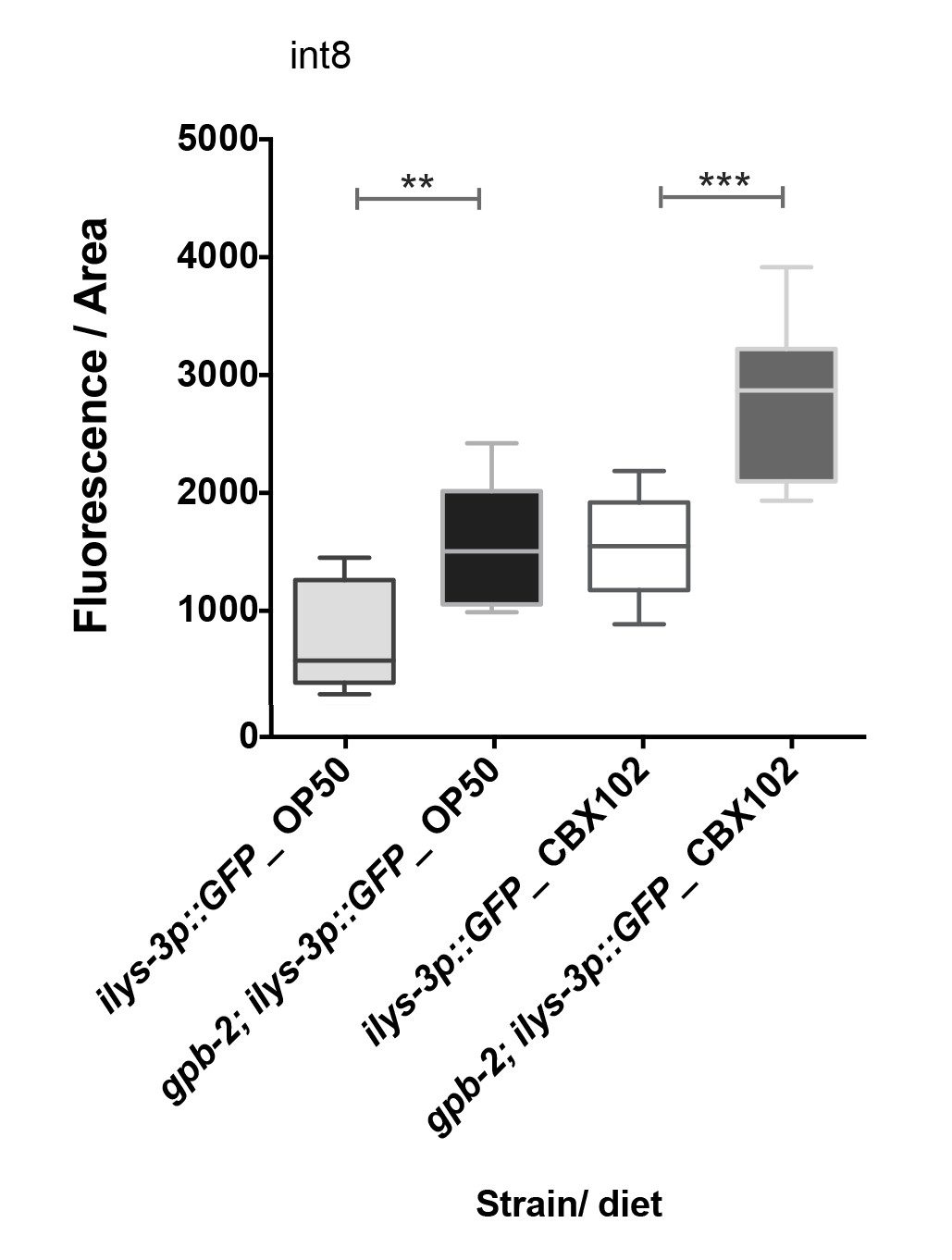

Supplement: S12 Fig — ROI set to 40 μ diameter and 0.4 μ thickness. Graph is representative of two independent experiments. Asterisks indicate the results of Mann Whitney test of fluorescence values, 95% confidence interval. Fluorescence intensity for gpb-2; ilys-3p::GFP_OP50 vs ilys-3p::GFP_OP50 and gpb-2; ilys-3p::GFP_CBX102 vs ilys-3p::GFP _CBX102 differ significantly (** p = 0.0052 and *** p = 0.0004, respectively). (TIF) [file ppat.1005826.s012.tif]

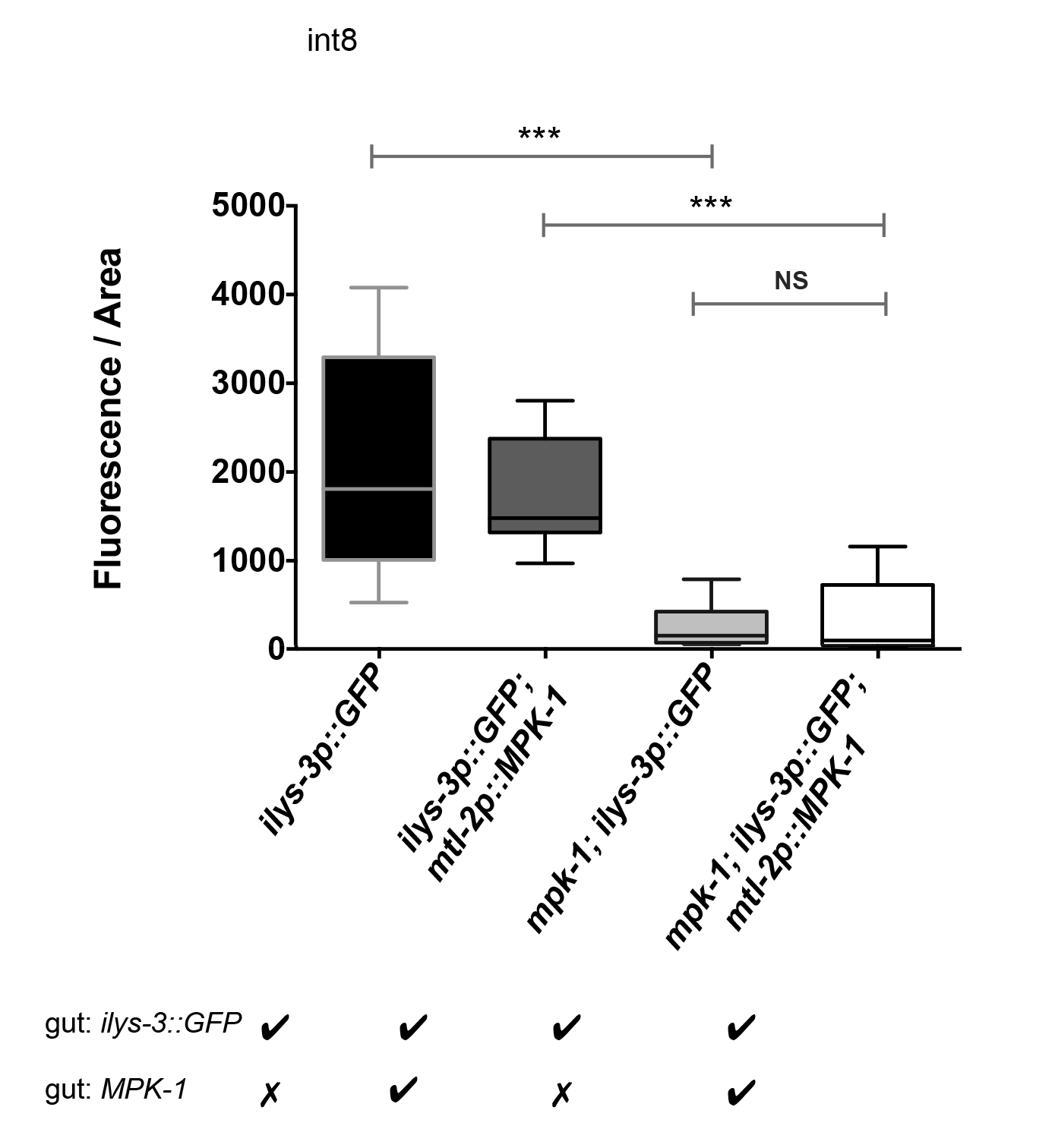

Supplement: S13 Fig — Fluorescence quantification of ilys-3 promoter activity and the effect of intestinal MPK-1 in int8. The construct mtl-2p::MPK-1 drives MPK-1 expression in the intestine. In the mpk-1 mutants, ilys-3 expression was blocked. This phenotype was not rescued when MPK-1 is restored in the intestine. Asterisks indicate the results of a Mann–Whitney Unpaired test statistical comparisons of the fluorescence intensity for mpk-1(ku1); ilys-3p::GFP; mtl-2p::MPK-1 vs ilys-3p::GFP; mtl-2p::MPK-1 and mpk-1(ku1); ilys-3p::GFP vs ilys-3p::GFP (*** p = 0.0002), mpk-1(ku1); ilys-3p::GFP; mtl-2p::MPK-1 vs ilys-3p::GFP (** p = 0.0012), which all differ from their controls. Mean values for mpk-1 mutants with the double transgene were not significantly different (NS) from their sibling controls harbouring the ilys-3p::GFP reporter only (p = 0.7137). N ≥ 15/group. (TIF) [file ppat.1005826.s013.tif]

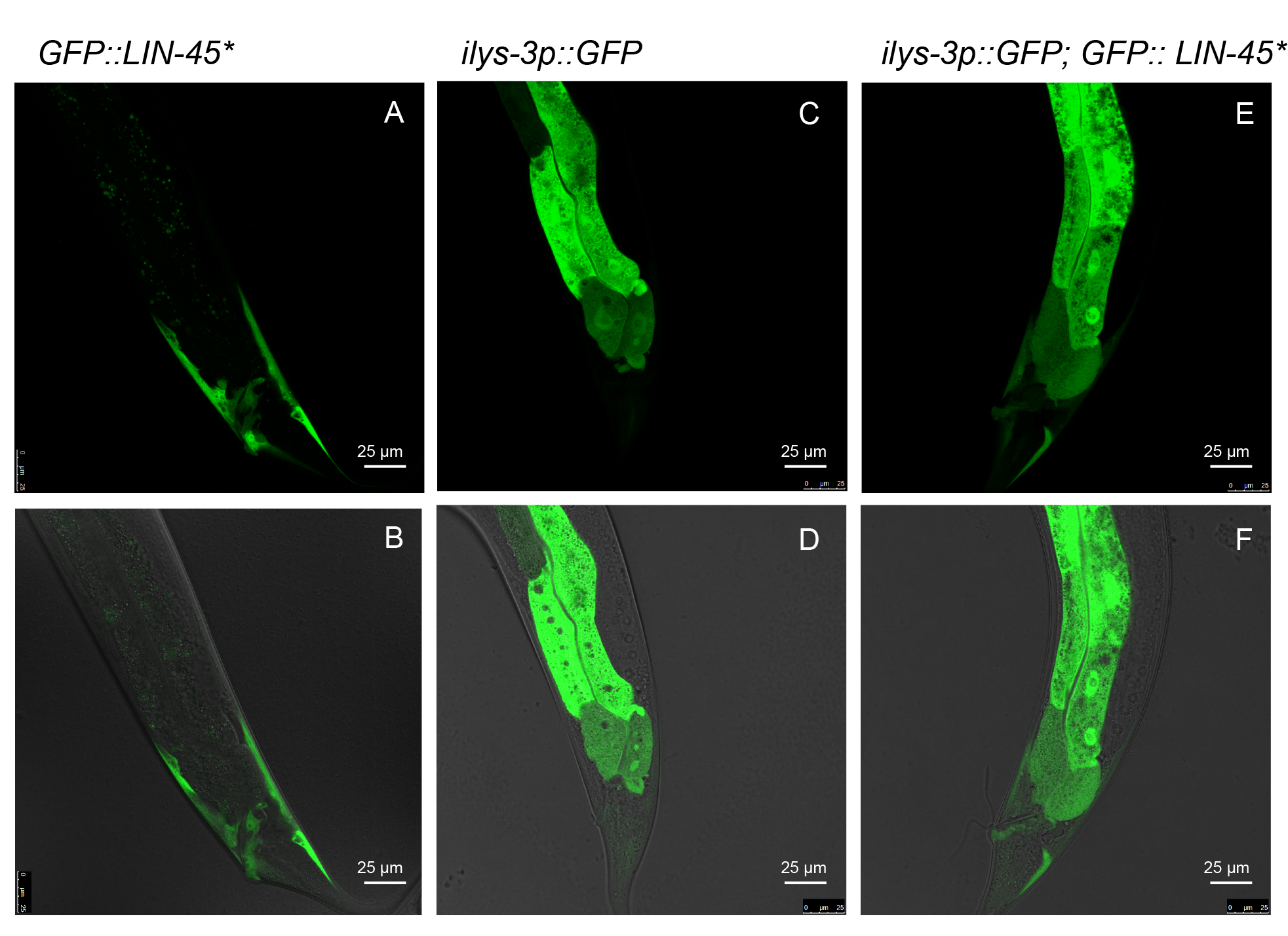

Supplement: S14 Fig — (A-B) Image of an adult showing the active LIN-45* driven by the 1.3 Kb egl-5 promoter fragment which expresses GFP in the B, K, F, U, and P12.pa rectal epithelial cells and in three posterior body wall muscles. (A) Fluorescent channel (B) Merge image of GFP and DIC. (C-D). Representative image of a transgenic animal expressing intestinal ilys-3p::GFP. (D). Overlay. (E-F). Representative image of an animal bearing the two transgenes, showing that intestinal ilys-3p::GFP expression is not enhanced by constitutively activating MAPK signalling in the rectal epithelium. (F) Merge image of GFP and DIC. All images correspond to 1-day-old adults. (TIF) [file ppat.1005826.s014.tif]

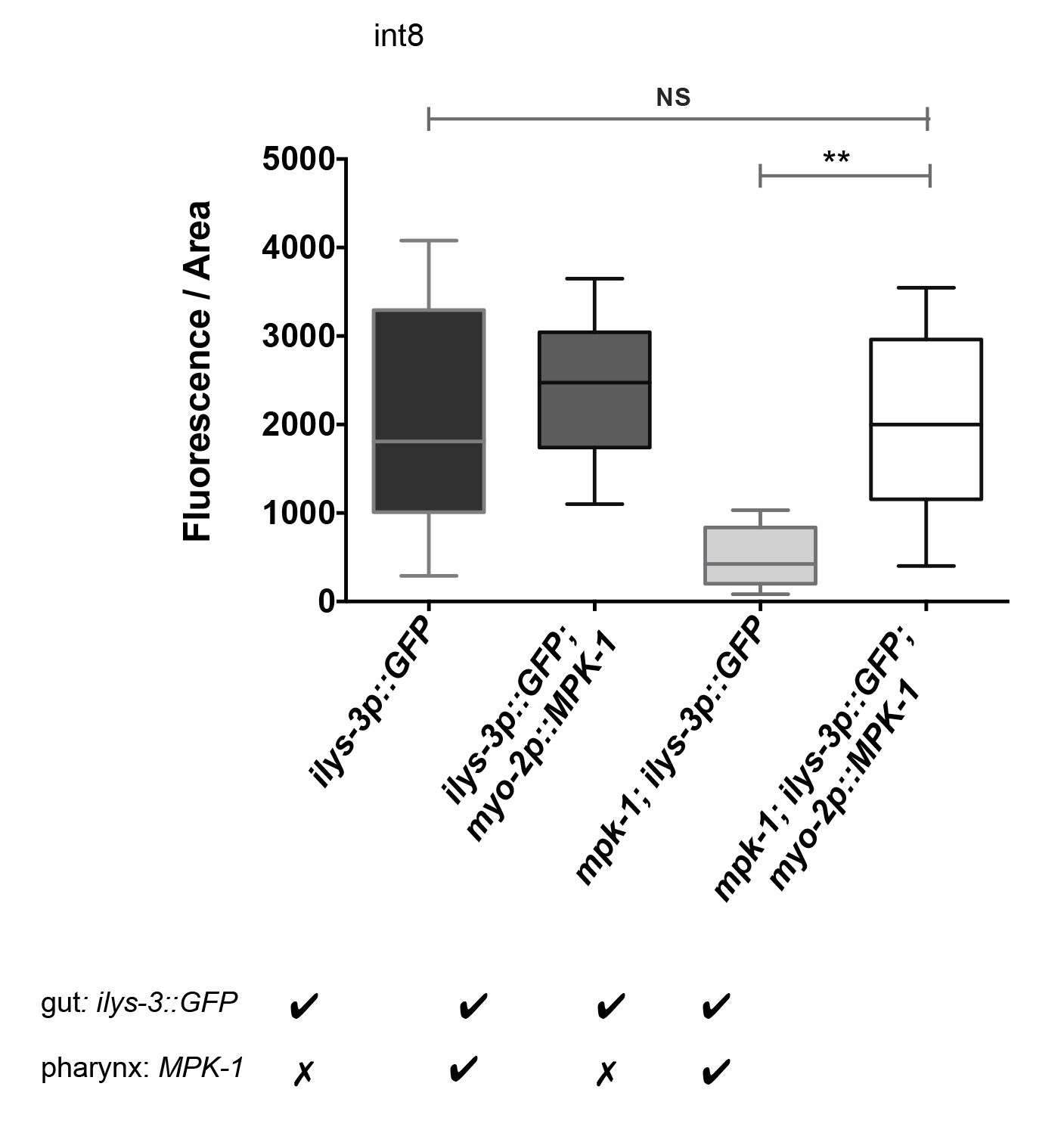

Supplement: S15 Fig — Fluorescence quantification of ilys-3 promoter activity and the effect of pharyngeal MPK-1 in int8 of single and double transgene reporter strains. The construct myo-2p::MPK-1 drives MPK-1 expression in the pharynx and rescued the intestinal ilys-3 expression in mpk-1 mutants. Data analyzed with Mann–Whitney Unpaired test, 95% confidence level. Fluorescence intensity for mpk-1(ku1); ilys-3p::GFP; myo-2p::MPK-1 vs ilys-3p::GFP; myo-2p::MPK-1 and mpk-1(ku1); ilys-3p::GFP; myo-2p::MPK-1 vs ilys-3p::GFP were not significantly different (ns). Mean values for mpk-1 mutants with the double transgene differ significantly from their sibling controls harbouring the ilys-3p::GFP reporter only (** p = 0.004). N = 10-15/ group. (TIF) [file ppat.1005826.s015.tif]

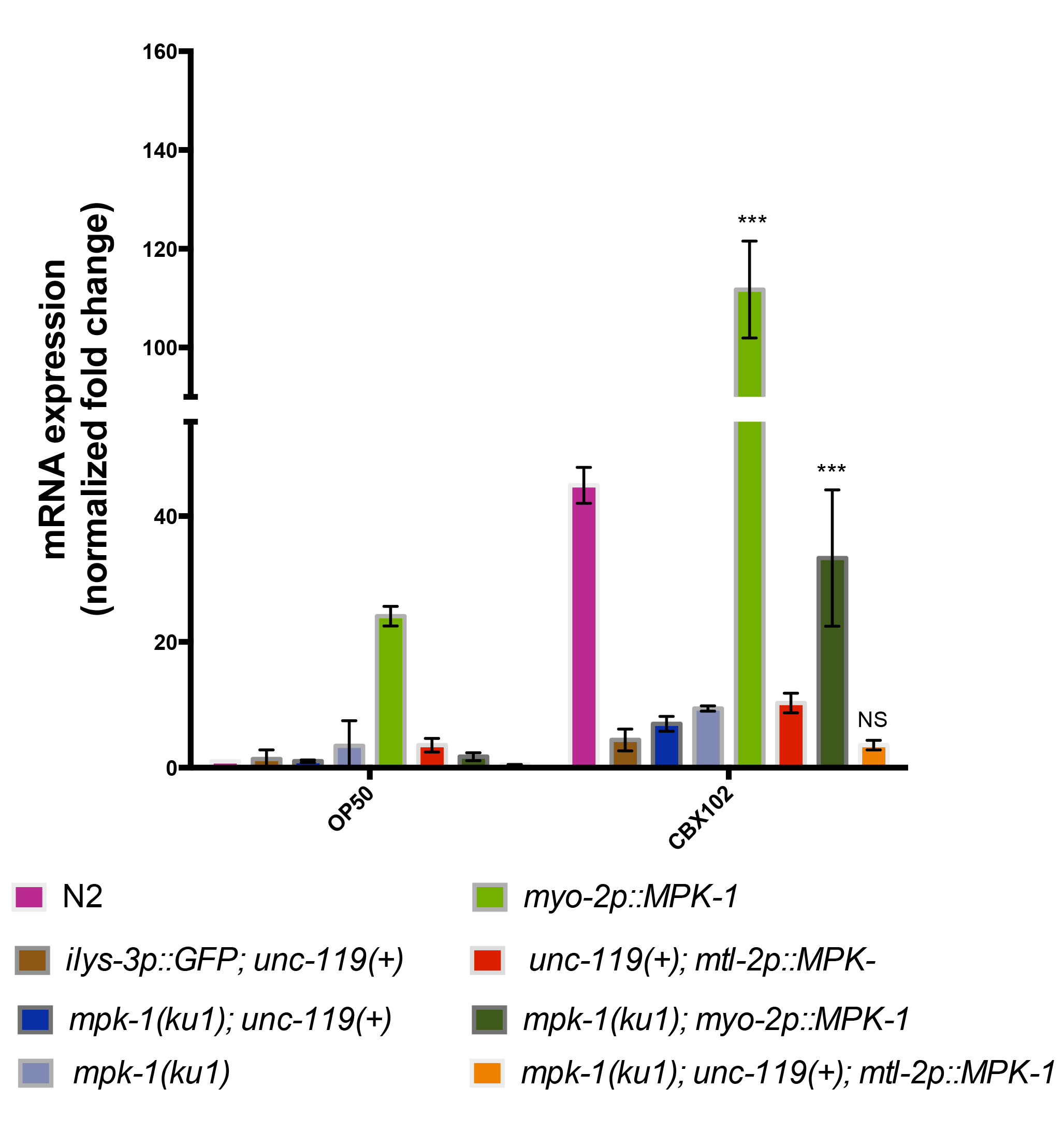

Supplement: S16 Fig — RT-PCR quantification of relative levels of ilys-3 in mpk-1(ku1) mutants and control strains grown on OP50 or CBX102. Levels were normalized against WT (N2) on E. coli and to the endogenous control gene rla-1. Gene expression was analyzed using the comparative ΔΔCt method. Each bar represents the average relative mRNA level from three independent RNA isolations obtained from synchronization L1 in populations of worms exposed for 24 hours to M. nematophilum. Control strains were as follows: N2, ilys-3p::GFP; unc-119; unc-119 (+), mpk-1(ku1); unc-119(+) [obtained from cross with mpk-1; mtl-2p::MPK-1; unc-119; unc-119(+)], and mtl-2p::MPK-1; unc-119; unc-119(+), myo-2p::MPK-1. Data were analyzed with two-way Anova, Holm-Sidak's multiple comparison tests, 99% confidence interval. Error bars represent SEM. Expression levels of ilys-3 were significantly higher in transgenic animals expressing the pharyngeal MPK-1 relative to N2 controls regardless of nature of the bacterial lawn (*** p < 0.0001). The mean values for mpk-1 mutants with the intestinal MPK-1 transgene were not significantly different from their sibling single mutant controls (NS: p > 0.9999). For multiple comparisons see S4 Table. (TIF) [file ppat.1005826.s016.tif]

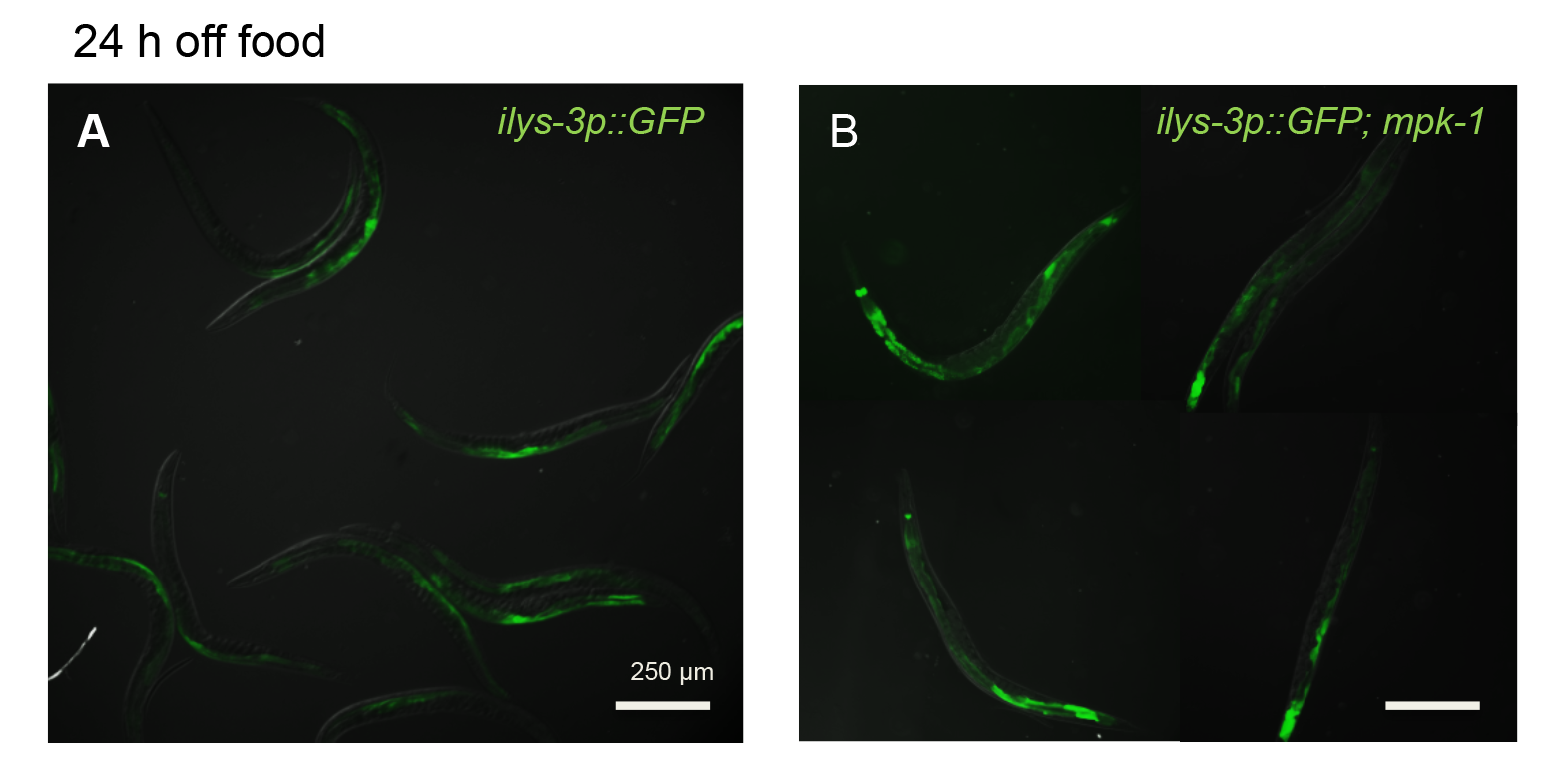

Supplement: S17 Fig — Fluorescence images of ilys-3p::GFP reporter in worms fasted for 24 hours. (A) ilys-3p::GFP control. (B) ilys-3p::GFP; mpk-1 mutants showed similar intestinal fluorescence intensity relative to control animals. (TIF) [file ppat.1005826.s017.tif]

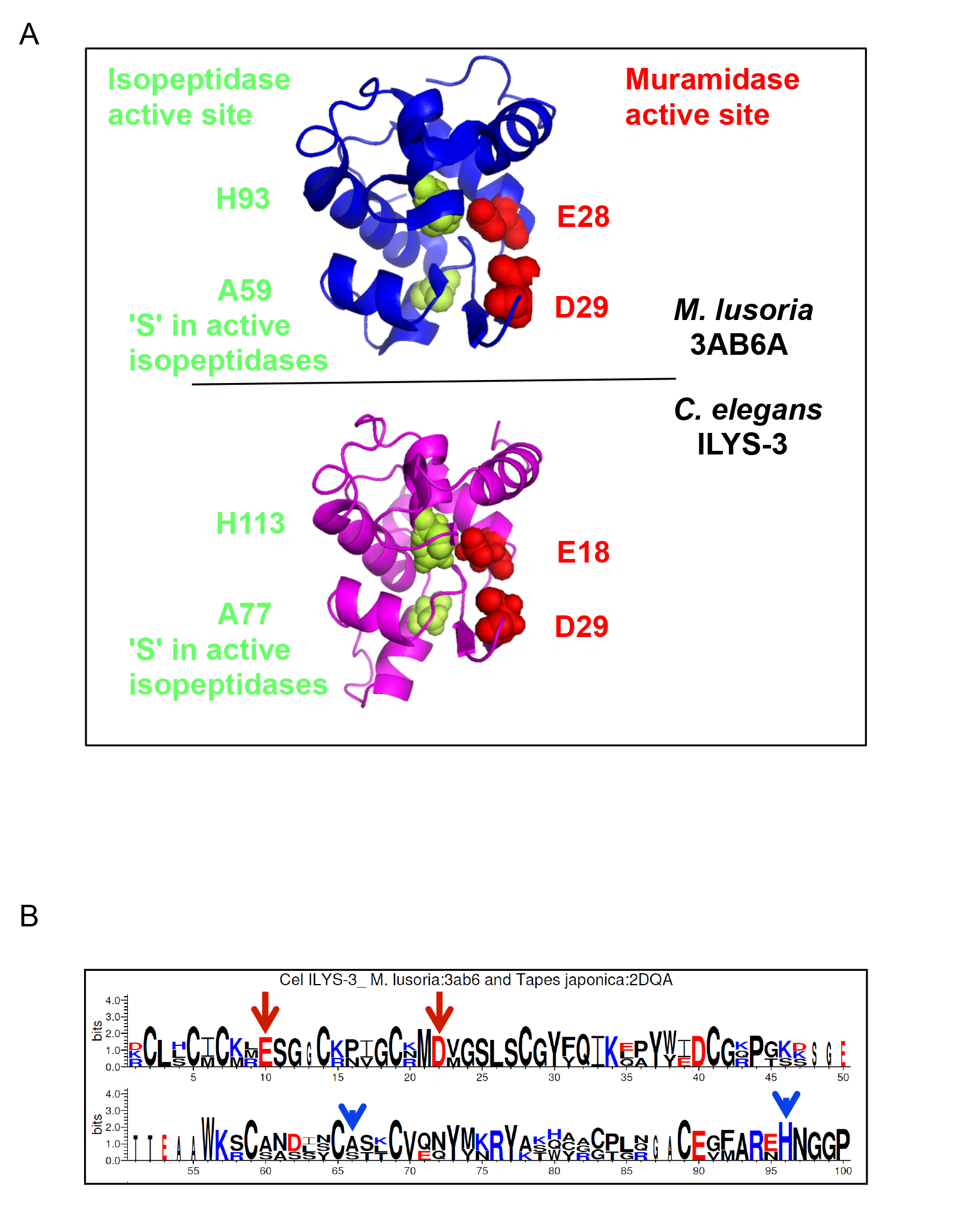

Supplement: S18 Fig — (A) Predicted 3D homology model structure of the C elegans ILYS-3 inferred from the crystal structure from of the lysozyme isolated from M. lusoria (PDB No:3AB6A). This model was created using PyMOL molecular graphic software. Residues E18 D29 predicted to be involved in muramidase activity are shown in red. Residues predicted to be responsible for the isopeptidase activity are marked in green. Of these only H113 seemed to have been conserved in both lysozymes. The other residue is a substitution of the S77 to A. (B) Sequence logo showing the conservation of amino acids in i-type lysozymes of Meretrix lusoria: 3AB6, Tapes japonica: 2DQA and C. elegans ILYS-3, based on a multiple sequence alignment (CLUSTALW) WebLogo3 was used to generate the sequence logos. Signal peptides were predicted by SignalP 4.1 server and removed prior to alignment. Poorly aligning N- and C- terminus were also removed. Amino acids are colored by charge: blue: positive and red: negative. The two muramidase residues E and D are indicated by red arrows and the possible active residues responsible for isopeptidase activity are marked with blue arrowheads. In contrast to the Tapes japonica protein, endowed with both the Serine and Histidine residues that confer isopeptidase activity, the C. elegans ILYS-3 and the M. lusoria 3AB6A appeared to have replaced the active Serine (S77) with Alanine. (TIF) [file ppat.1005826.s018.tif]
