# Supplementary material for: The Invertebrate Lysozyme Effector ILYS-3 Is Systemically Activated in Response to Danger Signals and Confers Antimicrobial Protection in C. elegans
Source: PLoS Pathog. 2016 Aug 15;12(8):e1005826. doi: 10.1371/journal.ppat.1005826 (PMC4985157; doi:10.1371/journal.ppat.1005826)
Supplement: S1 Table — (DOCX) [file ppat.1005826.s019.docx]

| **Reporter** | **Expression** |
| --- | --- |
| *ilys-1p::DsRed2* | pharyngeal muscles pm3, pm4 (sieve), and marginal cells mc1 and mc2 |
| *ilys-2p::CFP* | pharyngeal muscle pm3, nerve ring and intestine |
| *ilys-3p::GFP* | pharyngeal muscle pm7 (grinder), marginal cell mc2 and intestine |
| *ilys-4p::GFP* | interneurons and intestine |
| *ilys-5p::GFP* | interneurons, pharyngeal neuron and intestine |
| *ilys-6p::GFP* | pharyngeal gland cells, coelomocytes and intestine |
