# Supplementary material for: The Invertebrate Lysozyme Effector ILYS-3 Is Systemically Activated in Response to Danger Signals and Confers Antimicrobial Protection in C. elegans
Source: PLoS Pathog. 2016 Aug 15;12(8):e1005826. doi: 10.1371/journal.ppat.1005826 (PMC4985157; doi:10.1371/journal.ppat.1005826)
Supplement: S2 Table — P value vs control calculated with the Mantel-Cox log-rank test (95% CI). Results are the mean of 3 independent trials. (DOCX) [file ppat.1005826.s020.docx]

| **Strain** | **Mean Survival ± SEM** | ***p*-value (log rank test)** | **SD curves?** |
| --- | --- | --- | --- |
| *ilys-3 vs* N2 | **12.50** ± 0.1000 | < 0.0001 | Y |
|  | vs |  |  |
|  | **16.50** ± 0.0527 |  |  |
| *ilys-3; eEx752 vs ilys-3* | **15.5** ± 0.5000 | < 0.0001 | Y |
|  | *vs* |  |  |
|  | **12.50** ± 0.1000 |  |  |
| *ilys-3; eEx754 vs ilys-3* | **15.50** ± 0.0916 | < 0.0001 | Y |
|  | *vs* |  |  |
|  | **12.50** ± 0.1000 |  |  |
| *+; eEx754 vs* N2 | **16.50** ± 0.1225 | 0.6581 | N |
|  | *vs* |  |  |
|  | **16.50** ± 0.0527 |  |  |
