# Supplementary material for: The Invertebrate Lysozyme Effector ILYS-3 Is Systemically Activated in Response to Danger Signals and Confers Antimicrobial Protection in C. elegans
Source: PLoS Pathog. 2016 Aug 15;12(8):e1005826. doi: 10.1371/journal.ppat.1005826 (PMC4985157; doi:10.1371/journal.ppat.1005826)
Supplement: S3 Table — P value vs control calculated with the Mantel-Cox log-rank test (95% CI). Results are the mean of 3 independent trials. (DOCX) [file ppat.1005826.s021.docx]

| **Strain** | **Mean Survival ± SEM** | ***p-value (log rank test)*** | **SD curves ?** |
| --- | --- | --- | --- |
| *ilys-3 vs* N2 | **6.5 ± 0.3180** | < 0.0001 | Y |
|  | vs |  |  |
|  | **9.5 ± 0.5487** |  |  |
| *ilys-3; eEx752 vs ilys-3* | **10.9 ± 0.7621** | < 0.0001 | Y |
|  | *vs* |  |  |
|  | **6.5 ± 0.3180** |  |  |
| *ilys-3; eEx754 vs ilys-3* | **9.6 ± 0.2963** | < 0.0001 | Y |
|  | *vs* |  |  |
|  | **6.5 ± 0.3180** |  |  |
| *ilys-3; eEx752 vs* N2 | **10.9 ± 0.7621** | 0.0002 | Y |
|  | *vs* |  |  |
|  | **9.5 ± 0.5487** |  |  |
| *ilys-3; eEx754 vs* N2 | **9.6 ± 0.2963** | 0.024 | Y |
|  | *vs* |  |  |
|  | **9.5 ± 0.5487** |  |  |
| *+; eEx754 vs* N2 | **9.6 ± 0.8490** | 0.0004 | Y |
|  | *vs* |  |  |
|  | **9.5 ± 0.5487** |  |  |
