# Supplementary material for: The Invertebrate Lysozyme Effector ILYS-3 Is Systemically Activated in Response to Danger Signals and Confers Antimicrobial Protection in C. elegans
Source: PLoS Pathog. 2016 Aug 15;12(8):e1005826. doi: 10.1371/journal.ppat.1005826 (PMC4985157; doi:10.1371/journal.ppat.1005826)
Supplement: S4 Table — (DOCX) [file ppat.1005826.s022.docx]

| **Strain** | **Significant?** | **Summary** | **Adjusted P Value** |
| --- | --- | --- | --- |
| **N2_CBX102 *vs. myo-2p::MPK-1_*CBX102** | **Yes** | ******** | **< 0.0001** |
| **N2_CBX102 *vs.* *mpk-1(ku1); unc-119*(+)_CBX102** | **Yes** | ******** | **< 0.0001** |
| **N2_CBX102 *vs. mpk-1(ku1)* _CBX102** | **Yes** | ******** | **< 0.0001** |
| N2_ CBX102 *vs.* *mpk-1(ku1)*; *myo-2p::MPK-1­*_CBX102 | No | ns | 0.9283 |
| **N2 _CBX102 *vs.* *mpk-1(ku1); unc-119(*+); *mtl-2p::MPK-1*_CBX102** | **Yes** | ******** | **< 0.0001** |
| ***myo-2p::MPK-1*_CBX102 *vs. unc-119*(+); *mtl-2p::MPK-1*_CBX102** | **Yes** | ******** | **< 0.0001** |
| *unc-119(+); mtl-2p::MPK-1*_ CBX102 *vs.* *mpk-1(ku1)*; *unc-119*(+)_ CBX102 | No | ns | > 0.9999 |
| *mpk-1(ku1); unc-119*(+)_ CBX102 *vs. mpk-1(ku1)* _ CBX102 | No | ns | > 0.9999 |
| ***mpk-1(ku1); unc-119*(+)_CBX102 *vs.* *mpk-1(ku1)*; *myo-2p::MPK-1*_CBX102** | **Yes** | ****** | **0.002** |
| *mpk-1(ku1); unc-119*(+)_ CBX102 *vs.* *mpk-1(ku1); unc-119*(+); *mtl-2p::MPK-1*_ CBX102 | No | ns | > 0.9999 |
| ***mpk-1(ku1)* _CBX102 *vs.* *mpk-1(ku1)*; *myo-2p::MPK-1*_CBX102** | **Yes** | ****** | **0.0074** |
| *mpk-1(ku1)* _CBX102 *vs. mpk-1(ku1); unc-119*(+); *mtl-2p::MPK-1*_CBX102 | No | ns | > 0.9999 |
| ***mpk-1(ku1)*; *myo-2p::MPK-1*_CBX102 *vs*. *mpk-1(ku1)*; *unc-119*(+); *mtl-2p::MPK-1*_CBX102** | **Yes** | ******* | **0.0003** |
| **N2_ OP50 *vs.* N2_CBX102** | **Yes** | ******** | **< 0.0001** |
| N2_OP50 *vs.* *myo-2p::MPK-1*_OP50 | Yes | * | 0.0112 |
| ***myo-2p::MPK-1*_ OP50 vs. *myo-2p::MPK-1*_CBX102** | **Yes** | ******** | **< 0.0001** |
| *mpk-1(ku1); unc-119*(+)_OP50 *v* *vs.* *mpk-1(ku1)* _OP50 | No | ns | > 0.9999 |
| *mpk-1(ku1); unc-119*(+)_OP50 *vs.* *mpk-1(ku1); unc-119*(+)_CBX102 | No | ns | > 0.9999 |
| *mpk-1(ku1); unc-119*(+)_OP50 *vs. mpk-1(ku1)*_CBX102 | No | ns | 0.9997 |
| ***mpk-1(ku1); unc-119*(+)_ OP50 *vs*. *mpk-1(ku1)*; *myo-2p::MPK-1*_CBX102** | **Yes** | ******** | **< 0.0001** |
| *mpk-1(ku1); unc-119*(+)_OP50 *vs.* *mpk-1(ku1); unc-119*(+); *mtl-2p::MPK-1*_CBX102 | No | ns | > 0.9999 |
| *mpk-1(ku1)* _OP50 *vs. mpk-1(ku1); unc-119*(+)_CBX102 | No | ns | > 0.9999 |
| *mpk-1(ku1)* _OP50 *vs.* *mpk-1(ku1)* _CBX102 | No | ns | > 0.9999 |
| ***mpk-1(ku1)* _ OP50 *vs.* *mpk-1(ku1)*; *myo-2p::MPK-1*_CBX102** | **Yes** | ******* | **0.0003** |
| *mpk-1(ku1)* _OP50 *vs*. *mpk-1(ku1); unc-119*(+) *mtl-2p::MPK-1*_CBX102 | No | ns | > 0.9999 |
| ***mpk-1(ku1)*; *myo-2p::MPK-1* _ OP50 *vs. myo-2p::MPK-1*_CBX102** | **Yes** | ******** | **< 0.0001** |
| *mpk-1(ku1)*; *myo-2p::MPK-1*_OP50 *vs*. *mpk-1(ku1); unc-119*(+); *mtl-2p::MPK-1*_CBX102 | No | ns | > 0.9999 |
| ***mpk-1(ku1); unc-119*(+);*mtl-2p::MPK-1*_ OP50 *vs. myo-2p::MPK-1*_CBX102** | **Yes** | ******** | **< 0.0001** |
| *mpk-1(ku1); unc-119*(+); *mtl-2p::MPK-1*_OP50 *vs*. *mpk-1(ku1); unc-119*(+)_CBX102 | No | ns | > 0.9999 |
