# Supplementary material for: The Invertebrate Lysozyme Effector ILYS-3 Is Systemically Activated in Response to Danger Signals and Confers Antimicrobial Protection in C. elegans
Source: PLoS Pathog. 2016 Aug 15;12(8):e1005826. doi: 10.1371/journal.ppat.1005826 (PMC4985157; doi:10.1371/journal.ppat.1005826)
Supplement: S5 Table — (DOCX) [file ppat.1005826.s023.docx]

| **Strain** | **Genotype** |
| --- | --- |
| CB6710 | *unc-119(ed3) III; eEx650[1.0 Kb ilys-3p::GFP + unc-119(+)] line 1* |
| CB6711 | *unc-119(ed3) III; eEx651[1.0 Kb ilys-3p::GFP+ unc-119(+)] line 2* |
| CB6712 | *unc-119(ed3) III; eEx652[1.7 Kb ilys-1p::DsRed2 + unc-119(+)] line 1* |
| CB6713 | *unc-119(ed3) III; eEx653[1.7 Kb ilys-1p::DsRed2 + unc-119(+)] line 2* |
| CB6725 | *unc-119(ed3) III; eEx655[2.4 Kb ilys-2p::CFP + unc-119(+)]* |
| CB6785 | *unc-119(ed3) III; eEx670[3.3 Kb ilys-4p::GFP + unc-119(+)] line 1* |
| CB6786 | *unc-119(ed3) III; eEx671[4.9 Kb ilys-5p::GFP + unc-119(+)] line 1* |
| CB6789 | *unc-119(ed3) III; eEx672[4.9 Kb ilys-5p::GFP + unc-119(+)] line 2* |
| CB6873 | *unc-119(ed3) III; eEx650[ilys-3p::GFP + unc-119(+)]; him-5(e1490)* |
| CB6875 | *mpk-1(ku1) unc-119(ed3) III; eEx650[ilys-3p::GFP + unc-119(+)]* |
| CB6876 | *unc-119(ed3) III; eEx673[ilys-5p::GFP + unc-119(+)]; him-5(e1490) V* |
| CB6898 | *mpk-1(ku1) unc-119(ed3) III; eEx650[ilys-3p::GFP + unc-119(+)] after outcross* |
| CB6899 | *mpk-1(ku1); adIs2200(myo-2p::MPK-1::GFP)* |
| CB6900 | *unc-119(ed3) III; adIs2200[myo-2p::MPK-1::GFP); eEx650(ilys-3p::GFP + unc-119(+)]* |
| CB7000 | *unc-119(ed3) III; eEx727[pMGN37(mtl-2p::MPK-1::unc-54 3'UTR); pMGN39(mtl-2p::mCherry) +*  *unc-119(+)]* |
| CB7001 | *unc-119 (ed3) III; eEx728[pMGN38(mtl-2p::MPK-1(b)long::unc-54 3'UTR); pMGN39(mtl-2p::mCherry + unc-119(+)]* |
| CB7002 | *unc-119(ed3) III; gpb-2(ad541) IV; eEx650[ilys-3p::GFP + unc-119(+)]* |
| CB7003 | *mpk-1(ku1) unc-119(ed3) III; eEx650[ilys-3p::GFP + unc-119(+)]; adIs2200(myo-2p::MPK-1::GFP)* |
| CB7004 | *mpk-1(ku1) unc-119(ed3) III; eEx727[pMGN37(mtl-2p::MPK-1::unc-54 3'UTR); pMGN39(mtl-2p::mCherry) + unc-119(+)]* |
| CB7005 | *unc-119(ed3) III; eEx650[ilys-3p::GFP + unc-119(+)]; eEx727[pMGN37(mtl-2p::MPK-1::unc-54 3'UTR); pMGN39(mtl-2p::mCherry) + unc-119(+)]* |
| CB7006 | *mpk-1(ku1) unc-119(ed3) III; eEx650[ilys-3p::GFP + unc-119(+)]; eEx727[pMGN37(mtl-2p::MPK-1::unc-54 3'UTR); pMGN39(mtl-2p::mCherry) + unc-119(+)]* |
| CB7007 | *ilys-3(ok3222) IV outcrossed 3x* |
| CB7029 | *unc-119(ed3) III; ilys-3(ok3222) IV* |
| CB7073 | *unc-119(ed3) III; ilys-3(ok3222) IV; eEx752[pMGN47(4.5 Kb ilys-3p::ILYS-3::mCherry C- terminus::unc-54 3' UTR ) + unc-119(+)]* |
| CB7074 | *unc-119(ed3) III; ilys-3(ok3222) IV; eEx753[pMGN47(4.5 Kb ilys-3p::ILYS-3::mCherry C- terminus::unc-54 3' UTR ) + unc-119(+)]* |
| CB7075 | *ilys-3(ok3222) IV; eEx754 [pMGN47(4.5 Kb ilys-3p::ILYS-3::mCherry C- terminus::unc-54 3'UTR ) + sur-5::GFP]* |
| CB7132 | *arIs37(myo-3::ssGFP) I; cup-5(ar465) III; dpy-20 (e1282) IV; eEx754[ilys-3p::ILYS-3::mCherry C- terminus::unc-54 3'UTR ) + sur-5::GFP]* |
| CB7133 | *rme-1(b1045) V; eEx752[pMGN47(4.5 Kb ilys-3p::ILYS-3::mCherry C- terminus::unc-54 3' UTR ) + unc-119(+)] line 1* |
| CB7137 | *unc-119(ed3) III; ilys-3 (ok3222) IV; arIs37(myo-3p::ssGFP); cdls32(pcc1::DT-A(E148D) + unc-119(+) + myo-2p::GFP* |
| CB7163 | *unc-119(ed3);eIs120[ilys-3P::GFP + unc-119(+)] I* |
| CB7167 | *unc-119(ed3) III; ilys-3(ok3222) IV; eEx752(ilys-3p::ILYS-3::mCherry + unc-119(+); pWIs69(vha-6p::GFP::RAB-11)* |
| CB7168 | *unc-119(ed3) III; ilys-3(ok3222) IV; eEx752 (ilys-3p::ILYS-3::mCherry + unc-119(+); pWIs170(vha-6p::GFP::RAB-7)* |
| **Strain** | **Genotype** |
| CB7210 | *unc-119(ed3) III; eEx779[4.5kb ilys-3p::GFP::ILYS-3::3'UTR + unc-119(+)]* |
| CB7212 | *unc-119(ed3) III; eEx781[4.5kb ilys-6p::GFP + unc-119(+)]* |
| CB7216 | *unc-119(ed3) III; eIs120[ilys-3p::GFP + unc-119(+)] I; him-5(e1490) V* |
| CB7450 | *eIs120 I; eIs102(egl-5p::GFP::LIN-45*)* |
| CB7451 | *unc-119(ed3) III; cdIs66[pcc1::GFP::RAB-7 + myo-2p::GFP + unc-119(+)]; eEx752* |
| CB7452 | *rab-10(ok1494) I; eEx752* |
