# Supplementary material for: The Invertebrate Lysozyme Effector ILYS-3 Is Systemically Activated in Response to Danger Signals and Confers Antimicrobial Protection in C. elegans
Source: PLoS Pathog. 2016 Aug 15;12(8):e1005826. doi: 10.1371/journal.ppat.1005826 (PMC4985157; doi:10.1371/journal.ppat.1005826)
Supplement: S6 Table — (DOCX) [file ppat.1005826.s024.docx]

| **WormBase locus** | **Ensembl Transcript ID** | **Ensembl Gene ID** | **EntrezGene ID** | **Protein (Genbank) ID** |
| --- | --- | --- | --- | --- |
| *cup-5* | *R13A5.1* | WBGene00000846 | [176074](http://www.ncbi.nlm.nih.gov/sites/entrez?cmd=search&db=gene&term=176074%5buid%5d) | CCD63610 |
| *egl-5* | *C08C3.1* | WBGene00001174 | [176093](http://www.ncbi.nlm.nih.gov/sites/entrez?cmd=search&db=gene&term=176093%5buid%5d) | AAC37166 |
| *gpb-2* | *F52A8.2* | WBGene00001680 | [172483](http://www.ncbi.nlm.nih.gov/sites/entrez?cmd=search&db=gene&term=172483%5buid%5d) | [AAK55964](http://www.ensembl.org/Caenorhabditis_elegans/Gene/Summary?db=core;g=WBGene00017691) |
| *him-5* | *D1086.4* | WBGene00001864 | [183932](http://www.ncbi.nlm.nih.gov/sites/entrez?cmd=search&db=gene&term=183932%5buid%5d) | CBW48354 |
| *ilys-1* | *C45G7.1* | [WBGene00016668](http://www.ensembl.org/Caenorhabditis_elegans/Gene/Summary?db=core;g=WBGene00004273) | [183474](http://www.ncbi.nlm.nih.gov/sites/entrez?cmd=search&db=gene&term=183474%5buid%5d) | [CCD65534](http://www.ncbi.nlm.nih.gov/protein/AFP33152) |
| *ilys-2* | *C45G7.2* | [WBGene00016669](http://www.ensembl.org/Caenorhabditis_elegans/Gene/Summary?db=core;g=WBGene00004373) | [183475](http://www.ncbi.nlm.nih.gov/sites/entrez?cmd=search&db=gene&term=183475%5buid%5d) | [CCD65530](http://www.ncbi.nlm.nih.gov/protein/CCD72158) |
| *ilys-3* | *C45G7.3* | [WBGene00016670](http://www.ensembl.org/Caenorhabditis_elegans/Gene/Summary?db=core;g=WBGene00016668) | [177033](http://www.ncbi.nlm.nih.gov/sites/entrez?cmd=search&db=gene&term=177033%5buid%5d) | [CCD65531](http://www.ncbi.nlm.nih.gov/protein/CCD65534) |
| *ilys-4* | *C55F2.2* | [WBGene00016958](http://www.ensembl.org/Caenorhabditis_elegans/Gene/Summary?db=core;g=WBGene00016669) | [183853](http://www.ncbi.nlm.nih.gov/sites/entrez?cmd=search&db=gene&term=183853%5buid%5d) | [CCD67727](http://www.ncbi.nlm.nih.gov/protein/CCD65530) |
| *ilys-5* | *F22A3.6a* | [WBGene00017691](http://www.ensembl.org/Caenorhabditis_elegans/Gene/Summary?db=core;g=WBGene00016670) | [180928](http://www.ncbi.nlm.nih.gov/sites/entrez?cmd=search&db=gene&term=180928%5buid%5d) | [CCD64578](http://www.ncbi.nlm.nih.gov/protein/CCD65531) |
| *ilys-6* | *W03D2.7* | [WBGene00020982](http://www.ensembl.org/Caenorhabditis_elegans/Gene/Summary?db=core;g=WBGene00016958) | [177164](http://www.ncbi.nlm.nih.gov/sites/entrez?cmd=search&db=gene&term=177164%5buid%5d) | [CCD66766](http://www.ncbi.nlm.nih.gov/protein/CCD67727) |
| *lin-45* | *Y73B6A.5* | WBGene00003030 | [177436](http://www.ncbi.nlm.nih.gov/sites/entrez?cmd=search&db=gene&term=177436%5buid%5d) | CDK13625 |
| [*mpk-1*](http://www.ncbi.nlm.nih.gov/protein/CCD64578) | [*F43C1.2b*](http://www.ncbi.nlm.nih.gov/protein/CCD66766) | [WBGene00003401](http://www.ensembl.org/Caenorhabditis_elegans/Gene/Summary?db=core;g=WBGene00003401) | [175545](http://www.ncbi.nlm.nih.gov/sites/entrez?cmd=search&db=gene&term=175545%5buid%5d) | [AAA18956](http://www.ncbi.nlm.nih.gov/protein/AAA18956) |
| *mtl-2* | *T08G5.10* | WBGene00003474 | [179899](http://www.ncbi.nlm.nih.gov/sites/entrez?cmd=search&db=gene&term=179899%5buid%5d) | AAA28117 |
| *myo-2* | *T18D3.4* | WBGene00003514 | [181404](http://www.ncbi.nlm.nih.gov/sites/entrez?cmd=search&db=gene&term=181404%5buid%5d) | CAA30855 |
| *rab-10* | *T23H2.5.1* | [WBGene00004273](http://www.ensembl.org/Caenorhabditis_elegans/Gene/Summary?db=core;g=WBGene00001680) | [266836](http://www.ncbi.nlm.nih.gov/sites/entrez?cmd=search&db=gene&term=266836%5buid%5d) | [AFP33152](http://www.ncbi.nlm.nih.gov/protein/AAK55964) |
| *rab-11.1* | *F53G12.1.1* | WBGene00004274 | [171601](http://www.ncbi.nlm.nih.gov/sites/entrez?cmd=search&db=gene&term=171601%5buid%5d) | CCD71701 |
| *rab-11.2* | *W04G5.2a* | WBGene00004275 | [4363014](http://www.ncbi.nlm.nih.gov/sites/entrez?cmd=search&db=gene&term=4363014%5buid%5d) | CAJ85753 |
| *rab-7* | *W03C9.3.1* | WBGene00004271 | [174834](http://www.ncbi.nlm.nih.gov/sites/entrez?cmd=search&db=gene&term=174834%5buid%5d) | [AFP33150](http://www.ensembl.org/Caenorhabditis_elegans/Gene/Summary?db=core;g=WBGene00020982) |
| *rme-1* | *W06H8.1a* | [WBGene00004373](http://www.ensembl.org/Caenorhabditis_elegans/Gene/Summary?db=core;g=WBGene00004271) | [179010](http://www.ncbi.nlm.nih.gov/sites/entrez?cmd=search&db=gene&term=179010%5buid%5d) | [CCD72158](http://www.ncbi.nlm.nih.gov/protein/AFP33150) |
| *unc-119* | *M142.1a* | WBGene00006843 | [176519](http://www.ncbi.nlm.nih.gov/sites/entrez?cmd=search&db=gene&term=176519%5buid%5d) | CAX51683 |
| *unc-54* | *F11C3.3.1* | WBGene00006789 | [259839](http://www.ncbi.nlm.nih.gov/sites/entrez?cmd=search&db=gene&term=259839%5buid%5d) | AAA28124 |
